# Sialic Acid 4‐*N*‐Piperazine and Piperidine Derivatives Bind with High Affinity to the *P. mirabilis* Sialic Acid Sodium Solute Symporter

**DOI:** 10.1002/cmdc.202200351

**Published:** 2022-10-13

**Authors:** Tiago Bozzola, Richard E. Johnsson, Ulf J. Nilsson, Ulf Ellervik

**Affiliations:** ^1^ Department of Chemistry Lund University P.O. Box 124 221 00 Lund Sweden; ^2^ Red Glead Discovery AB Medicon Village 22381 Lund Sweden

**Keywords:** Antibacterial agents, Sialic acid, Uptake inhibition, Stereoselective amination, *Proteus mirabilis*

## Abstract

In search for novel antibacterial compounds, bacterial sialic acid uptake inhibition represents a promising strategy. Sialic acid plays a critical role for growth and colonisation of several pathogenic bacteria, and its uptake inhibition in bacteria was recently demonstrated to be a viable strategy by targeting the SiaT sodium solute symporters from *Proteus mirabilis* and *Staphylococcus aureus*. Here we report the design, synthesis and evaluation of potential sialic acid uptake inhibitors bearing 4‐*N*‐piperidine and piperazine moieties. The 4‐*N*‐derivatives were obtained via 4‐*N*‐functionalization with piperidine and piperazine nucleophiles in an efficient direct substitution of the 4‐*O*‐acetate of Neu5Ac. Evaluation for binding to bacterial transport proteins with nanoDSF and ITC revealed compounds possessing nanomolar affinity for the *P. mirabilis* SiaT symporter. Computational analyses indicate the engagement of a previously untargeted portion of the binding site.

## Graphical abstract

**A new target**: Bacterial sialic acid uptake inhibition is a promising strategy for the development of antibacterial drugs. In this study, we present the design, synthesis and evaluation of sialic acid 4‐piperidine and piperazine derivatives a novel compound class, which bind the SiaT from *Proteus mirabilis* and target a different portion of the binding site.

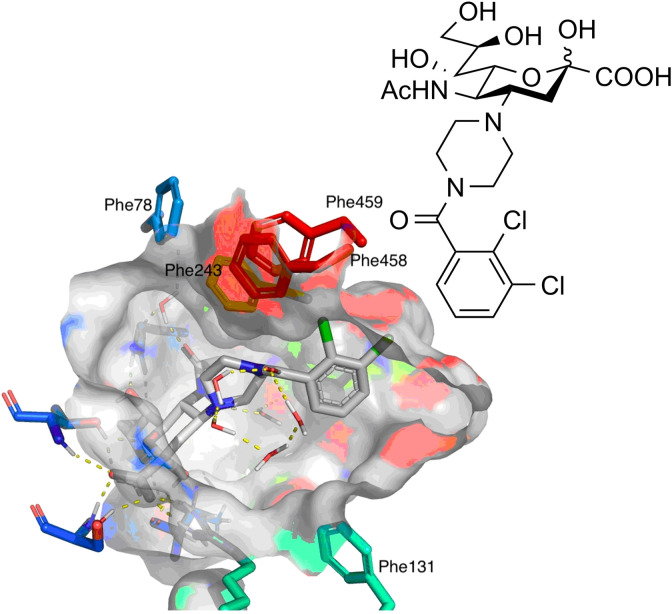

## Introduction

**Figure 1:**
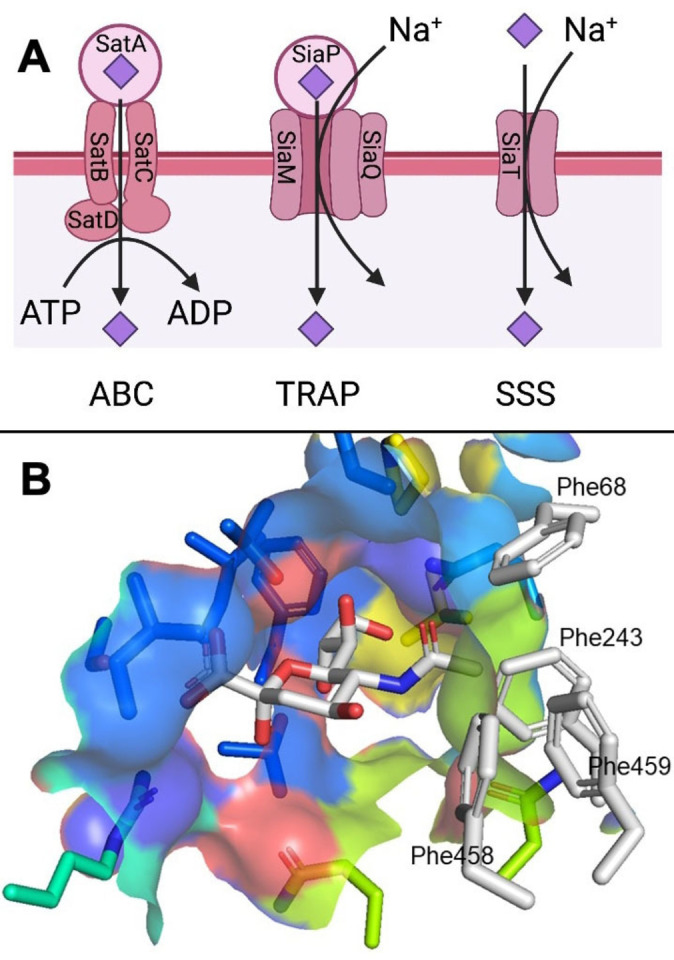
A) The three bacterial sialic acid transporter superfamilies investigated in this study, with the constituting subunits. MFS, the fourth superfamily, is not depicted. B) Neu5Ac in complex with PmSiaT (pdb ID 5NV9) with the relevant amino acids from the hydrophobic gate labelled. Colour is according to atom type with protein carbons coloured by helix. Phenyl alanine side chains of the hydrophobic gate and ligand carbons are coloured light grey.

5‐Acetyl neuraminic acid (Neu5Ac) is the most abundant member of the sialic acid family.^[1]^ Sialic acids are found at the terminal position of glycans and are crucial for many physiological and pathological processes.[^2^, ^3^, ^4^] Generally, bacteria are not able to biosynthesise sialic acid and instead harvest it from the host.^[5]^ Neu5Ac is then utilised by bacteria as a source of carbon and in a mechanism called “molecular mimicry”, in which bacteria, by sialylating their glycan epitopes, decrease recognition by our immune system. Thus, molecular mimicry favours bacterial growth and infectivity.^[6]^ We have recently developed the first example of bacterial sialic acid uptake inhibition by targeting the SiaT sodium solute symporter (SSS) from *Proteus mirabilis* (*Pm*SiaT) and *Staphylococcus aureus* (*Sa*SiaT).^[7]^ Neu5Ac 4‐*O*‐benzyl derivatives with nanomolar affinity were capable of blocking the SiaT from the two different bacterial strains in a competitive mode, as shown by a proteoliposome uptake assay. The same compounds elicited a bacterial growth delay in *S. aureus*, but not in *P. mirabilis*, in a bacterial growth model using Neu5Ac as predominant carbon source. The SSS is one of four presently known bacterial sialic acid transporter superfamilies.[^8^, ^9^] The other transporter superfamilies are the ATP binding cassette (ABC),[^10^, ^11^] the tripartite ATP‐independent periplasmic (TRAP),[^12^, ^13^] and the major facilitator superfamily (MFS).[^10^, ^14^, ^15^, ^16^] After our initial efforts on the SiaTs, we aim at the development of compounds with the capacity of targeting multiple bacterial sialic acid transporter superfamilies. Accordingly, we selected the substrate binding proteins (SBP) from the ABC and TRAP transporter superfamilies as additional targets (Figure 1a).

Specifically, we chose SatA from *Hemophilus ducreyi* (*Hd*SatA) and SiaP from *Fusobacterium nucleatum* (*Fn*SiaP) in the light of previous structural and biochemical elucidations.[^17^, ^18^] Furthermore, the two bacteria are relevant therapeutical targets. *F. nucleatum* is an opportunistic pathogenic bacterium associated with several adverse conditions such as periodontal disease, adverse pregnancy outcomes and colorectal cancer.^[19][20]^ *H. ducreyi* is a pathogenic bacterium associated with chancroid, a sexually transmitted disease in developing countries.^[21]^

**Scheme 1:**
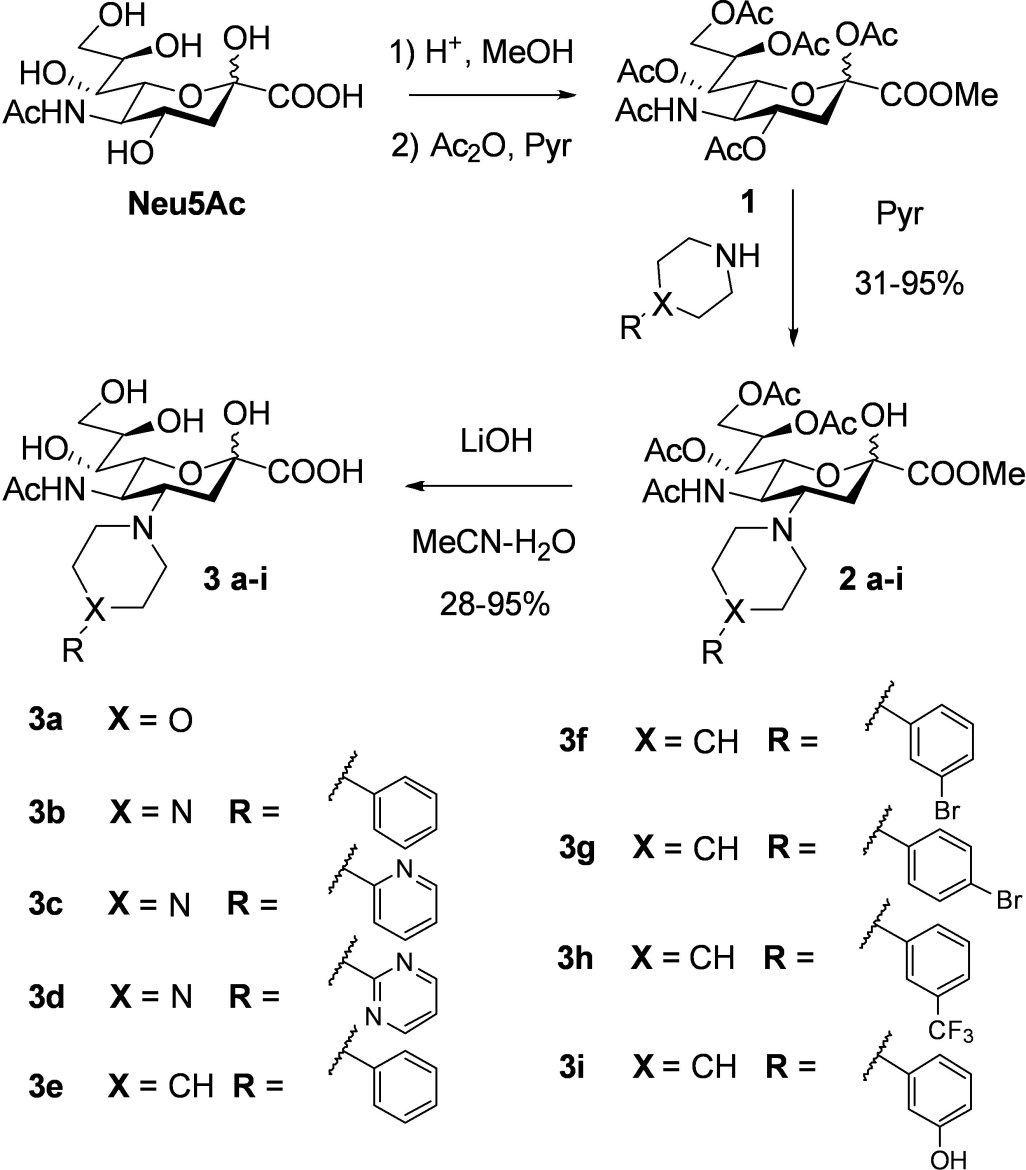
Synthetic approach in the development of compounds **3 a**‐**i**.

Based on the discovery that 4‐*O*‐benzyl ethers of sialic acid inhibit *Pm*SiaT and *Sa*SiaT,^[7]^ investigations of novel structural scaffolds at C4 of sialic acid emerged as an attractive strategy. A procedure described by Ye *et al*., and further developed by us,^[22]^ enables the introduction of cyclic secondary amines at C4 in a single step from commonly employed intermediate 1 (Scheme 1).^[23]^ We anticipated this procedure to be efficient at introducing cyclic secondary amines, specifically piperidine and piperazine at C4 of sialic acid. Piperidines and piperazines possess rigid structures that could restrain the position of the scaffold at C4 and possibly increase interactions with the hydrophobic residues of the so called hydrophobic gate, formed, among others, by Phe78, Phe243, Phe458 and Phe459 (Figure 1b).[^7^, ^24^]

Additionally, we envisioned the possibility of establishing hydrogen bonds or cation‐π interactions by the introduction of nitrogen atoms. Furthermore, piperidines and piperazines are privileged scaffolds in medicinal chemistry and appear in several marketed drugs.[^25^, ^26^] Hence, a collection of 4‐*N*‐derivatives was designed, synthesised and evaluated versus *Pm*SiaT, *Fn*SiaP, and *Hd*SatA.

## Results and Discussion

**Figure 2:**
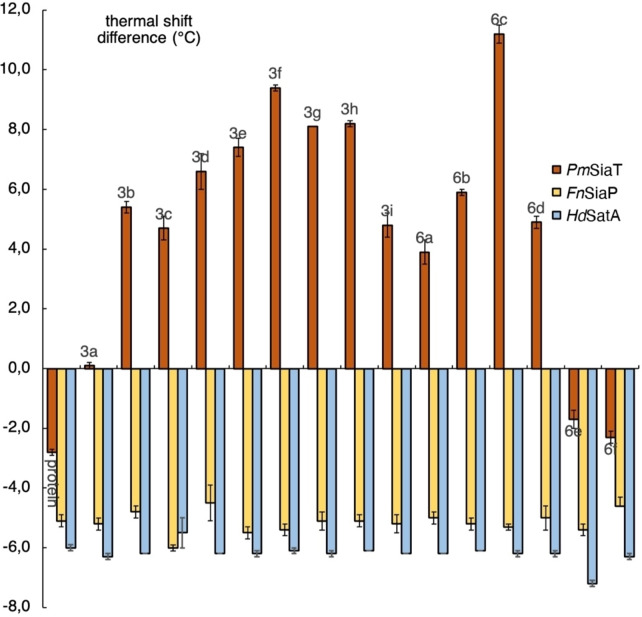
nanoDSF results of compounds **3 a‐i** and **6 a‐f**. The Δ*T*_m_ reported are given in °C and relative to Neu5Ac. Therefore, compounds with a positive Δ*T*_m_ indicate a thermal stabilization greater than Neu5Ac, while negative Δ*T*_m_ are associated with a smaller one. Compounds displaying Δ*T*_m_ values in the range of the control experiments (protein) are to be considered non‐binders.

Compounds **3 a‐e** bearing different cyclic amino containing moieties were initially synthesised to identify a suitable scaffold for further derivatisations (Scheme 1). The reactions proceeded smoothly, and the planned compounds **2 a‐e** were synthesised with yields ranging between 31–95 %. Ester hydrolyses (LiOH) afforded compounds **3 a‐e** in moderate to excellent yields. Evaluation of **3 a**‐**e** in a thermal shift assay (differential scanning fluorimetry, nanoDSF) as previously described,^[7]^ revealed the phenylpiperidine **3 e** to best stabilise *Pm*SiaT with a thermal shift (Δ*T*_m_) of 7.4 °C (relative to Neu5Ac, Figure 2). Encouraged by these results, we designed and synthesised a second generation of piperidine derivatives, **3 f**‐**i**, by the introduction of phenyl moieties with electron‐donating or ‐withdrawing substituents in meta or para position.

**Scheme 2:**
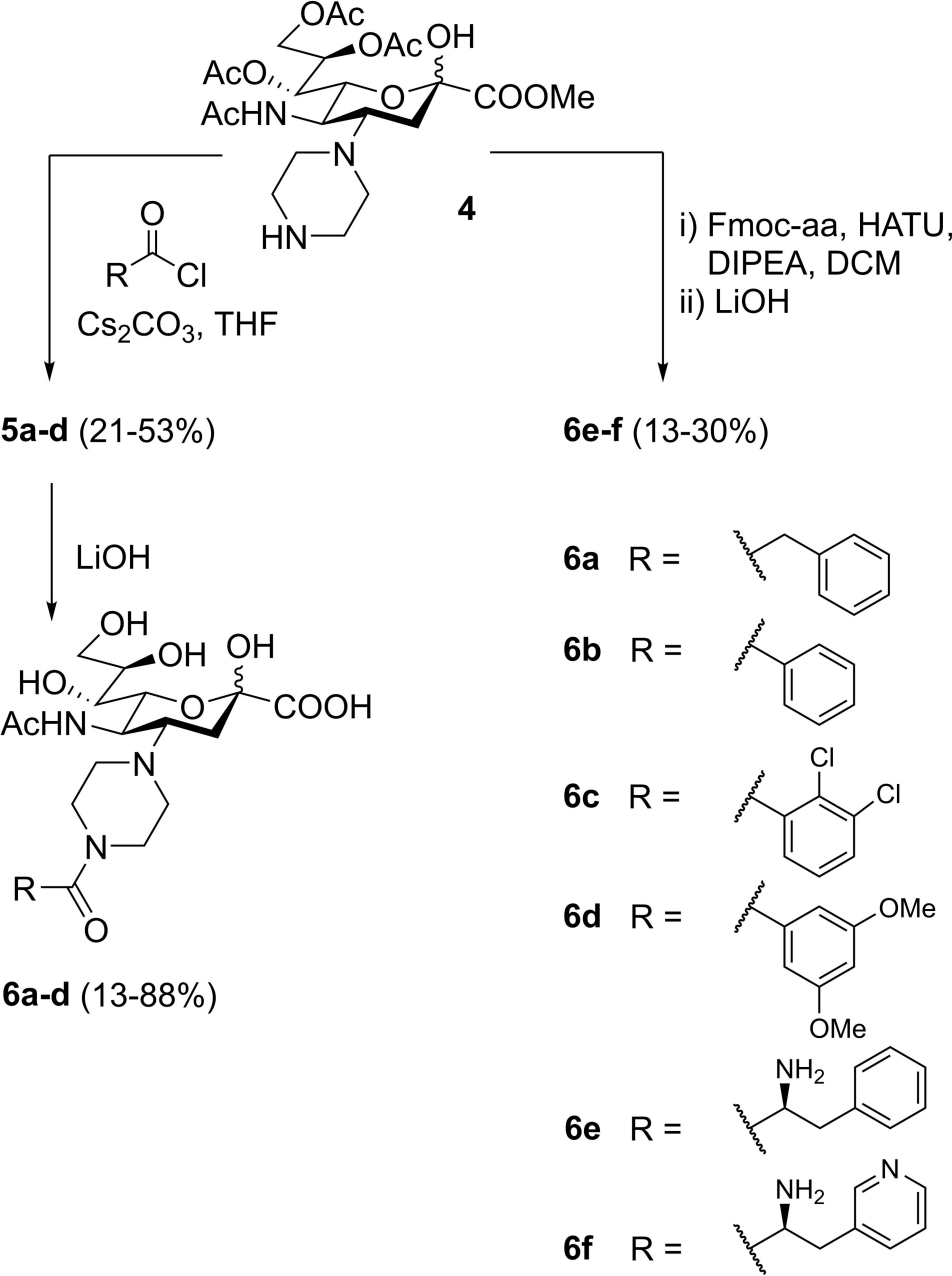
Synthesis of compounds **6 a‐f**

Furthermore, since the piperazine scaffold seemed to be tolerated by the protein, we decided to evaluate amides, which were synthesised by acylation of the distal amine of **4** (Scheme 2). The piperazine and the aromatic moieties were further separated by spacers of different lengths (**6 a‐b**) upon acylation of **4**.^[27]^ Amino acids (**6 e‐f**) were introduced to both increase the linker length and to probe for additional interactions. Finally, electron‐poor and electron‐rich derivatives (*i. e*., **6 c** and **d**) were synthesised.

The affinities of compounds **3 a**‐**i** and **6 a**‐**f** for *Pm*SiaT, *Fn*SiaP, and *Hd*SatA were evaluated with nanoDSF (Figure 2). *Fn*SiaP and *Hd*SatA showed no thermal stabilisations in the presence of any synthesised compound, indicating no binding. In contrast, evaluation of **3 a‐i**, **6 a‐f** with *Pm*SiaT revealed excellent thermal stabilizations, both for the piperidine and the piperazine series. Introduction of electron‐withdrawing substituents on the phenyl ring leads to higher thermal shifts than the underivatised structure (*e. g*., **3 f** >**3e** and **6 c** >**6b**). On the contrary, introduction of electron‐donating groups, as in the case of compounds **3 i** and **6 d**, causes reduction in thermal stabilisations. The amino acid portions of **6 e** and **f** led to complete loss of affinity, possibly for the excessive length of the linker or for detrimental interactions caused by the additional amino group.

To further evaluate the affinities of the compounds and their binding thermodynamics for *Pm*SiaT, we performed isothermal titration calorimetry (ITC) experiments. Compounds **3 f**, **3 i** and **6 c** were selected to probe both the compounds with the highest thermal stabilisations from nanoDSF (*i. e*., **3 f** and **6 c**), and one with a reduced one (**3 i**). The results are presented in Table 1. The nanoDSF screen indicates excellent correlation with the ITC data, showing the robustness of the results in orthogonal assays. For example, compound **6 c**, the one displaying the highest Δ*T*_m_, bind to *Pm*SiaT with a K_d_ value in the mid nanomolar range, with over 100‐fold affinity increase over Neu5Ac. From a thermodynamic perspective, the affinity increase seems to be driven by an increase in the entropic component. We have previously observed a similar trend and we believe that the large entropic gain is indicative of an entropy‐enthalpy transduction (EET).[^7^, ^28^]

**Table 1:** ITC data for Neu5Ac and compounds **3 i**, **3 f** and **6 c** with dissociation constants and binding thermodynamics listed. The confidence intervals for all values are found in the Supporting Information (Table S4).

	***Pm*** **SiaT**			
**Compound**	***K***_**d**_ **[μM]**	**Δ*G°* [kJ/mol]**	**Δ*H°* [kJ/mol]**	**−*T*Δ*S°* [kJ/mol]**
**Neu5Ac**	50^18^	−24.5	28.1	−52.6
**3 i**	17.9	−27.1	45.4	−72.5
**3 f**	1.32	−33.5	36.7	−70.2
**6 c**	0.47	−36.1	32.3	−68.4

**Figure 3:**
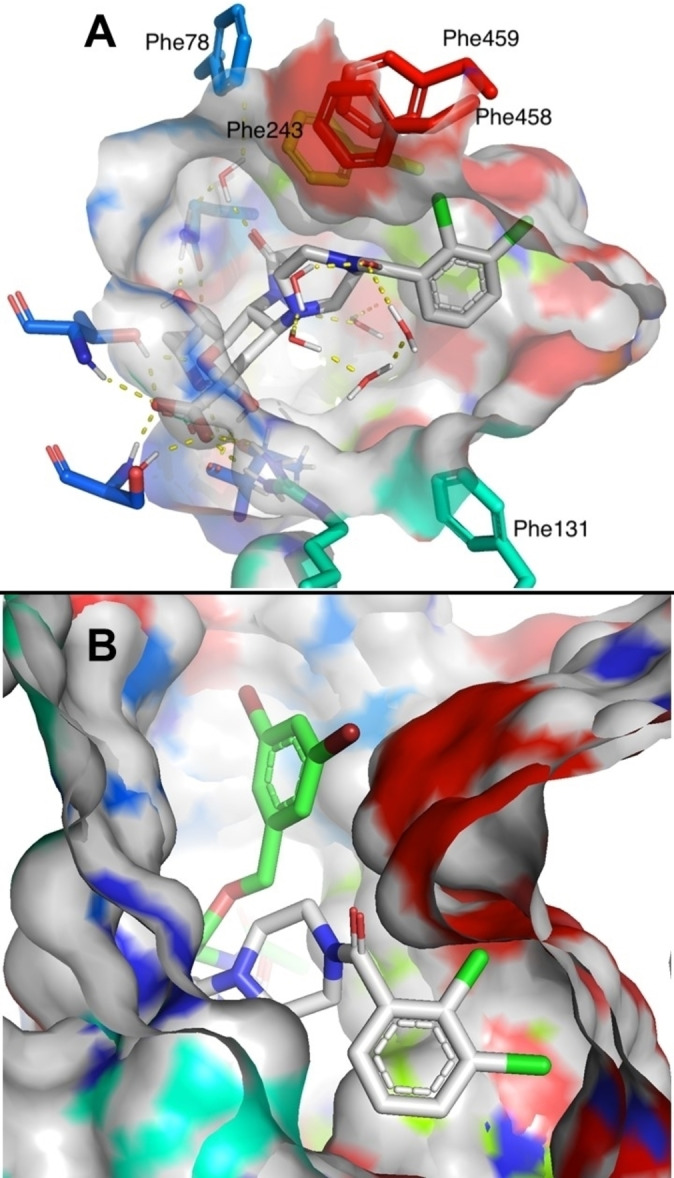
A) Representative MD snapshot at 90 ns from a 100 ns molecular dynamic simulation of **6 c** in complex with *Pm*SiaT (OPLS4 force field in Desmond, Schrödinger Release 2022‐1). The complex for the simulations was constructed by placing **6 c** with its Neu5Ac part identically positioned as Neu5Ac in the x‐ray structure of Neu5Ac in complex with the outward open *Pm*SiaT (pdb id 5NV9). The molecular dynamic simulation was performed as described.^[7]^ B) Overlay of the MD simulation 90 ns snapshots of compound **6 c** and the previously published 3,5‐dibromobenyl ether.^[7]^ Colour is according to atom type with protein carbon sticks coloured by helix. Protein surface carbons and **6 c** carbons are coloured light grey. The 3,5‐dibromobenzyl ether carbons are coloured green. From the overlay it is possible to appreciate the different portions of the binding site targeted by the two compounds. Molecular dynamics simulations were performed with OPLS4 implemented in Desmond (Schrödinger Release 2022‐1).

Preliminary computational analysis suggest that the compounds protrude their aryl‐substituted piperidine/piperazine moieties into the hydrophobic gate. However, the substituted piperidine/piperazine moieties do not fit ideally into the hydrophobic gate, but instead alter the flexible phenylalanine side chains in the hydrophobic gate to an induced fit, according to molecular dynamics simulations (Figure 3a). Hence, experimental structural analysis is necessary to reach conclusions about ligand binding modes. Nonetheless, the simulation indicates that compound **6 c** protrudes towards a different potion of the *Pm*SiaT binding site, when compared to the previously reported 3,5‐dibromobenzyl ether (Figure 3b).

## Conclusion

The introduction of aromatic moieties at O4 of Neu5Ac has been reported as a fruitful strategy to develop bacterial sialic acid uptake inhibitors. Here we explored new structural motives at C4, with piperidine and piperazine functionalities as handles to introduce and distance different aromatic moieties. The compound evaluation with a thermal shift assay (nanoDSF) revealed significant thermal stabilisations for *Pm*SiaT but showed no affinity for *Hd*SatA and *Fn*SiaP. *Fn*SiaP and *Hd*SatA are the SBPs of the TRAP and ABC transporters, respectively. Their binding event is described as the Venus flytrap or Pac‐Man models, in which the SBP binds the substrate with low nanomolar affinity.[^18^, ^29^, ^30^] The nanomolar affinity translates in a very tight binding site, and subsequently, only minor derivatisations seem to be allowed. By thermal shift assays of compounds **3 a**‐**i** and **6 a**‐**f** we show that the introduction of large substituents at C4 of Neu5Ac is not tolerated, neither by *Fn*SiaP nor by *Hd*SatA. Our investigations thus indicate that it may be difficult to design broad spectrum bacterial sialic acid uptake inhibitors. Further investigations are therefore required to develop compounds capable of targeting and inhibiting the TRAP and ABC transporters, either via the SPBs or the whole transporter system.

For *Pm*SiaT, piperidine or piperazine‐linked halogenated aromatic residues showed the largest thermal stabilizations, which satisfactorily translated in high affinities, as measured with ITC. Electron donating substituents, such as 3‐hydroxy and 3,5‐dimetoxy (compounds **3 i** and **6 d**, respectively), led to reduced affinities, when compared to the parent compounds. We hypothesise that these affinity‐increasing and decreasing effects can be ascribed to entropic factors, due to favourable desolvation penalty of the halogenated compounds (compounds **3 e‐h**, **6 c**) over the ones substituted with hydroxy or methoxy groups (**3 i** and **6 d**). Computational analyses indicate that compound **6 c** establishes interactions with Phe243, Phe458 and Phe459, residues which were not predicted to interact with the previously published benzyl ether series. The ability of targeting different druggable sites allows for the design of further generations of compounds to achieve improved drug‐like properties and potencies. Furthermore, the compounds reported in this work, all retain key structural elements of the natural substrate, Neu5Ac, which minimises the risk of cross‐binding to other symporters, such as human glucose transporters. We thus believe that these observations will enable identification of strong sialic acid uptake inhibitors, thus paving the way for new antibacterial drugs.

## Experimental Section

### Chemical synthesis

**General information**: All moisture‐ and air‐sensitive reactions were carried out under an atmosphere of dry nitrogen using oven‐dried glassware. All solvents were dried using MBRAUN SPS‐800 Solvent purification system prior to use, unless otherwise stated. Purchased reagents were used without further purification. Thin‐layer chromatography was performed on precoated TLC glass plates with silica gel 60 F_254_ 0.25 mm (Merck). Spots were visualised with UV light or by charring with a 5 % sulfuric acid in Ethanol solution. Biotage Isolute phase separators were used for drying of combined organic layers. Preparative chromatography was performed on a Biotage Isolera One flash purification system using Biotage SNAP Sfär HD silica cartridges. Agilent Technologies 1260 Infinity II HPLC with Waters XSelect CSH Prep C18 5.0 μM OBD, 19 mm×250 mm and water/acetonitrile mobile phase, was used for purification. The HPLC water phase contained either 0.1 % of formic acid (FA) or trifluoroacetic acid (TFA), thus leading to the isolation of the formate or trifluoroacetate salts for the final products. Optical rotations were measured on a Perkin Elmer instruments, Model 341 polarimeter. NMR spectra were recorded on a Bruker Avance II at 400 MHz (^1^H) and 100 MHz (^13^C) or on a Bruker Avance DMX‐500 (500 MHz) spectrometer. Assignment of ^1^H and ^13^C NMR spectra was achieved using 2D methods (COSY, HSQC, HMBC). Chemical shifts are expressed in ppm using residual solvent signals (CHCl_3_, CHD_2_OD, HDO) as reference. Coupling constant values are given in Hz. ^13^C‐NMR spectra are proton decoupled. Mass spectra were recorded on Waters XEVO G2 (positive ESI).

**General procedure A**: Starting material (1 eq) was dissolved in dry pyridine and the cyclic amine (5–10 eq) added. The reaction was stirred at room temperature and under a flow of nitrogen. Upon full conversion or formation of side products, the reaction mixture was concentrated, the residue dissolved in EtOAc and washed with water and brine. The organic phase was then dried, filtered, concentrated and purified by flash chromatography (heptane:EtOAc 70 : 30→0 : 100 in 8 column volumes (CV), then isocratic).

**General procedure B**: To the starting material (1 eq) solution in H_2_O‐ACN, LiOH (5–6 eq) was added. Upon full conversion, the solution was filtered and purified by prep‐HPLC, as described in the general information.

**Methyl 5‐acetamido‐4‐(morpholin‐4‐yl)‐7,8,9‐tri‐O‐acetyl‐3,4,5‐trideoxy‐D‐glycero‐β‐D‐galacto‐non‐2‐ulopyranosidonate (2 a)** As reference.^[27]^

**Methyl 5‐acetamido‐4‐(4‐phenylpiperazin‐1‐yl)‐7,8,9‐tri‐O‐acetyl‐3,4,5‐trideoxy‐D‐glycero‐β‐D‐galacto‐non‐2‐ulopyranosidonate (2 b)** Starting material **1** (100 mg, 0.187 mmol) was reacted with 4‐phenylpiperazine (0.295 mL, 1.870 mmol) as in general procedure A. The reaction was completed after 3 days and the product isolated as a white amorphous solid (96 mg, 86 %). [α]^20^_D_ +32.2 (c 1, CH_2_Cl_2_). ^1^H NMR (400 MHz, CDCl_3_) δ 7.33–7.19 (m, 2H, Ar−H), 7.03–6.71 (m, 3H, Ar−H), 5.36 (dd, *J*=6.1, 2.1 Hz, 1H, H‐7), 5.23 (ddd, *J*=7.3, 6.1, 2.4 Hz, 1H, H‐8), 5.08 (d, *J*=10.0 Hz, 1H, NH), 4.45 (dd, *J*=12.3, 2.4 Hz, 1H, H‐9), 4.18 (q, *J*=10.3 Hz, 1H, H‐5), 4.09 (dd, *J*=10.3, 2.1 Hz, 1H, H‐6), 4.02 (dd, *J*=12.3, 7.3 Hz, 1H, H‐9), 3.87 (s, 3H, COOCH_3_), 3.23–2.99 (m, 5H, N(CH_2_CH_2_)N ×4, H‐4), 2.91 ‐ 2.83 (m, 2H, N(CH_2_CH_2_)N ×2), 2.54–2.47 (m, 2H, N(CH_2_CH_2_)N ×2), 2.14 (s, 3H, OCOCH_3_), 2.11–1.98 (8H, OCOCH_3_ x2, H‐3eq, H‐3ax), 1.93 (s, 3H, HNCOCH_3_). ^13^C NMR (101 MHz, CDCl_3_) δ 170.9, 170.7, 170.5, 170.5, 170.0, 169.2, 151.5, 129.4, 120.7, 116.8, 95.3, 72.5, 71.1, 68.6, 63.0, 61.6, 53.6, 49.9, 49.5, 48.2, 46.8, 29.5, 23.4, 21.5, 21.1, 21.0, 20.9. ESI‐MS (m/z): Calcd. for C_28_H_39_N_3_O_11_+ H^+^ [M+H]+, 594.3; found, 594.3.

**Methyl 5‐acetamido‐4‐[4‐(2‐pyridyl)piperazin‐1‐yl]‐7,8,9‐tri‐O‐acetyl‐3,4,5‐trideoxy‐D‐glycero‐β‐D‐galacto‐non‐2‐ulopyranosidonate (2 c)** Starting material **1** (100 mg, 0.187 mmol) was reacted with 1‐(2‐pyridyl)piperazine (0.285 mL, 1.870 mmol) as in general procedure A. The reaction was completed after 4 days and the product isolated as a white amorphous solid (67 mg, 60 %). [α]^20^_D_ +30.8 (c 1, CH_2_Cl_2_). ^1^H NMR (400 MHz, CDCl_3_) δ 8.18–8.13 (m, 1H, Ar−H), 7.48–7.41 (m, 1H, Ar−H), 6.62–6.56 (m, 2H, Ar−H), 5.36 (dd, *J*=6.2, 2.2 Hz, 1H), 5.27–5.19 (m, 1H, H‐8), 5.07 (d, *J*=9.9 Hz, 1H, NH), 4.44 (dd, *J*=12.3, 2.4 Hz, 1H, H‐9), 4.18 (q, *J*=10.2 Hz, 1H, H‐5), 4.09 (dd, *J*=10.2, 2.2 Hz, 1H, H‐6), 4.02 (dd, *J*=12.3, 7.3 Hz, 1H, H‐9), 3.86 (s, 3H, COOCH_3_), 3.49–3.44 (m, 1H, N(CH_2_CH_2_)N ×2), 3.43–3.34 (m, 1H, N(CH_2_CH_2_)N ×2), 3.08–2.99 (m, 2H, N(CH_2_CH_2_)N ×2), 2.86–2.78 (m, 2H, N(CH_2_CH_2_)N ×2), 2.51–2.45 (m, 2H, N(CH_2_CH_2_)N ×2), 2.15 (s, 3H, OCOCH_3_), 2.10‐2.01 (s, 7H, OCOCH_3_ ×2, H‐3eq), 1.98 (dd, *J*=12.9, 4.2 Hz, 1H, H‐3ax), 1.94 (s, 3H, HNCOCH_3_). ^13^C NMR (101 MHz, CDCl_3_) δ 170.9, 170.5, 170.0, 169.3, 148.2, 137.8, 114.1, 107.4, 95.3, 72.4, 71.0, 68.6, 63.0, 61.7, 53.6, 48.2, 46.8, 46.1, 45.2, 41.2, 29.6, 23.4, 21.6, 21.1, 21.1, 21.0. ESI‐MS (m/z): Calcd. for C_27_H_38_N_4_O_11_+H^+^ [M+H]+, 595.3; found, 595.3.

**Methyl 5‐acetamido‐4‐[4‐(2‐pyrimidyl)piperazin‐1‐yl]‐7,8,9‐tri‐O‐acetyl‐3,4,5‐trideoxy‐D‐glycero‐β‐D‐galacto‐non‐2‐ulopyranosidonate (2 d)** Starting material **1** (100 mg, 0.187 mmol) was reacted with 1‐(2‐pyrimidyl)piperazine (0.265 mL, 1.870 mmol) as in general procedure A. The reaction was completed after 4 days and the product isolated as a white amorphous solid (65 mg, 58 %). [α]^20^_D_ +43.6 (c 1, CH_2_Cl_2_). ^1^H NMR (400 MHz, CDCl_3_) δ 8.27 (d, *J*=4.7 Hz, 2H, Ar−H), 6.45 (t, *J*=4.7 Hz, 1H, Ar−H), 5.36 (dd, *J*=6.1, 2.1 Hz, 1H, H‐7), 5.27–5.18 (m, 1H, H‐8), 5.13 (d, *J*=9.6 Hz, 1H, NH), 4.45 (dd, *J*=12.3, 2.4 Hz, 1H, H‐9), 4.23–3.97 (m, 2H, H‐5, H‐6), ), 4.01 (dd, *J*=12.3, 7.3 Hz, 1H, H‐9), 3.85 (s, 3H, COOCH_3_), 3.79–3.57 (m, 4H, N(CH_2_CH_2_)N ×2), 3.03 (td, *J*=11.5, 3.9 Hz, 1H, H‐4), 2.80–2.72 (m, 2H, N(CH_2_CH_2_)N ×2), 2.47–2.37 (m, 2H, N(CH_2_CH_2_)N ×2), 2.15 (s, 3H, OCOCH_3_), 2.09 (s, 3H, OCOCH_3_), 2.07–1.92 (m, 8H, H‐3eq, H‐3ax, OCOCH_3_, HNCOCH_3_). ^13^C NMR (101 MHz, CDCl_3_) δ 170.9, 170.6, 170.6, 170.5, 170.0, 161.9, 157.8, 109.9, 95.3, 72.5, 71.1, 68.6, 63.0, 61.8, 53.6, 48.3, 46.8, 44.5, 29.7, 23.4, 21.2, 21.1, 21.0. Calcd. for C_26_H_37_N5O_11_+H^+^ [M+H]+, 596.3; found, 596.3.

**Methyl 5‐acetamido‐4‐(4‐phenylpiperidin‐1‐yl)‐7,8,9‐tri‐O‐acetyl‐3,4,5‐trideoxy‐D‐glycero‐β‐D‐galacto‐non‐2‐ulopyranosidonate (2 e)** To the starting material **1** (100 mg, 0.187 mmol) solution in dry pyridine (2 mL), 4‐phenylpiperidine (302 mg, 1.870 mmol) was added according to general procedure A. The product was obtained 118 mg (>95 %). [α]^20^_D_ +8.3 (c 1, CH_2_Cl_2_). ^1^H NMR (400 MHz, CDCl_3_) δ 7.35–7.14 (m, 5H, Ar−H), 6.57 (d, *J*=10.0 Hz, 1H, AcNH), 5.38 (dd, *J*=6.2, 2.1 Hz, 1H, H‐7), 5.28 (ddd, *J*=7.2, 6.2, 2.4 Hz, 1H, H‐8), 4.44 (dd, *J*=12.3, 2.4 Hz, 1H, H‐9), 4.27 (q, *J*=10.3 Hz, 1H, H‐5), 4.13 (dd, *J*=10.0, 2.1 Hz, 1H, H‐6), 4.01 (dd, *J*=12.3, 7.3 Hz, 1H, H‐9), 3.87 (s, 3H, COOCH_3_), 3.49 (td, *J*=11.0, 4.9 Hz, 1H, H‐4), 3.29 (d, *J*=11.2 Hz, 1H, piperidine), 2.93 (d, *J*=11.3 Hz, 1H, piperidine), 2.75 (t, *J*=11.4 Hz, 1H, piperidine), 2.57–2.38 (m, 2H, piperidine x2), 2.21–1.69 (m, 18H, H‐3_eq_, H‐3_ax_, OCOCH_3_ ×3, HNCOCH_3_, piperidine x4). ^13^C NMR (101 MHz, CDCl_3_) δ 171.1, 170.9, 170.7, 170.4, 169.7, 145.1, 128.7, 126.9, 126.6, 95.1, 72.5, 70.9, 68.4, 63.0, 61.4, 53.6, 52.1, 46.7, 46.5, 42.1, 32.9, 32.2, 29.8, 23.3, 21.2, 21.1, 20.9. ESI‐MS (m/z): Calcd. for C_29_H_40_N_2_O_11_+H^+^ [M+H]+, 593.3; found, 593.3.

**Methyl 5‐acetamido‐4‐[4‐(3‐bromophenyl)piperidin‐1‐yl]‐7,8,9‐tri‐O‐acetyl‐3,4,5‐trideoxy‐D‐glycero‐β‐D‐galacto‐non‐2‐ulopyranosidonate (2 f)** Starting material **1** (100 mg, 0.187 mmol) was reacted with 4‐(3‐bromophenyl)piperidine ( mg, 0.935 mmol) as in general procedure A. The reaction was completed after 4 days and the product isolated as a white amorphous solid (39 mg, 31 %). [α]^20^_D_ +17.0 (c 1, CH_2_Cl_2_). ^1^H NMR (400 MHz, CDCl_3_) δ 7.38–7.28 (m, 2H, Ar−H), 7.13 (dt, *J*=15.5, 7.8 Hz, 2H, Ar−H), 5.34 (dd, *J*=6.4, 2.1 Hz, 1H, H‐7), 5.24 (td, *J*=6.9, 2.4 Hz, 1H, H‐8), 4.44 (dd, *J*=12.3, 2.4 Hz, 1H, H‐9), 4.18 (q, *J*=10.3 Hz, 1H, H‐5), 4.07 (dd, *J*=10.3, 2.1 Hz, 1H, H‐6), 4.01 (dd, *J*=12.3, 7.3 Hz, 1H, H‐9), 3.88 (s, 3H, COOCH_3_), 3.14–3.02 (m, 2H, H‐4, piperidine), 2.76 (m, 1H, piperidine), 2.62 (m, 1H, piperidine), 2.39 (m, 1H, piperidine), 2.24 (m, 1H, piperidine), 2.17–1.94 (m, 14H, OCOCH_3_ ×3, HNCOCH_3,_ H‐3_eq_, H‐3_ax_), 1.87–1.66 (m, 4H, piperidine ×4). ^13^C NMR (101 MHz, CDCl_3_) δ 170.9, 170.7, 170.6, 170.5, 170.0, 148.5, 130.1, 129.4, 125.6, 122.6, 95.3, 72.5, 71.0, 68.5, 63.0, 61.8, 53.6, 52.1, 46.9, 46.4, 42.5, 34.2, 29.7, 23.5, 21.2, 21.1, 21.0. Calcd. for C_29_H_39_BrN_3_O_12_+H^+^ [M+H]+, 671.2; found, 671.2.

**Methyl 5‐acetamido‐4‐[4‐(4‐bromophenyl)piperidin‐1‐yl]‐7,8,9‐tri‐O‐acetyl‐3,4,5‐trideoxy‐D‐glycero‐β‐D‐galacto‐non‐2‐ulopyranosidonate (2 g)** Starting material **1** (100 mg, 0.187 mmol) was reacted with 4‐(4‐bromophenyl)piperidine (225 mg, 0.935 mmol) as in general procedure A. The reaction was completed after 4 days and the product isolated as a white amorphous solid (64 mg, 51 %). [α]^20^_D_ +15.0 (c 1, CH_2_Cl_2_). ^1^H NMR (400 MHz, CDCl_3_) δ 7.39 (d, *J*=8.5 Hz, 2H, Ar−H), 7.04 (d, *J*=8.5 Hz, 2H, Ar−H), 5.34 (dd, *J*=6.2, 2.1 Hz, 1H, H‐7), 5.27–5.19 (m, 1H, H‐8), 5.06 (d, *J*=10.0 Hz, 1H, AcNH), 4.46 (dd, *J*=12.3, 2.4 Hz, 1H, H‐9), 4.20–4.04 (m, 2H, H‐5, H‐6), 4.01 (dd, *J*=12.3, 7.4 Hz, 1H, H‐9), 3.87 (s, 3H, COOCH_3_), 2.99 (m, 2H, H‐4, piperidine), 2.74‐2.68 (m, 1H, piperidine), 2.61‐ 2.53 (m, 1H, piperidine), 2.42–2.32 (m, 1H, piperidine), 2.21–1.93 (m, 14H, OCOCH_3_ ×3, HNCOCH_3,_ H‐3_eq_, H‐3_ax_), 1.86–1.39 (m, 5H, piperidine). ^13^C NMR (101 MHz, CDCl_3_) δ 170.9, 170.6, 170.6, 170.1, 131.5, 128.7, 95.4, 72.6, 71.1, 68.6, 63.0, 61.9, 53.6, 52.1, 47.0, 46.5, 42.4, 29.8, 23.5, 21.2, 21.1, 21.0. Calcd. for C_29_H_39_BrN_3_O_12_+H^+^ [M+H]+, 671.2; found, 671.2.

**Methyl 5‐acetamido‐7,8,9‐tri‐O‐acetyl‐3,4,5‐trideoxy‐4‐[4‐(3‐trifluoromethylphenyl)piperidin‐1‐yl]‐D‐glycero‐β‐D‐galacto‐non‐2‐ulopyranosidonate (2 h)** Starting material **1** (100 mg, 0.187 mmol) was reacted with 4‐(3‐trifluoromethylphenyl)piperidine (214 mg, 0.935 mmol) as in general procedure A. The reaction was completed after 4 days and the product isolated as a white amorphous solid (62 mg, 50 %). [α]^20^_D_ +3.0 (c 1, CH_2_Cl_2_). ^1^H NMR (500 MHz, CDCl_3_) δ 7.60–7.34 (m, 4H, Ar−H), 5.36 (dd, *J*=6.9, 2.1 Hz, 1H, H‐7), 5.28 (m, 1H, H‐8), 4.45–4.21 (m, 2H, H‐9, H‐5), 4.15–4.09 (m, 1H, H‐6), 4.00 (dd, *J*=12.4, 7.0 Hz, 1H, H‐9), 3.89 (s, 3H, COOCH_3_), 3.03 (m, 1H, H‐4), 3.26–2.42 (m, 5H, piperidine), 2.27–1.81 (m, 14H, OCOCH_3_ ×3, HNCOCH_3,_ H‐3_eq_, H‐3_ax_). ^13^C NMR (126 MHz, CDCl_3_) δ 170.7, 170.4, 170.1, 130.0, 129.8, 129.0, 123.6, 94.8, 72.0, 69.7, 67.3, 62.6, 61.6, 53.5, 52.0, 46.3, 29.6, 21.0, 20.8, 20.7. ESI‐MS (m/z): Calcd. for C_30_H_39_F_3_N_2_O_11_+H^+^ [M+H]+, 661.2; found, 662.3.

**Methyl 5‐acetamido‐4‐[4‐(3‐hydroxyphenyl)piperidin‐1‐yl]‐7,8,9‐tri‐O‐acetyl‐3,4,5‐trideoxy‐D‐glycero‐β‐D‐galacto‐non‐2‐ulopyranosidonate (2 i)** Starting material **1** (100 mg, 0.187 mmol) was reacted with 4‐(3‐hydroxyphenyl)piperidine (166 mg, 0.935 mmol) as in general procedure A. The reaction was completed after 4 days and the product isolated as a white amorphous solid (47 mg, 41 %). [α]^20^_D_ +31.0 (c 1, CH_2_Cl_2_). ^1^H NMR (400 MHz, CDCl_3_) δ 7.12 (dd, *J*=8.8, 7.6 Hz, 1H, Ar−H), 6.73–6.59 (m, 3H, Ar−H), 5.35 (dd, *J*=6.0, 2.1 Hz, 1H; H‐7), 5.23 (ddd, *J*=7.5, 6.0, 2.4 Hz, 1H, H‐8), 4.47 (dd, *J*=12.4, 2.4 Hz, 1H, H‐9), 4.17 (q, *J*=10.3 Hz, 1H, H‐6), 4.08 (dd, *J*=10.3, 2.1 Hz, 1H, H‐6), 4.01 (dd, *J*=12.4, 7.5 Hz, 1H, H‐9), 3.87 (s, 3H, COOCH_3_), 3.07–2.93 (m, 2H, H‐4, piperidine), 2.71 (m, 1H, piperidine), 2.57 (m, 1H, piperidine), 2.35 (m, 1H, piperidine), 2.25–1.94 (m, 14H, OCOCH_3_ ×3, HNCOCH_3,_ H‐3_eq_, H‐3_ax_), 1.84–1.37 (m, 5H, piperidine). ^13^C NMR (101 MHz, CDCl_3_) δ 171.0, 170.9, 170.7, 170.6, 170.1, 156.2, 148.3, 129.6, 119.2, 113.8, 113.3, 95.4, 72.6, 71.2, 68.6, 63.0, 61.8, 53.6, 52.3, 47.1, 46.4, 42.7, 34.4, 33.4, 29.6, 23.5, 21.2, 21.1, 20.9. Calcd. for C_29_H_40_N_3_O_12_+H^+^ [M+H]+, 609.3; found, 609.3.

**Methyl 5‐acetamido‐4‐(4‐phenylacetylpiperazin‐1‐yl)‐7,8,9‐tri‐O‐acetyl‐3,4,5‐ trideoxy‐D‐glycero‐β‐D‐galacto‐non‐2‐ulopyranosidonate (5 a)** Starting material **4** (100 mg, 0.193 mmol) was dissolved in dry THF (2 mL), then caesium carbonate (157 mg, 0.482 mmol) and phenylacetyl chloride (0.051 mL, 0.386 mmol) were added. The reaction was completed after 2 hours. The reaction was concentrated, dissolved in EtOAc, washed with water and brine, dried and purified via flash chromatography. The product was isolated as a white amorphous solid (26 mg, 21 %). [α]^20^_D_ +24.4 (c 1, CH_2_Cl_2_). ^1^H NMR (500 MHz, CDCl_3_) δ 7.37–7.17 (m, 5H, Ar−H), 5.34 (dd, *J*=6.1, 1.7 Hz, 1H, H‐7), 5.21 (dq, *J*=7.3, 2.4 Hz, 1H, H‐8), 4.42 (dd, *J*=12.4, 2.4 Hz, 1H, H‐9), 4.12 (d, *J*=17.9 Hz, 2H, H‐5, H‐6), 3.99 (dd, *J*=12.4, 7.3 Hz, 1H, H‐9), 3.86 (s, 3H, COOCH_3_), 3.69 (s, 2H, Ph‐CH_z_‐), 3.61–2.53 (m, 2H, N(CH_2_CH_2_)N, 3.41 (m, 1H, H‐4), 3.33–3.14 (m, 2H, N(CH_2_CH_2_)N, 2.88–2.39 (m, 4H, N(CH_2_CH_2_)N), 2.16–1.86 (m, 14H„ H‐3_eq_, H‐3_ax_, HNCOCH_3,_ OCOCH_3_ ×3). ^13^C NMR (126 MHz, CDCl_3_) δ 174.6, 170.9, 170.7, 170.3, 169.7, 169.5, 129.5, 129.0, 128.7, 127.3, 95.0, 72.0, 70.9, 68.3, 62.8, 61.6, 53.6, 51.0, 46.3, 41.1, 41.1, 29.8, 29.6, 23.3, 21.1, 21.0, 20.9. Calcd. for C_30_H_40_N_3_O_12_+Na^+^ [M+Na]+, 658.2; found, 657.9.

**Methyl 5‐acetamido‐4‐(4‐benzoylpiperazin‐1‐yl)‐7,8,9‐tri‐O‐acetyl‐3,4,5‐ trideoxy‐D‐glycero‐β‐D‐galacto‐non‐2‐ulopyranosidonate (5 b)** Starting material **4** (100 mg, 0.193 mmol) was dissolved in dry THF (2 mL), then caesium carbonate (157 mg, 0.482 mmol) and benzoyl chloride (0.045 mL, 0.386 mmol) were added. The reaction was completed after 2 hours. The reaction was concentrated, dissolved in EtOAc, washed with water and brine, dried and purified via flash chromatography. The product was isolated as a white amorphous solid (51 mg, 43 %). [α]^20^_D_ +27.5 (c 0.4, MeOH). ^1^H NMR (500 MHz, CDCl_3_) δ 7.48–7.37 (m, 5H, Ar−H), 5.33 (dd, *J*=6.9, 1.8 Hz, 1H, H‐7), 5.28 (td, *J*=6.9, 2.5 Hz, 1H, H‐8), 4.35 (m, *J*=12.3, 2.5 Hz, 2H, H‐6, H‐9), 4.20 (d, *J*=9.6 Hz, 1H, H‐5), 4.07 (d, *J*=8.7 Hz, 1H, H‐4), 3.98 (dd, *J*=12.3, 6.9 Hz, 1H, H‐9), 3.89 (s, 3H, COOCH_3_), 3.84–3.34 (m, 8H, N(CH_2_CH_2_)N ×2), 2.20–1.92 (m, 14H, H‐3_eq_, H‐3_ax_, HNCOCH_3,_ OCOCH_3_ ×3). ^13^C NMR (126 MHz, CDCl_3_) δ 172.51, 170.92, 170.88, 170.60, 170.07, 169.00, 168.32, 133.60, 131.06, 128.82, 127.24, 94.38, 71.67, 70.09, 67.54, 62.51, 61.83, 53.88, 45.89, 29.86, 25.19, 23.38, 21.50, 21.17, 20.95, 20.91. Calcd. for C_29_H_39_N_3_O_12_+Na^+^ [M+Na]+, 644.2; found, 644.0.

**Methyl 5‐acetamido‐4‐[4‐(2,3‐dichlorobenzoyl)piperazin‐1‐yl]‐7,8,9‐tri‐O‐acetyl‐3,4,5‐ trideoxy‐D‐glycero‐β‐D‐galacto‐non‐2‐ulopyranosidonate (5 c)** Starting material **4** (100 mg, 0.193 mmol) was dissolved in dry THF (2 mL), then caesium carbonate (157 mg, 0.482 mmol) and 2,3‐dichlorobenzoyl chloride (61 mg, 0.289 mmol) were added. The reaction was completed after 1 hour. The reaction was concentrated, dissolved in EtOAc, washed with water and brine, dried and purified via flash chromatography. The product was isolated as a white amorphous solid (60 mg, 53 %). [α]^20^_D_ +13.8 (c 0.6, MeOH). ^1^H NMR (400 MHz, MeOD) δ 7.46–7.24 (m, 3H, Ar−H), 5.42 (dd, *J*=4.8, 2.4 Hz, 1H, H‐7), 5.16 (ddd, *J*=7.4, 4.8, 2.5 Hz, 1H, H‐8), 4.56 (dd, 1H, *J*=2.5, 12.3, H‐9), 4.21 (d, *J*=10.1 Hz, 1H, H‐6), 4.07 (dd, *J*=12.3, 7.4 Hz, 1H, H‐9), 3.98 (t, *J*=10.6 Hz, 1H, H‐5), 3.78 (s, 3H, OCOCH_3_), 3.73–3.51 (m, 4H, N(CH_2_CH_2_)N ×4), 3.35 (s, 3H, COOCH_3_), 3.19–3.03 (m, 1H, H‐4), 2.90‐2.81 (m, 1H, N(CH_2_CH_2_)N ×1), 2.80‐2.67 (m, 2H, N(CH_2_CH_2_)N ×2), 2.56–2.48 (m, 1H, N(CH_2_CH_2_)N ×1), 2.16 (s, 3H, OCOCH_3_), 2.01 (m, 7H, H‐3_eq_, OCOCH_3_ ×2), 1.89 (s, 4H, H‐3_ax_, HNCOCH_3_). ^13^C NMR (126 MHz, MeOD) δ 173.4, 172.5, 172.1, 171.4, 168.3, 167.9, 133.0, 132.5, 129.9, 128.8, 127.4, 127.4, 96.4, 73.0, 72.9, 70.4, 63.8, 62.2, 53.2, 47.5, 46.8, 43.7, 42.9, 42.7, 42.6, 42.1, 31.9, 22.7, 21.2, 20.9, 20.6. Calcd. for C_29_H_37_Cl_2_N_3_O_12_+H^+^ [M+H]+, 690.0; found, 689.8.

**Methyl 5‐acetamido‐4‐[4‐(3,5‐dimethoxybenzoyl)piperazin‐1‐yl]‐7,8,9‐tri‐O‐acetyl‐3,4,5‐ trideoxy‐D‐glycero‐β‐D‐galacto‐non‐2‐ulopyranosidonate (5 d)** Starting material **4** (100 mg, 0.193 mmol) was dissolved in dry THF (2 mL), then caesium carbonate (157 mg, 0.482 mmol) and 3,5‐dimethoxybenzoyl chloride (49 mg, 0.289 mmol) were added. The reaction was completed after 1 hour. The reaction was concentrated, dissolved in EtOAc, washed with water and brine, dried and purified via flash chromatography. The product was isolated as a white amorphous solid (59 mg, 45 %). [α]^20^_D_ +13.9 (c 0.7, MeOH).^1^H NMR (500 MHz, MeOD) δ 6.59‐6.49 (m, 3H, Ar−H), 5.43 (dd, *J*=4.7, 2.4 Hz, 1H, H‐7), 5.16 (ddd, *J*=7.3, 4.7, 2.6 Hz, 1H, H‐8), 4.57 (dd, *J*=12.3, 2.6 Hz, 1H, H‐9), 4.22 (dd, *J*=10.3, 2.4 Hz, 1H, H‐6), 4.07 (dd, *J*=12.3, 7.4 Hz, 1H, H‐9), 3.98 (t, *J*=10.7 Hz, 1H, H‐5), 3.81 (s, 6H, Ar‐OCH_3_ ×2), 3.79 (s, 3H, COOCH_3_), 3.76–3.41 (m, 4H, N(CH_2_CH_2_)N), 3.06 (td, *J*=11.7, 3.8 Hz, 1H, H‐4), 2.86–2.78 (m, 1H, N(CH_2_CH_2_)N), 2.75–2.68 (m, 1H, N(CH_2_CH_2_)N), 2.52–2.45 (m, 1H, N(CH_2_CH_2_)N), 2.37–2.31 (m, 1H, N(CH_2_CH_2_)N), 2.08 (s, 3H, OCOCH_3_), 2.05–1.97 (m, 7H, H‐3_eq_, OCOCH_3_ ×2), 1.90 (s, 3H, HNCOCH_3_), 1.85 (t, *J*=12.7 Hz, 1H, H‐3_ax_). ^13^C NMR (126 MHz, MeOD) δ 173.4, 172.5, 172.1, 172.0, 171.9, 171.4, 162.7, 162.6, 108.3, 105.8, 105.7, 102.7, 96.5, 73.0, 70.5, 63.8, 62.2, 56.0, 56.0, 55.9, 53.2, 47.7, 31.8, 22.7, 21.2, 20.9, 20.8, 20.6. Calcd. for C_31_H_43_N_3_O_14_+H^+^ [M+H]+, 682.3; found, 681.9.

**5‐Acetamido‐4‐(morpholin‐4‐yl)‐3,4,5‐trideoxy‐D‐glycero‐β‐D‐galacto‐nonulosonic acid (3 a) FA salt** Compound **2 a** (40 mg, 0.0771 mmol) was treated with LiOH (11 mg, 0.463 mmol) as in general procedure B. Purification afforded 24 mg (82 %) of compound **3 a**. [α]^20^_D_ +5.0 (c 1, MeOH) ^1^H NMR (400 MHz, MeOD) δ 4.18 (t, *J*=10.2 Hz, 1H, H‐5), 4.03 (dd, *J*=10.2, 1.1 Hz, 1H, H‐6), 3.79 (dd, *J*=11.1, 2.7 Hz, 1H, H‐9), 3.70–3.55 (m, 6H, H‐8, H‐9, morpholine ×4), 3.45 (dd, *J*=9.0, 1.1 Hz, 1H, H‐7), 3.10 (td, *J*=11.6, 3.9 Hz, 1H, H‐4), 2.80–2.71 (m, 2H, morpholine ×2), 2.50–2.40 (m, 2H, morpholine ×2), 2.00–1.91 (m, 4H, H‐3eq, NHCOCH_3_), 1.84 (t, *J*=12.4 Hz, 1H, H‐3ax). ^13^C NMR (101 MHz, MeOD) δ 177.8, 173.8, 97.5, 72.8, 72.1, 70.9, 68.7, 65.1, 62.2, 48.8, 47.9, 32.0, 22.8. ESI‐HRMS (m/z): calcd. for C_15_H_26_N_2_O_9_+H^+^ [M+H]+, 379.1721; found, 379.1717.

**5‐Acetamido‐4‐(4‐phenylpiperazin‐1‐yl)‐3,4,5‐trideoxy‐D‐glycero‐β‐D‐galacto‐nonulosonic acid (3 b) FA salt** Compound **2 b** (40 mg, 0.0674 mmol) was treated with LiOH (10 mg, 0.404 mmol) as in general procedure B. Purification afforded 17 mg (56 %) of compound **3 b**. [α]^20^_D_ +32.2. (c 1, H_2_O). ^1^H NMR (400 MHz, MeOD) δ 7.27–7.17 (m, 2H, Ar−H), 6.99–6.92 (m, 2H, Ar−H), 6.83 (tt, *J*=7.2, 1.0 Hz, 1H, Ar−Hz), 4.32 (t, *J*=10.0 Hz, 1H, H‐5), 4.10 (d, *J*=10.0 Hz, 1H, H‐6), 3.80 (dd, *J*=11.1, 2.8 Hz, 1H, H‐9), 3.68 (ddd, *J*=8.8, 5.8, 2.8 Hz, 1H, H‐8), 3.61 (dd, *J*=11.1, 5.8 Hz, 1H, H‐9), 3.47 (d, *J*=8.8 Hz, 1H, H‐7), 3.31 (m, 1H under MeOD peak, H‐4), 3.25–3.06 (m, 6H, piperidine ×6), 2.80 (s, 2H, piperidine ×2), 2.09–1.92 (m, 5H, H‐3eq, H‐3ax, NHCOCH_3_). ^13^C NMR (101 MHz, D_2_O) δ 176.1, 172.4, 150.5, 131.1, 123.8, 119.0, 97.0, 72.0, 71.6, 69.8, 64.6, 47.1, 32.1, 23.7. ESI‐HRMS (m/z): calcd. for C_21_H_31_N_3_O_8_+H^+^ [M+H]+, 454.2186; found, 454.2189.

**5‐Acetamido‐4‐[4‐(2‐pyridyl)piperazin‐1‐yl]‐3,4,5‐trideoxy‐D‐glycero‐β‐D‐galacto‐nonulosonic acid (3 c) FA salt** Compound **2 c** (40 mg, 0.0673 mmol) was treated with LiOH (9 mg, 0.404 mmol) as in general procedure B. Purification afforded 27 mg (90 %) of compound **3 c**. [α]^20^_D_ +20.0 (c 0.2, H_2_O).^1^H NMR (400 MHz, MeOD) δ 8.05 (ddd, *J*=5.1, 2.0, 0.8 Hz, 1H, Ar−H), 7.53 (ddd, *J*=8.9, 7.1, 2.0 Hz, 1H, Ar−H), 6.79 (d, *J*=8.7 Hz, 1H, Ar−H), 6.68–6.60 (m, 1H, Ar−H), 4.22 (t, *J*=10.7 Hz, 1H, H‐5), 4.06 (dd, *J*=10.2, 1.2 Hz, 1H, H‐6), 3.80 (dd, *J*=11.1, 2.7 Hz, 1H, H‐9), 3.69–3.64 (m, 1H, H‐8), 3.60 (dd, *J*=11.1, 6.0 Hz, 1H, H‐9), 3.49–3.39 (m, 5H, H‐7, piperazine ×4), 3.20 (td, *J*=11.6, 4.3 Hz, 1H, H‐4), 2.90–2.83 (m, 2H, piperazine ×2), 2.60–2.52 (m, 2H, piperazine ×2), 1.98–1.82 (m, 5H, H‐3eq, H‐3ax, NHCOCH_3_). ^13^C NMR (101 MHz, MeOD) δ 178.6, 170.1, 161.2, 148.3, 139.1, 114.4, 109.2, 97.5, 72.9, 72.1, 71.0, 65.2, 62.0, 48.8, 47.4, 32.2, 23.0, 22.8. ESI‐HRMS (m/z): calcd. for C_20_H_30_N_4_O_8_+H^+^ [M+H]+, 455.2142; found, 455.2141.

**5‐Acetamido‐4‐[4‐(2‐pyrimidyl)piperazin‐1‐yl]‐3,4,5‐trideoxy‐D‐glycero‐β‐D‐galacto‐nonulosonic acid (3 d) FA salt** Compound **2 d** (45 mg, 0.0756 mmol) was treated with LiOH (11 mg, 0.453 mmol) as in general procedure B. Purification afforded 28 mg (81 %) of compound **3 d**. [α]^20^_D_ +10.0 (c 0.5, H_2_O). ^1^H NMR (400 MHz, MeOD) δ 8.31 (d, *J*=4.8 Hz, 2H, Ar−H), 6.60 (t, *J*=4.8 Hz, 1H, Ar−H), 4.32 (t, *J*=10.1 Hz, 1H, H‐5), 4.11 (dd, *J*=10.1, 1.2 Hz, 1H, H‐6), 3.91–3.75 (m, 5H, H‐9, piperazine ×4), 3.68 (ddd, *J*=8.7, 5.8, 2.7 Hz, 1H, H‐8), 3.62 (dd, *J*=11.1, 5.9 Hz, 1H, H‐9), 3.48 (dd, *J*=9.1, 1.2 Hz, 1H, H‐7), 3.32 (m, 1H, H‐4), 3.06–2.98 (m, 2H, piperazine ×2), 2.77–2.68 (m, 2H, piperazine ×2), 2.07–1.91 (m, 5H, H‐3eq, H‐3ax, NHCOCH_3_). ^13^C NMR (101 MHz, MeOD) δ 176.6, 174.1, 166.7, 162.8, 159.0, 111.4, 97.0, 72.4, 72.0, 70.9, 65.1, 63.0, 48.8, 47.7, 44.9, 31.8, 22.8. ESI‐HRMS (m/z): calcd. for C_19_H_29_N_5_O_8_+H^+^ [M+H]+, 456.2096; found, 456.2094.

**5‐Acetamido‐4‐(4‐phenylpiperidin‐1‐yl)‐3,4,5‐ trideoxy‐D‐glycero‐β‐D‐galacto‐nonulosonic acid (3 e)** Compound **2 e** (40 mg, 0.0675 mmol) was treated with LiOH (10 mg, 0.401 mmol) as in general procedure B. Purification afforded 21 mg (69 %) of compound **3 e**. [α]^20^_D_ −39.0 (c 1, H_2_O) ^1^H NMR (400 MHz, DMSO) δ 8.02 (d, *J*=8.5 Hz, 1H), 7.39–7.09 (m, 5H), 4.74–4.40 (m, 1H), 4.35–4.07 (m, 3H), 3.88 (d, *J*=9.8 Hz, 1H), 3.60 (d, *J*=10.6 Hz, 1H), 3.49 (m, 1H), 3.24 (d, *J*=9.3 Hz, 1H), 3.04 (m, 2H), 1.97–1.52 (m, 8H). ^13^C NMR (126 MHz, DMSO) δ 172.0, 170.7, 128.6, 128.4, 126.6, 126.1, 94.6, 70.1, 69.5, 63.6, 43.7, 29.9, 22.6. ESI‐HRMS (m/z): calcd. for C_22_H_32_N_2_O_8_+H^+^ [M+H]+,453.2242; found, 453.2237

**5‐Acetamido‐4‐[4‐(3‐bromophenyl)piperidin‐1‐yl]‐3,4,5‐ trideoxy‐D‐glycero‐β‐D‐galacto‐nonulosonic acid (3 f)** Compound **2 f** (18 mg, 0.0268 mmol) was treated with LiOH (3 mg, 0.134 mmol) as in general procedure B. Purification afforded 4 mg (28 %) of compound **3 f**. [α]^20^_D_ .–17.0 (c 1, MeOH). ^1^H NMR (400 MHz, MeOD) δ 7.48 (d, *J*=2.0 Hz, 1H, Ar−H), 7.43–7.36 (m, 1H, Ar−H), 7.25 (d, *J*=7.3 Hz, 2H, Ar−H), 4.57 (t, *J*=9.1 Hz, 1H, H‐5), 4.21 (d, *J*=9.6 Hz, 1H, H‐6), 3.91 (d, *J*=11.7 Hz, 1 Hz, piperidine), 3.80 (dd, *J*=10.9, 2.4 Hz, 1H, H‐9), 3.73–3.56 (m, 3H, H‐8, H‐9, piperidine), 3.48 (d, *J*=8.8 Hz, 1H, H‐7), 3.40 (m, 1H, piperidine), 3.07 (m, 1H, piperidine), 2.87 (m, 1H, piperidine), 2.25 (d, *J*=6.9 Hz, 2H, H‐3_eq_, H‐3_ax_), 2.06 (s, 6H, NHCOCH_3_, piperidine ×3), 2.00–1.81 (m, 1H, piperidine). ^13^C NMR (126 MHz, MeOD) δ 175.0, 131.5, 131.0, 130.9, 126.8, 123.7, 72.0, 70.7, 64.9, 52.5, 46.8, 45.5, 40.9, 31.5, 30.9, 22.9. ESI‐HRMS (m/z): calcd. for C_22_H_31_N_2_O_8_Br+H^+^ [M+H]+, 531.1342; found, 531.1342.

**5‐Acetamido‐4‐[4‐(4‐bromophenyl)piperidin‐1‐yl]‐3,4,5‐ trideoxy‐D‐glycero‐β‐D‐galacto‐nonulosonic acid (3 g)** Compound **2 g** (75 mg, 0.111 mmol) was treated with LiOH (13 mg, 0.558 mmol) as in general procedure B. Purification afforded 40 mg (68 %) of compound **3 g**. [α]^20^_D_ −38.0 (c 0.5, MeOH). ^1^H NMR (500 MHz, MeOD) δ 7.52–7.45 (m, 2H, Ar−H), 7.22 (d, *J*=8.0 Hz, 2H, Ar−H), 4.58 (m, 1H, H‐5), 4.21 (d, *J*=9.6 Hz, 1H, H‐6), 3.91 (m, 1H, piperidine), 3.80 (dd, *J*=11.2, 2.7 Hz, 1H, H‐9), 3.69 (ddd, *J*=9.1, 5.6, 2.7 Hz, 1H, H‐8), 3.64 (dd, *J*=11.2, 5.6 Hz, 1H, H‐9), 3.59 (m, 1H, piperidine), 3.47 (d, *J*=9.1 Hz, 1H, H‐7), 3.45–3.35 (m, 1H, piperidine), 3.08 (m, 1H, piperidine), 2.87 (m, 1H, piperidine), 2.24 (d, *J*=6.7 Hz, 2H, H‐3_eq_, H‐3_ax_), 2.05 (m, 5H, NHCOCH_3_, piperidine ×2), 1.91 (m, 1H, piperidine). ^13^C NMR (126 MHz, DMSO) δ 171.9, 170.7, 145.3, 131.2, 129.0, 119.0, 94.6, 71.6, 70.4, 70.0, 69.5, 63.6, 60.4, 49.7, 46.1, 43.5, 40.7, 32.9, 29.9, 22.6. ESI‐HRMS (m/z): calcd. for C_22_H_31_N_2_O_8_Br+H^+^ [M+H]+, 531.1283; found, 531.1296.

**5‐Acetamido‐3,4,5‐ trideoxy‐4‐[4‐(3‐trifluoromethylphenyl)piperidin‐1‐yl]‐D‐glycero‐β‐D‐galacto‐nonulosonic acid (3 h)** Compound **2 h** (8 mg, 0.0121 mmol) was treated with LiOH (1.4 mg, 0.0605 mmol) as in general procedure B. Purification afforded 6 mg (95 %) of compound **3 h**. [α]^20^_D_ +7.0 (c 1, MeOH). ^1^H NMR (400 MHz, MeOD) δ 7.62–7.49 (m, 4H, Ar−H), 4.58 (t, *J*=9.1 Hz, 1H, H‐5), 4.21 (d, *J*=9.7 Hz, 1H, H‐6), 3.91 (m, 1H, piperidine), 3.80 (dd, *J*=11.0, 2.6 Hz, 1H, H‐9), 3.70 (ddd, *J*=8.5, 5.6, 2.6 Hz, 1H, H‐8), 3.64 (dd, *J*=11.0, 5.6 Hz, 1H, H‐9), 3.59 (m, 1H, piperidine), 3.47 (d, *J*=8.5 Hz, 1H, H‐7), 3.45–3.35 (m, 1H, piperidine), 3.15–2.91 (m, 2H, piperidine ×2), 2.25 (d, *J*=6.9 Hz, 2H„ H‐3_eq_, H‐3_ax_), 2.13 (m, 3H, piperidine ×3), 2.06 (s, 3H, NHCOCH_3_), 2.00 (m, 1H, piperidine). ^13^C NMR (126 MHz, MeOD) δ 176.1, 175.0, 146.6, 132.3, 132.1, 131.8, 130.6, 126.8, 124.7, 124.5, 96.3, 71.9, 71.2, 70.7, 65.0, 52.5, 50.4, 46.7, 41.0, 31.5, 22.9. ESI‐HRMS (m/z): calcd. for C_23_H_31_N_2_O_8_F_3_+H^+^ [M+H]+, 521.2111; found, 521.2115.

**5‐Acetamido‐4‐[4‐(3‐hydroxyphenyl)piperidin‐1‐yl]‐3,4,5‐ trideoxy‐D‐glycero‐β‐D‐galacto‐nonulosonic acid (3 i)** Compound **2 i** (40 mg, 0.0657 mmol) was treated with LiOH (8 mg, 0.329 mmol) as in general procedure B. Purification afforded 15 mg (49 %) of compound **3 i**. [α]^20^_D_ −13.0 (c 1, MeOH). ^1^H NMR (400 MHz, D_2_O) δ 7.31 (t, *J*=8.2 Hz, 1H, Ar−H), 6.91 (d, *J*=7.7 Hz, 1H, Ar−H), 6.83 (dd, *J*=4.6, 1.7 Hz, 2H, Ar−H), 4.47 (s, 1H, H‐5), 4.21 (d, *J*=9.8 Hz, 1H, H‐6), 4.02–3.75 (m, 4H, H‐4, H‐8, H‐9, piperidine), 3.65 (dd, *J*=11.8, 6.1 Hz, 1H, H‐9), 3.58 (d, *J*=9.2 Hz, 1H, H‐7), 3.47 (m, 2H, piperidine), 3.19 (m, 1H, piperidine), 2.88 (m, 1H, piperidine), 2.40 (m, 1H, H‐3_eq_), 2.26–2.06 (m, 7H, H‐3_ax_, NHCOCH_3_, piperidine ×3), 1.90 (m, 1H, piperidine). ^13^C NMR (101 MHz, D_2_O) δ 176.5, 169.6, 157.3, 147.3, 131.7, 120.2, 115.3, 115.1, 71.8, 71.6, 69.7, 64.9, 64.6, 53.4, 49.5, 46.8, 40.4, 31.3, 23.8. ESI‐HRMS (m/z): calcd. for C_22_H_32_N_2_O_9_+H^+^ [M+H]+, 469.2186; found, 469.2186.

**5‐Acetamido‐4‐(4‐phenylacetylpiperazin‐1‐yl)‐3,4,5‐ trideoxy‐D‐glycero‐β‐D‐galacto‐nonulosonic acid (6 a)** TFA salt Compound **5 a** (12 mg, 0.0188 mmol) was treated with LiOH (2 mg, 0.0943 mmol) as in general procedure B. Purification afforded 8 mg (85 %) of compound **6 a**. [α]^20^_D_ +2.0 (c 1, MeOH). ^1^H NMR (500 MHz, MeOD) δ 7.78–7.06 (m, 5H, Ar−H), 4.23 (t, *J*=10.3 Hz, 1H, H‐5), 4.14 (d, *J*=10.3 Hz, 1H, H‐6), 3.84–3.41 (4H, N(CH_2_CH_2_)N ×4) 3.79 (m, 3H, Ar‐CH_2_, H‐9), 3.68 (ddd, *J*=8.6, 5.6, 2.7 Hz, 2H, H‐8, H‐4), 3.63 (dd, *J*=11.2, 5.6 Hz, 1H, H‐9), 3.52 (d, *J*=9.1 Hz, 1H, H‐7), 3.09–3.0 (m, 1H, N(CH_2_CH_2_)N ×1), 2.98–2.91 (m, 1H, N(CH_2_CH_2_)N ×1), 2.70–2.72 (m, 1H, N(CH_2_CH_2_)N ×1), 2.65–2.56 (m, 1H, N(CH_2_CH_2_)N ×1), 2.14–2.06 (m, 1H, H‐3_eq_), 1.98 (s, 3H, HNCOCH_3_), 1.88 (t, *J*=12.4 Hz, 1H, H‐3_ax_). ^13^C NMR (126 MHz, MeOD) δ 174.6, 173.9, 172.2, 136.2, 129.9, 129.8, 128.0, 96.2, 72.4, 71.8, 70.2, 64.8, 63.0, 47.4, 42.2, 41.2, 31.4, 22.8. ESI‐HRMS (m/z): calcd. for C_23_H_33_N_3_O_9_+H^+^ [M+H]+, 496.2286; found, 496.2295.

**5‐Acetamido‐4‐(4‐benzoylpiperazin‐1‐yl)‐3,4,5‐ trideoxy‐D‐glycero‐β‐D‐galacto‐nonulosonic acid (6 b)** TFA salt Compound **5 b** (42 mg, 0.0676 mmol) was treated with LiOH (8 mg, 0.338 mmol) as in general procedure B. Purification afforded 29 mg (88 %) of compound **6 b**. [α]^20^_D_ +5.0 (c 1, MeOH). ^1^H NMR (500 MHz, MeOD) δ 7.54–7.40 (m, 5H, Ar−H), 4.26 (t, *J*=10.4 Hz, 1H, H‐5), 4.16 (dd, *J*=10.4, 1.3 Hz, 1H, H‐6), 3.95–3.39 (4H, N(CH_2_CH_2_)N ×4), 3.80 (dd, *J*=11.2, 2.8 Hz, 1H, H‐9), 3.70 (ddd, *J*=8.7, 5.6, 2.8 Hz, 1H, H‐8), 3.63 (dd, *J*=11.2, 5.6 Hz, 1H, H‐9), 3.55 (dd, *J*=8.7, 1.3 Hz, 1H, H‐7), 3.50–3.44 (m, 1H, H‐4), 3.21–3.02 (m, 2H, N(CH_2_CH_2_)N ×2), 2.92–2.66 (m, 2H, N(CH_2_CH_2_)N ×2), 2.18 (dd, *J*=12.2, 3.9 Hz, 1H, H‐3_eq_), 2.02 (s, 3H, HNCOCH_3_), 1.97 (t, *J*=12.2 Hz, 1H, H‐3_ax_). ^13^C NMR (126 MHz, MeOD) δ 174.6, 173.7, 172.5, 163.0, 136.4, 131.3, 129.8, 128.1, 96.2, 72.4, 71.9, 70.3, 64.8, 49.6, 49.3, 47.5, 31.5, 22.8, 21.2. ESI‐HRMS (m/z): calcd. for C_22_H_31_N_3_O_9_+H^+^ [M+H]+, 482.2133; found, 482.2148.

**5‐Acetamido‐4‐[4‐(2,3‐dichlorobenzoyl)piperazin‐1‐yl]‐3,4,5‐ trideoxy‐D‐glycero‐β‐D‐galacto‐nonulosonic acid (6 c)** Compound **5 c** (50 mg, 0.0724 mmol) was treated with LiOH (9 mg, 0.362 mmol) as in general procedure B. Purification afforded 13 mg (33 %) of compound **6 c**. [α]^20^_D_ −7.0 (c 1, MeOH). ^1^H NMR (500 MHz, MeOD) δ 7.65 (dd, *J*=8.0, 1.5 Hz, 1H, Ar−H), 7.42 (td, *J*=7.8, 1.0 Hz, 1H, Ar−H), 7.35 (dt, *J*=7.8, 1.7 Hz, 1H, Ar−H), 4.31 (q, *J*=10.2 Hz, 1H, H‐5), 4.24–4.17 (m, 1H, H‐6), 4.16–3.96 (m, 1H, N(CH_2_CH_2_)N ×1), 3.91–3.75 (m, *J*=11.1, 2.4 Hz, 2H, H‐9, N(CH_2_CH_2_)N x1), 3.72–3.67 (m, 1H, H‐8), 3.67–3.58 (m, *J*=11.2, 5.5, 1.9 Hz, 2H, H‐4, H‐9), 3.57–3.53 (m, 1H, H‐7), 3.52–3.19 (m, 4H, N(CH_2_CH_2_)N ×4), 3.13 ‐ 3.01 (m, 1H, N(CH_2_CH_2_)N ×1), 2.97–2.82 (m, 1H, N(CH_2_CH_2_)N ×1), 2.28–2.21 (m, 1H, H‐3_eq_), 2.10–1.97 (m, 4H, H‐3_ax_, HNCOCH_3_). ^13^C NMR (126 MHz, MeOD) δ 174.9, 172.9, 167.9, 138.1, 134.6, 132.7, 129.9, 129.6, 127.5, 95.9, 72.1, 71.8, 70.0, 64.7, 50.3, 47.2, 31.5, 3.4, 22.9, 22.9. ESI‐HRMS (m/z): calcd. for C_22_H_29_N_3_O_9_Cl_2_+H^+^ [M+H]+, 550.1365; found, 550.1359.

**5‐Acetamido‐4‐[4‐(3,5‐dimethoxybenzoyl)piperazin‐1‐yl]‐3,4,5‐ trideoxy‐D‐glycero‐β‐D‐galacto‐nonulosonic acid (6 d)** Compound **5 d** (50 mg, 0.0733 mmol) was treated with LiOH (9 mg, 0.368 mmol) as in general procedure B. Purification afforded 5 mg (13 %) of compound **6 d**. [α]^20^_D_ −2.0 (c 1, MeOH). ^1^H NMR (500 MHz, MeOD) δ 6.59 (m, 3H, Ar−H), 4.35 (t, *J*=10.3 Hz, 1H, H‐5), 4.24 (dd, *J*=10.3, 1.3 Hz, 1H, H‐6), 4.07–3.51 (4H, N(CH_2_CH_2_)N ×4), 3.83–3.77 (m, 7H, H‐9, Ar‐OCH_3_ ×2), 3.72–3.68 (m, 2H, H‐4, H‐8), 3.65 (dd, *J*=11.2, 5.5 Hz, 1H, H‐9), 3.56 (dd, *J*=9.2, 1.3 Hz, 1H, H‐7), 3.50–3.36 (m, 2H, N(CH_2_CH_2_)N ×2), 3.15 ‐ 2.91 (m, 2H, N(CH_2_CH_2_)N ×2), 2.30 (dd, *J*=12.7, 3.7 Hz, 1H, H‐3_eq_), 2.10 (t, *J*=12.5 Hz, 1H, H‐3_ax_), 2.04 (s, 3H, HNCOCH_3_). ^13^C NMR (126 MHz, MeOD) δ 175.0, 172.8, 172.1, 162.7, 137.7, 105.9, 102.9, 95.8, 72.0, 71.8, 69.9, 64.7, 56.0, 49.9, 49.1, 47.1, 31.4, 22.9. ESI‐HRMS (m/z): calcd. for C_24_H_35_N_3_O_11_+H^+^ [M+H]+, 542.2347; found, 542.2350

**5‐Acetamido‐4‐[4‐(L‐phenylalanyl)piperazin‐1‐yl]‐3,4,5‐trideoxy‐D‐glycero‐β‐D‐galacto‐nonulosonic acid (6 e)** Starting material **4** (100 mg, 0.193 mmol) was dissolved in dry DCM (2 mL), then Fmoc‐Phe‐OH (150 mg, 0.386 mmol), HATU (220 mg, 0.579 mmol) and DIPEA (0.202 mL, 1.158 mmol) were added. The reaction was completed after 2 hours. The reaction was concentrated, dissolved in EtOAc, washed with water and brine and dried. The crude was then dissolved in ACN‐H_2_O and treated with LiOH (8 mg) as in general procedure B. Purification afforded 9 mg (9 % over two steps) of **6 e**. [α]^20^_D_ +34.0 (c 0.5, MeOH). ^1^H NMR (500 MHz, MeOD) δ 7.62–7.25 (m, 5H, Ar−H), 4.67 (dd, *J*=9.7, 6.0 Hz, 1H, OCC*H*NH2), 4.24 (t, *J*=10.4 Hz, 1H, H‐5), 4.17 (dd, *J*=10.4, 1.3 Hz, 1H, H‐6), 4.02–3.89 (m, 1H, N(CH_2_CH_2_)N ×1), 3.80 (dd, *J*=11.2, 2.8 Hz, 1H, H‐9), 3.70 (ddd, *J*=8.7, 5.5, 2.8 Hz, 1H, H‐8), 3.63 (dd, *J*=11.3, 5.5 Hz, 1H, H‐9), 3.58–3.38 (m, 4H, H‐4, H‐7, N(CH_2_CH_2_)N ×2), 3.34–3.12 (m, 3H, Ar‐CH, N(CH_2_CH_2_)N ×4), 3.05 (dd, *J*=13.1, 9.7 Hz, 1H, Ar‐CH), 2.72–2.63 (s, 1H, N(CH_2_CH_2_)N ×1), 2.58–2.48 (s, 1H, N(CH_2_CH_2_)N ×1), 2.06–1.93 (m, 4H, H‐3_eq_, HNCOCH_3_), 1.86 (t, *J*=12.5 Hz, 1H, H‐3_ax_). ^13^C NMR (126 MHz, MeOD) δ 174.7, 172.9, 168.2, 135.4, 131.0, 130.4, 129.3, 95.8, 72.1, 71.8, 69.9, 64.7, 63.7, 51.5, 49.3, 47.0, 45.0, 38.9, 31.2, 22.8. ESI‐HRMS (m/z): calcd. for C_24_H_36_N_4_O_9_+H^+^ [M+H]+, 524.2482; found 524.2481.

**5‐Acetamido‐4‐{4‐[3‐(3‐pyridyl)‐L‐alanyl]piperazin‐1‐yl}‐3,4,5‐trideoxy‐D‐glycero‐β‐D‐galacto‐nonulosonic acid (6 f)** Starting material **4** (100 mg, 0.193 mmol) was dissolved in dry DCM (2 mL), then Fmoc‐β‐(3‐pyridyl)‐Ala‐OH (150 mg, 0.386 mmol), HATU (220 mg, 0.579 mmol) and DIPEA (0.202 mL, 1.158 mmol) were added. The reaction was completed after 2 hours. The reaction was concentrated, dissolved in EtOAc, washed with water and brine and dried. The crude was then dissolved in ACN‐H_2_O and treated with LiOH (7 mg) as in general procedure B. Purification afforded 4 mg (4 %) of compound **6 f**. [α]^20^_D_ +4.0 (c 1, MeOH).^1^H NMR (500 MHz, MeOD) δ 8.73–8.52 (m, 2H, Ar−H), 7.99 (d, *J*=7.9 Hz, 1H, Ar−H), 7.65 (t, *J*=6.6 Hz, 1H, Ar−H), 4.77 (t, *J*=7.1 Hz, 1H, OCC*H*NH2), 4.22 (t, *J*=10.4 Hz, 1H, H‐5), 4.15 (d, *J*=9.5 Hz, 1H, H‐6), 3.84 ‐ 3.16 (4H, piperazine ×4), 3.83–3.76 (m, 1H, H‐9), 3.70 (ddd, *J*=8.7, 5.6, 2.8 Hz, 1H, H‐8), 3.64 (dd, *J*=11.3, 5.6 Hz, 3H, H‐9), 3.54 (dd, *J*=9.1, 1.3 Hz, 1H, H‐7), 3.47–3.37 (m, 1H, H‐4), 3.30–3.16 (m, 2H, Ar‐CH_2_), 3.03 (m, 1H, piperazine), 2.64 (m, 3H, piperazine ×3), 2.08–2.02 (m, 1H, H‐3_eq_), 1.99 (s, 3H, HNCOCH_3_), 1.87 (t, *J*=12.5 Hz, 1H, H‐3_ax_). ^13^C NMR (126 MHz, MeOD) δ 174.5, 173.3 167.5, 155.1, 149.4, 148.0, 142.0, 126.5, 96.1, 72.4, 71.8, 70.2, 64.7, 57.5, 5.4, 47.4, 44.2, 35.2, 31.2, 22.8, 20.9. ESI‐HRMS (m/z): calcd. for C_23_H_35_N_5_O_9_+H^+^ [M+H]+, 526.2512; found, 526.2513.

### Biological assays and computational work

**nanoDSF**: Melting points of protein‐inhibitor complexes were measured on a Prometheus NT.48 instrument from NanoTemper Technologies. Solutions containing PmSiaT at 2 μM in PBS with 0.0174 % (w/v) of N‐Dodecyl β‐D‐maltoside (DDM) for *Pm*SiaT, and the ligand at 1.25 mM were loaded on standard capillaries by NanoTemper. Similarly, the protein concentration for *Fn*SiaP was 4 μM in HEPES (4‐(2‐hydroxyethyl)‐1‐piperazineethanesulfonic acid) buffer with a ligand concentration of 0.2 mM, while for *Hd*SatA the protein concentration was 2 μM in HEPES, with a ligand concentration of 0.04 mM. The temperature was increased from 25 to 95 °C at a ramp rate of 1 °C/min. The fluorescence was recorded at 330 nm, 350 nm and as a ratio of the two wavelengths. The intensity ratio and its first derivative were calculated with the manufacturer's software (PR.ThermControl, version 2.1.2). All nanoDSF data can be found at **Table S1‐3**.

**Isothermal titration calorimetry**: Isothermal titration calorimetric experiments were performed on an Microcal PEAQ‐ITC (Malvern) instrument at 25 °C using standard instrument settings (reference power 10 μcal s^−1^, stirring speed 750 rpm, feedback mode high). Protein solutions were dialysed against ITC buffer (50 mM Tris‐HCl, 150 mM NaCl, with 0.0174 % (w/v) DDM, pH 8.0 at 5 °C) prior to the experiments and all samples were prepared using the dialysate buffer to minimise dilution effects. Protein concentrations were determined spectrophotometrically with the specific absorbance at 280 nm employing an extinction coefficient of 76,445 mol^−1^ cm^−1^ for *Pm*SiaT. Binding affinities of *Pm*SiaT inhibitors in the μM range necessitated a low c titration setup, which nevertheless allows for the reliable determination of affinity, enthalpic and entropic contributions.[^31^, ^32^] In a typical experiment, a 0.3–1.2 mM inhibitor solution was titrated to a solution containing 15–30 μM of *Pm*SiaT to ensure >90 % saturation. Baseline correction, peak integration, and non‐linear regression analysis of experimental data was performed using the NITPIC (version 1.2.2.)^[33]^ and SEDPHAT (version 12.1b)^[34]^ software packages. The stoichiometry parameter was manually constrained to a value of 1. Experiments were performed in triplicate and the 68 % confidence intervals from global fitting of three experiments were calculated as an estimate of experimental error. All titration curves can be found in Figure S10–15.

**Molecular dynamics simulations**: Molecular dynamics simulations were performed with the OPLS4 force field in Desmond (Schrödinger Release 2022–1: Desmond Molecular Dynamics System, D. E. Shaw Research, New York, NY, 2017; Maestro‐Desmond Interoperability Tools, Schrödinger, New York, NY, 2017) using default settings except for the length of the simulation and the use of light harmonic constraints (1 kcal mol^−1^ Å^−1^) on all helix backbone atoms. Starting conformation of **6 c** in complex with *Pm*SiaT was built by adding the piperazinyl functionality to C4 of Neu5Ac in the *Pm*SiaT X‐ray structure (pdb ID 5NV9).^[24]^ The complexes were then subjected to 100 ns molecular dynamics simulations. Molecular images were generated using PyMOL v1.7 (Schrodinger LLC).

## Conflict of interest

The authors declare no conflict of interest.

1

## Supporting information

As a service to our authors and readers, this journal provides supporting information supplied by the authors. Such materials are peer reviewed and may be re‐organized for online delivery, but are not copy‐edited or typeset. Technical support issues arising from supporting information (other than missing files) should be addressed to the authors.

Supporting Information

## Data Availability

The data that support the findings of this study are available in the supplementary material of this article.
