# Supplementary material for: Sialic Acid 4‐N‐Piperazine and Piperidine Derivatives Bind with High Affinity to the P. mirabilis Sialic Acid Sodium Solute Symporter
Source: ChemMedChem. 2022 Oct 13;17(23):e202200351. doi: 10.1002/cmdc.202200351 (PMC10092485; doi:10.1002/cmdc.202200351)

ChemMedChem

Supporting Information

Sialic Acid 4-*N*-Piperazine and Piperidine Derivatives Bind with High Affinity to the *P. mirabilis* Sialic Acid Sodium Solute Symporter

Tiago Bozzola, Richard E. Johnsson, Ulf J. Nilsson, and Ulf Ellervik*

Contents

nanoDSF data 2

ITC data 4

NMR spectra. ^{1}H- and ^{13}C-NMR spectra 6

nanoDSF data

Table S1. nanoDSF data for *Pm*SiaT.

Sample	Tm Replicate 1	Tm Replicate 2	Tm Replicate 3	Tm Replicate 4	Tm Average	SD	ΔTm from Neu5Ac
*Pm*SiaT	46.7°C	46.6°C	46.5°C	46.5°C	46.6	0.1	-2.8
Neu5Ac	49.4°C	49.4°C	49.3°C	49.4°C	49.4	0.0	0.0
**3a**	49.5°C	49.4°C	49.4°C	49.5°C	49.5	0.1	0.1
**3b**	54.8°C	55.1°C	54.7°C	54.6°C	54.8	0.2	5.4
**3c**	53.6°C	54.6°C	54.0°C	54.0°C	54.1	0.4	4.7
**3d**	56.3°C	56.8°C	55.5°C	55.5°C	56.0	0.6	6.6
**3e**	57.2°C	56.9°C	56.6°C	56.4°C	56.8	0.3	7.4
**3f**	58.7°C	58.9°C	58.9°C	58.8°C	58.8	0.1	9.4
**3g**	57.5°C	57.6°C	57.6°C	57.6°C	57.5	0.0	8.1
**3h**	57.6°C	57.7°C	57.6°C	57.6°C	57.6	0.1	8.2
**3i**	53.6°C	54.6°C	54.5°C	54.2°C	54.2	0.4	4.8
**6a**	53.6°C	53.5°C	52.9°C	53.0°C	53.3	0.4	3.9
**6b**	55.4°C	55.3°C	55.2°C	55.3°C	55.3	0.1	5.9
**6c**	60.9°C	60.8°C	60.3°C	60.3°C	60.6	0.3	11.2
**6d**	54.5°C	54.4°C	54.1°C	54.2°C	54.3	0.2	4.9
**6e**	48.0°C	47.4°C	47.7°C	47.8°C	47.7	0.3	-1.7
**6f**	47.4°C	46.9°C	47.0°C	47.1°C	47.1	0.2	-2.3

Table S2. nanoDSF data for *Fn*SiaP.

Sample	Tm Replicate 1	Tm Replicate 2	Tm Replicate 3	Tm Replicate 4	Tm Average	SD	ΔTm from Neu5Ac
*Fn*SiaP	60.5°C	60.7°C	60.5°C	60.2°C	60.5°C	0.2	-5.1
Neu5Ac	65.0°C	65.0°C	66.2°C	66.2°C	65.6°C	0.7	0.0
**3a**	60.3°C	60.2°C	60.4°C	60.6°C	60.4°C	0.2	-5.2
**3b**	61.1°C	60.9°C	60.7°C	60.5°C	60.8°C	0.2	-4.8
**3c**	59.8°C	59.6°C	59.6°C	59.5°C	59.6°C	0.1	-6.0
**3d**	60.7°C	60.7°C	61.2°C	61.9°C	61.1°C	0.6	-4.5
**3e**	60.1°C	60.0°C	59.9°C	60.3°C	60.1°C	0.2	-5.5
**3f**	60.1°C	60.4°C	60.2°C	59.9°C	60.2°C	0.2	-5.4
**3g**	60.3°C	60.2°C	60.9°C	60.7°C	60.5°C	0.3	-5.1
**3h**	60.3°C	60.7°C	60.5°C	60.3°C	60.5°C	0.2	-5.1
**3i**	60.4°C	60.8°C	60.5°C	60.1°C	60.4°C	0.3	-5.2
**6a**	60.5°C	60.4°C	60.9°C	60.7°C	60.6°C	0.2	-5.0
**6b**	60.4°C	60.5°C	60.1°C	60.5°C	60.4°C	0.2	-5.2
**6c**	60.2°C	60.5°C	60.5°C	60.3°C	60.3°C	0.1	-5.3
**6d**	60.5°C	60.9°C	60.1°C	61.0°C	60.6°C	0.4	-5.0
**6e**	60.0°C	60.3°C	60.3°C	60.4°C	60.2°C	0.2	-5.4
**6f**	60.8°C	60.7°C	61.4°C	61.1°C	61.0°C	0.3	-4.6

Table S3. nanoDSF data for *Hd*SatA.

Sample	Tm Replicate 1	Tm Replicate 2	Tm Replicate 3	Tm Replicate 4	Tm Average	SD	ΔTm from Neu5Ac
*Hd*SatA	65.5°C	65.4°C	65.6°C	65.5°C	65.5°C	0.1	-6.0
Neu5Ac	71.2°C	71.5°C	71.7°C	71.8°C	71.5°C	0.3	0.0
**3a**	65.3°C	65.3°C	65.1°C	65.1°C	65.2°C	0.1	-6.3
**3b**	65.3°C	65.3°C	65.4°C	65.3°C	65.3°C	0.0	-6.2
**3c**	65.7°C	65.7°C	65.8°C	66.7°C	66.0°C	0.5	-5.5
**3d**	65.3°C	65.3°C	65.3°C	65.4°C	65.3°C	0.0	-6.2
**3e**	65.3°C	65.4°C	65.2°C	65.3°C	65.3°C	0.1	-6.2
**3f**	65.5°C	65.4°C	65.3°C	65.3°C	65.4°C	0.1	-6.1
**3g**	65.4°C	65.3°C	65.2°C	65.3°C	65.3°C	0.1	-6.2
**3h**	65.4°C	65.4°C	65.4°C	65.4°C	65.4°C	0.0	-6.1
**3i**	65.3°C	65.3°C	65.3°C	65.3°C	65.3°C	0.0	-6.2
**6a**	65.4°C	65.3°C	65.4°C	65.3°C	65.3°C	0.0	-6.2
**6b**	65.4°C	65.4°C	65.4°C	65.3°C	65.4°C	0.0	-6.1
**6c**	65.4°C	65.4°C	65.3°C	65.3°C	65.3°C	0.1	-6.2
**6d**	65.4°C	65.3°C	65.3°C	65.2°C	65.3°C	0.1	-6.2
**6e**	64.4°C	64.3°C	64.2°C	64.2°C	64.3°C	0.1	-7.2
**6f**	65.3°C	65.3°C	65.2°C	65.1°C	65.2°C	0.1	-6.3

ITC data

Table S4. ITC data with confidence intervals for dissociation constant (K_d, µM) and binding thermodynamics in kJ/mol of compounds **3i**, **3f** and **6c**.

Compound	K_d low	K_d high	Δ*G*° low	Δ*G*° high	Δ*H*° low	Δ*H*° high	-TΔ*S*° low	-TΔ*S*° high
**3i**	13.5	25.1	-27.8	-26.3	36.3	64.8	-92.6	-62.6
**3f**	0.69	4.57	-35.2	-30.5	32.2	55.0	-90.2	-62.7
**6c**	0.17	1.20	-38.6	-33.8	28.5	37.6	-76.2	-62.3

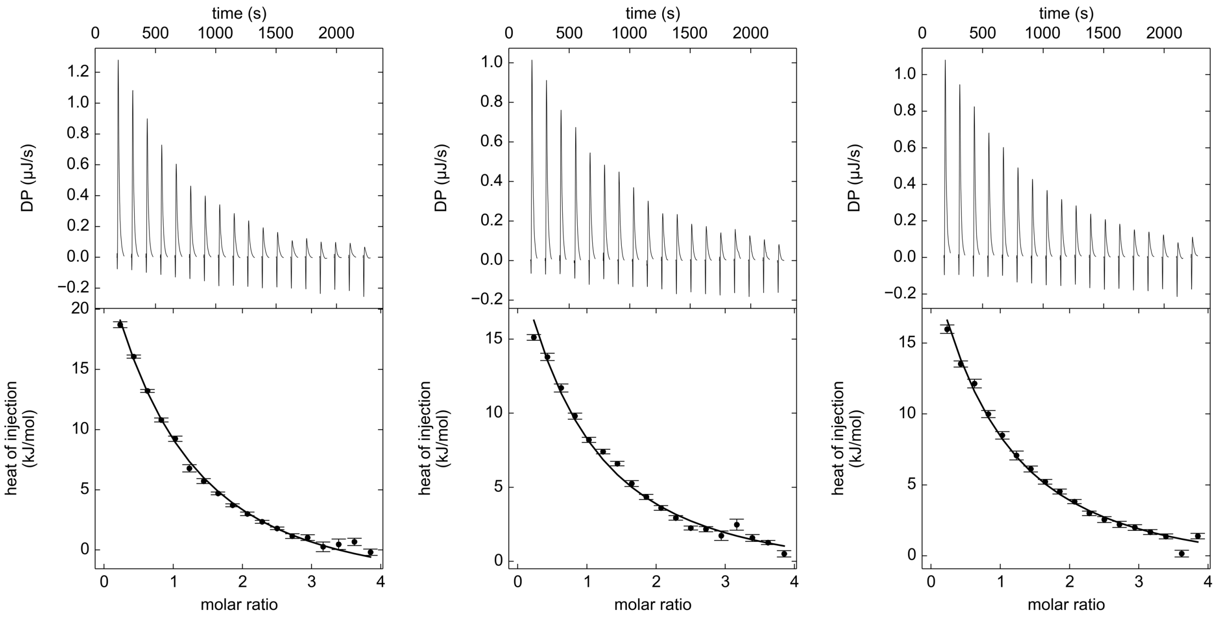

Figure S1. Three ITC titrations for compound **3i** with *Pm*SiaT.

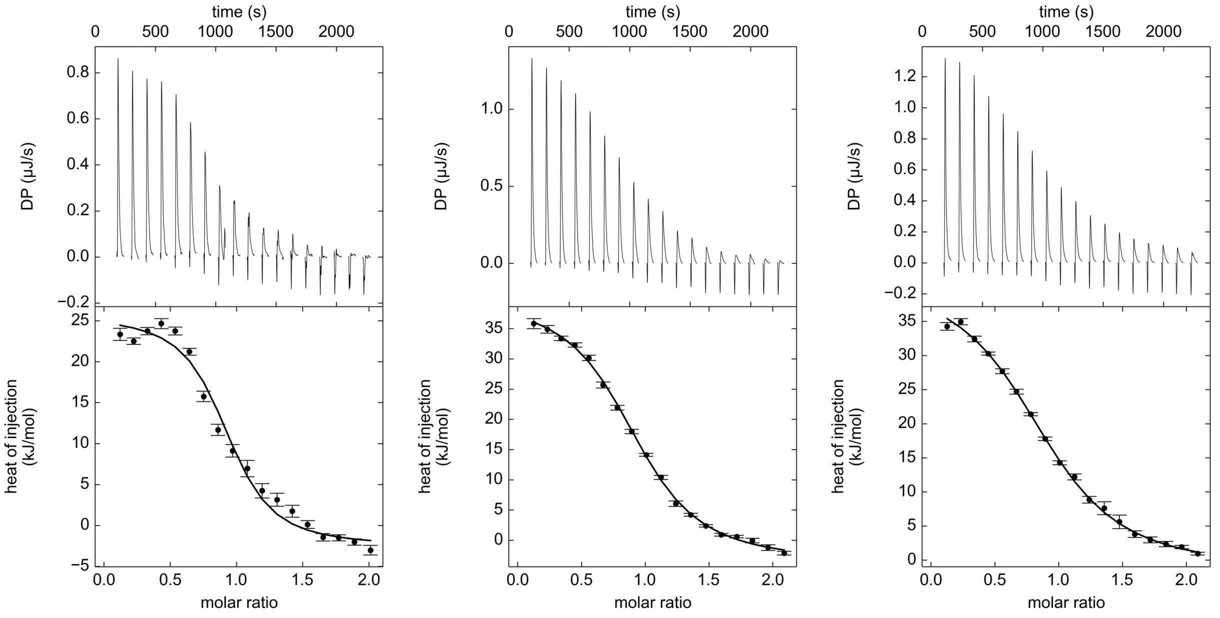

Figure S2. Three ITC titrations of compound **3e** with *Pm*SiaT.

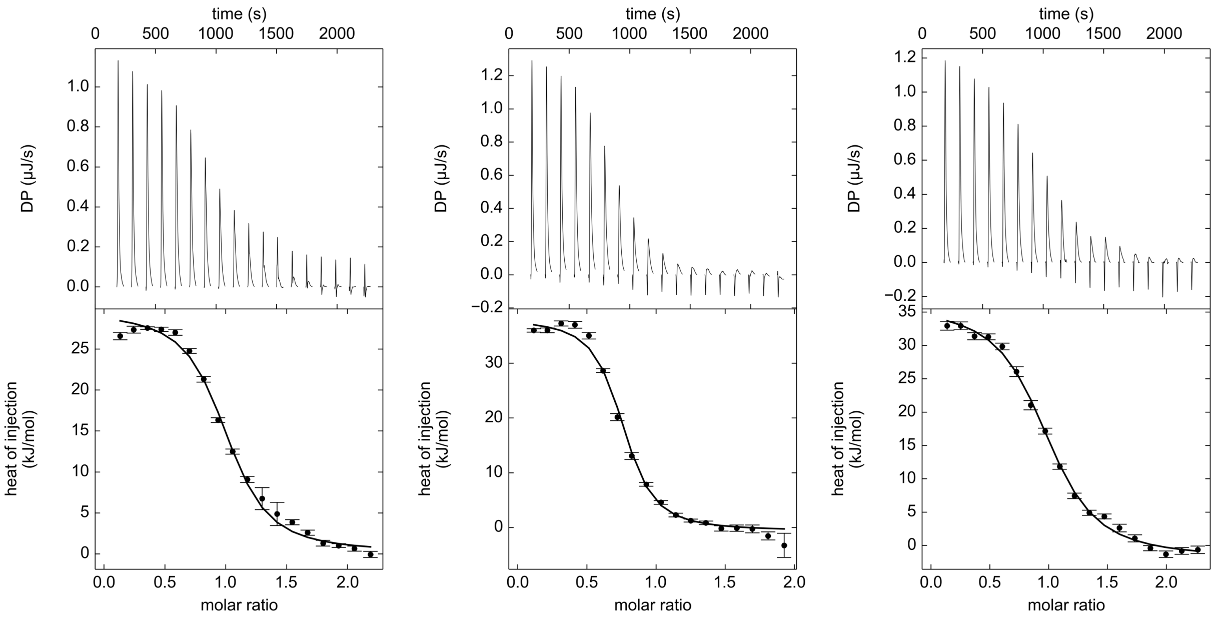

Figure S3. Three ITC titrations for compound **6c** with *Pm*SiaT.

NMR spectra. ^{1}H- and ^{13}C-NMR spectra of compound 2b

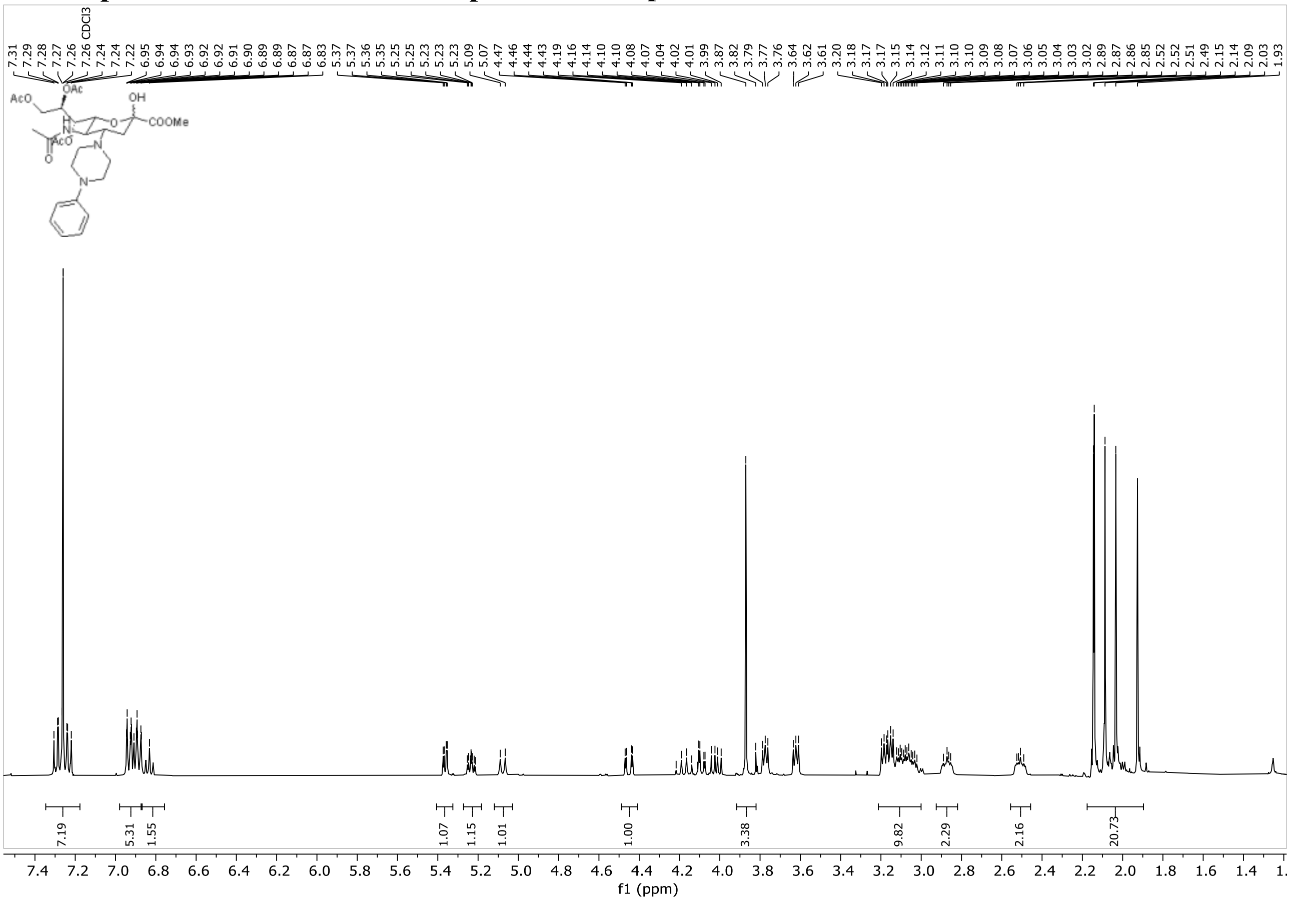

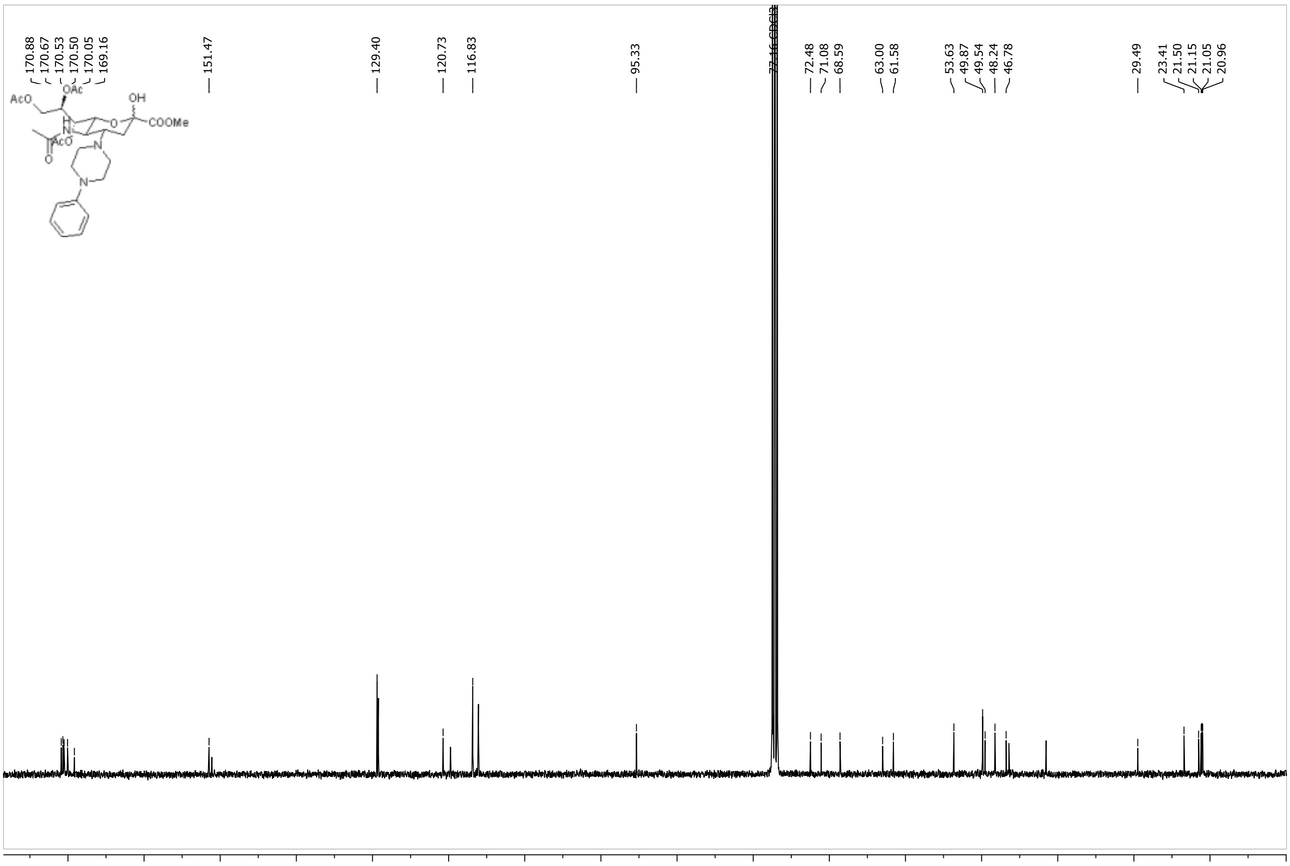

^{1}H- and ^{13}C-NMR spectra of compound 2c

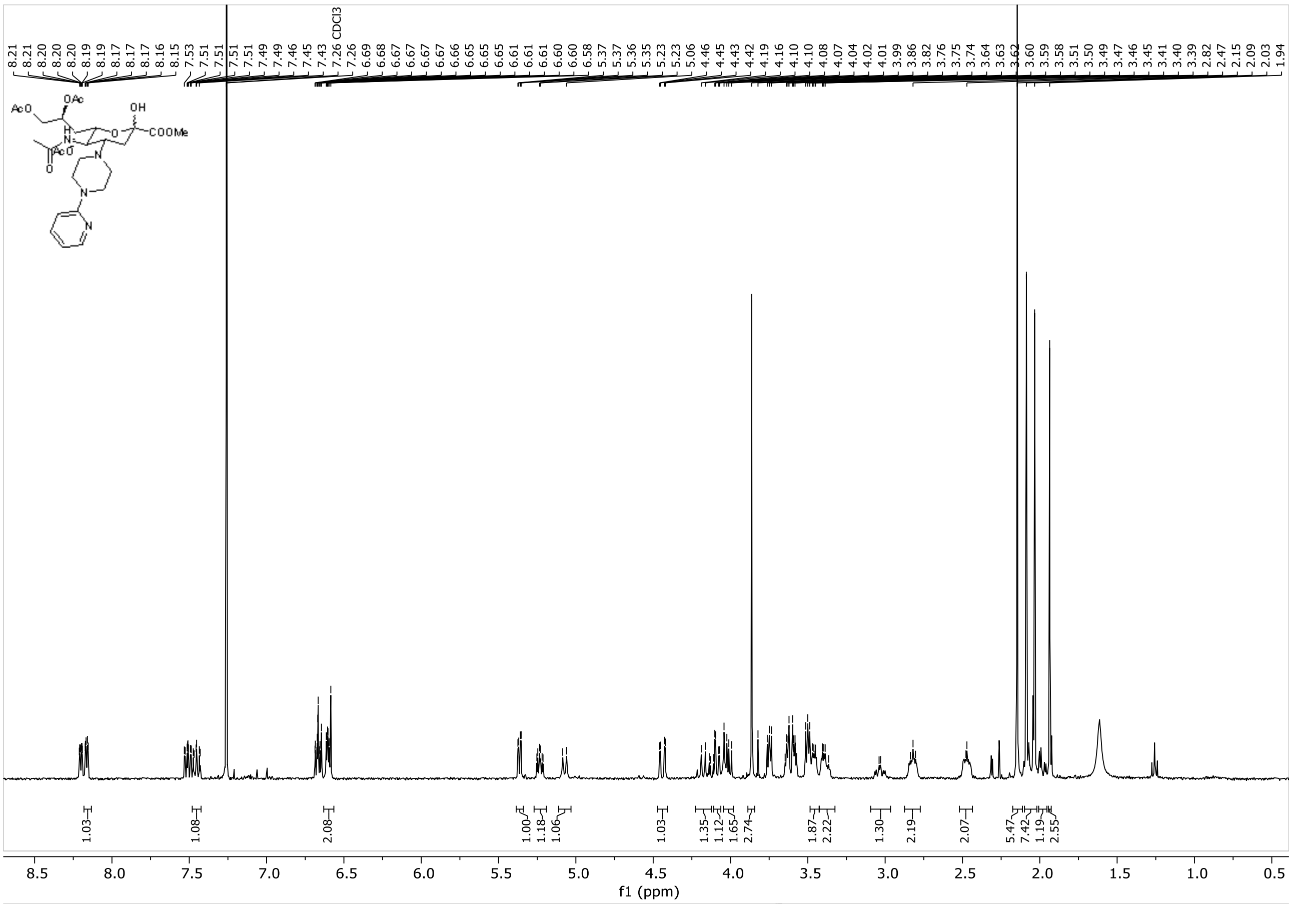

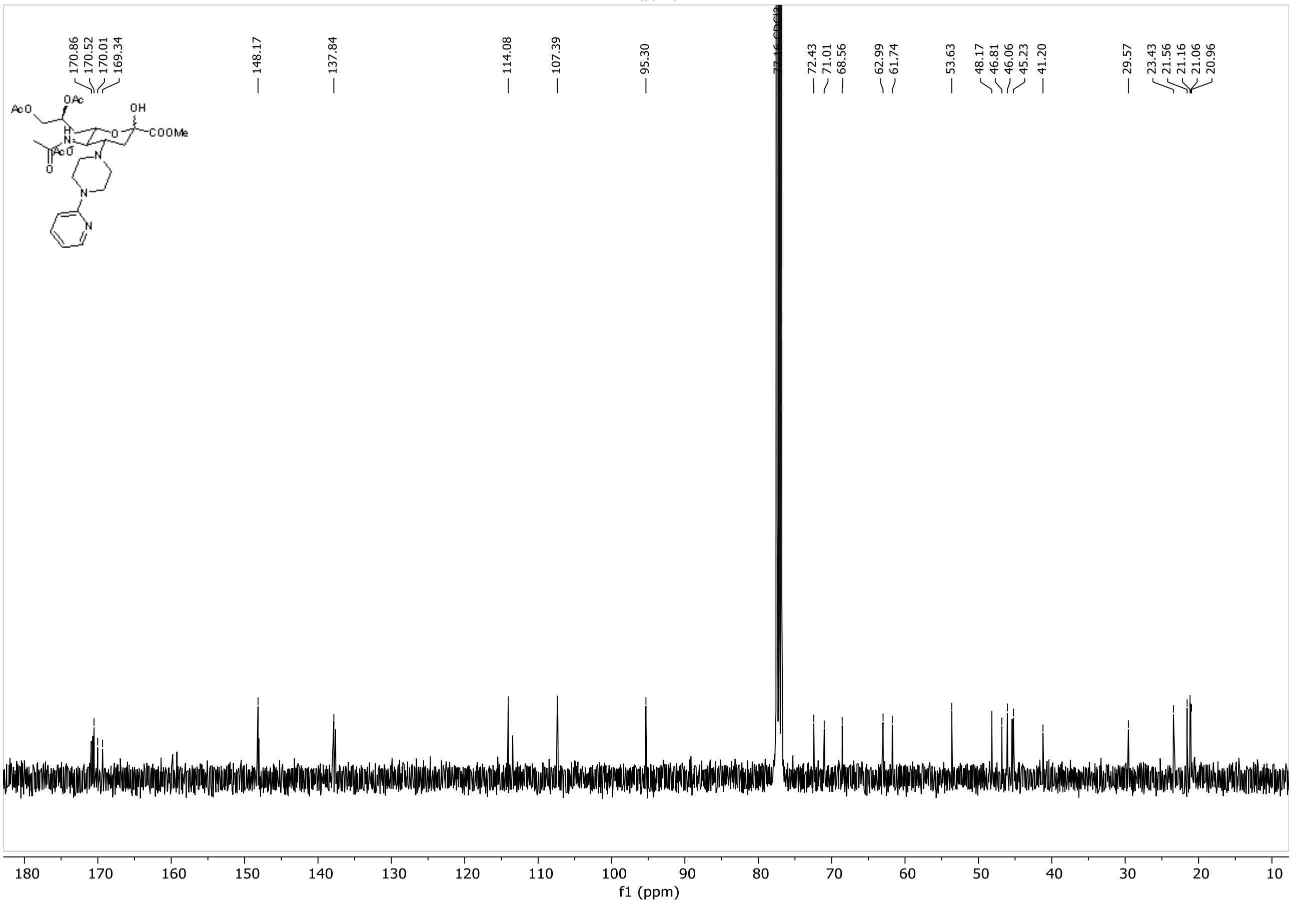

^{1}H- and ^{13}C-NMR spectra of compound 2d

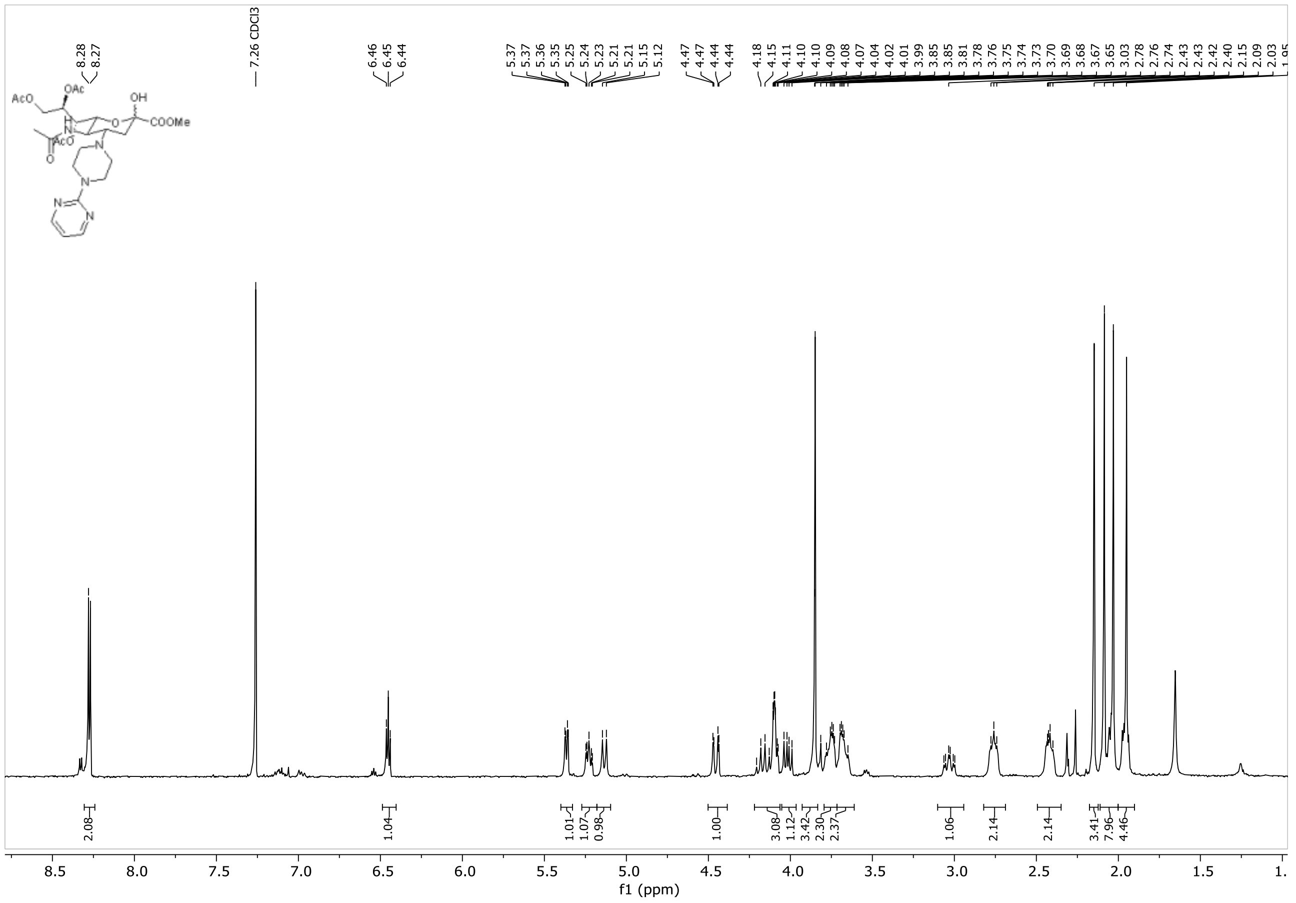

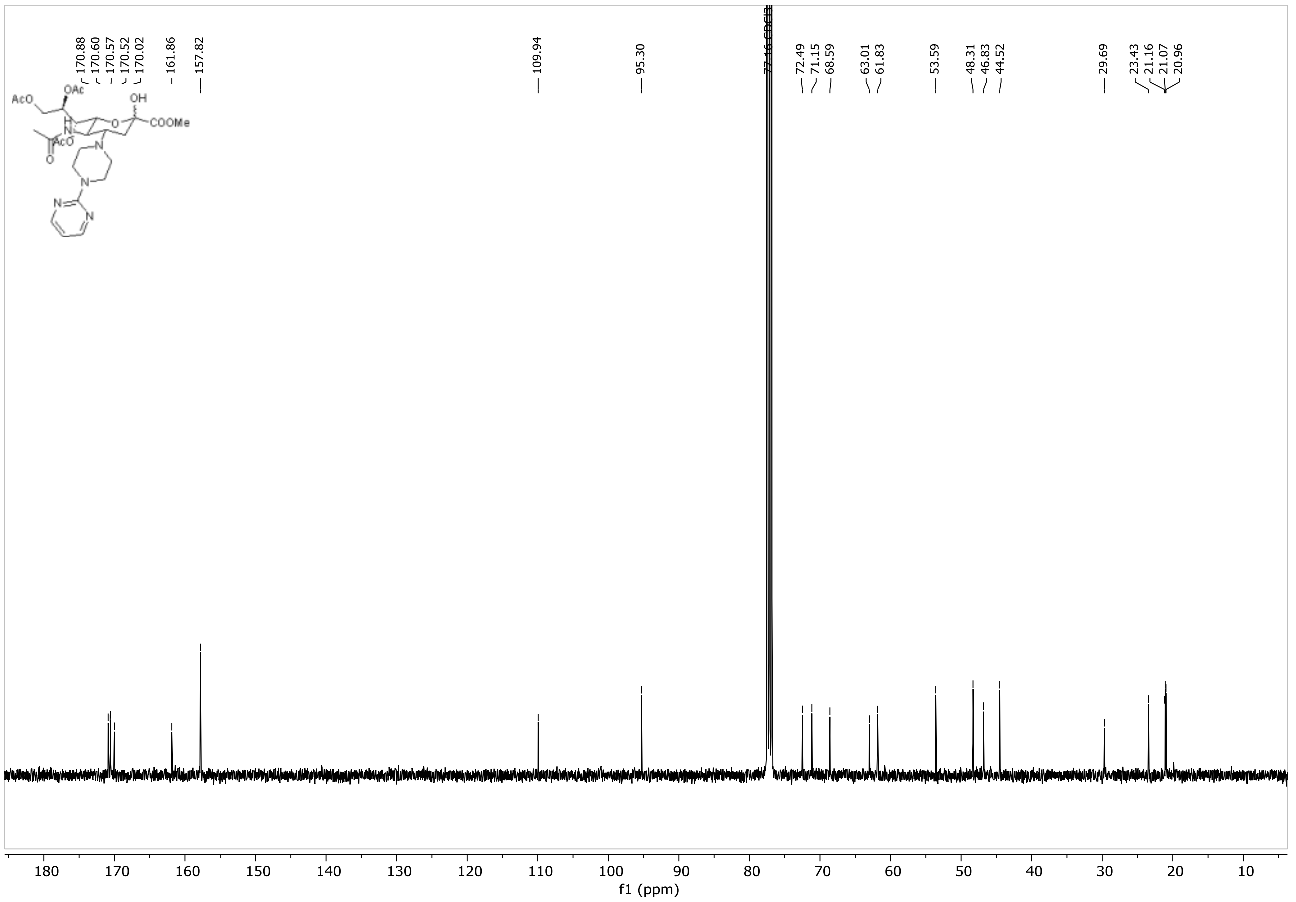

^{1}H- and ^{13}C-NMR spectra of compound 2e

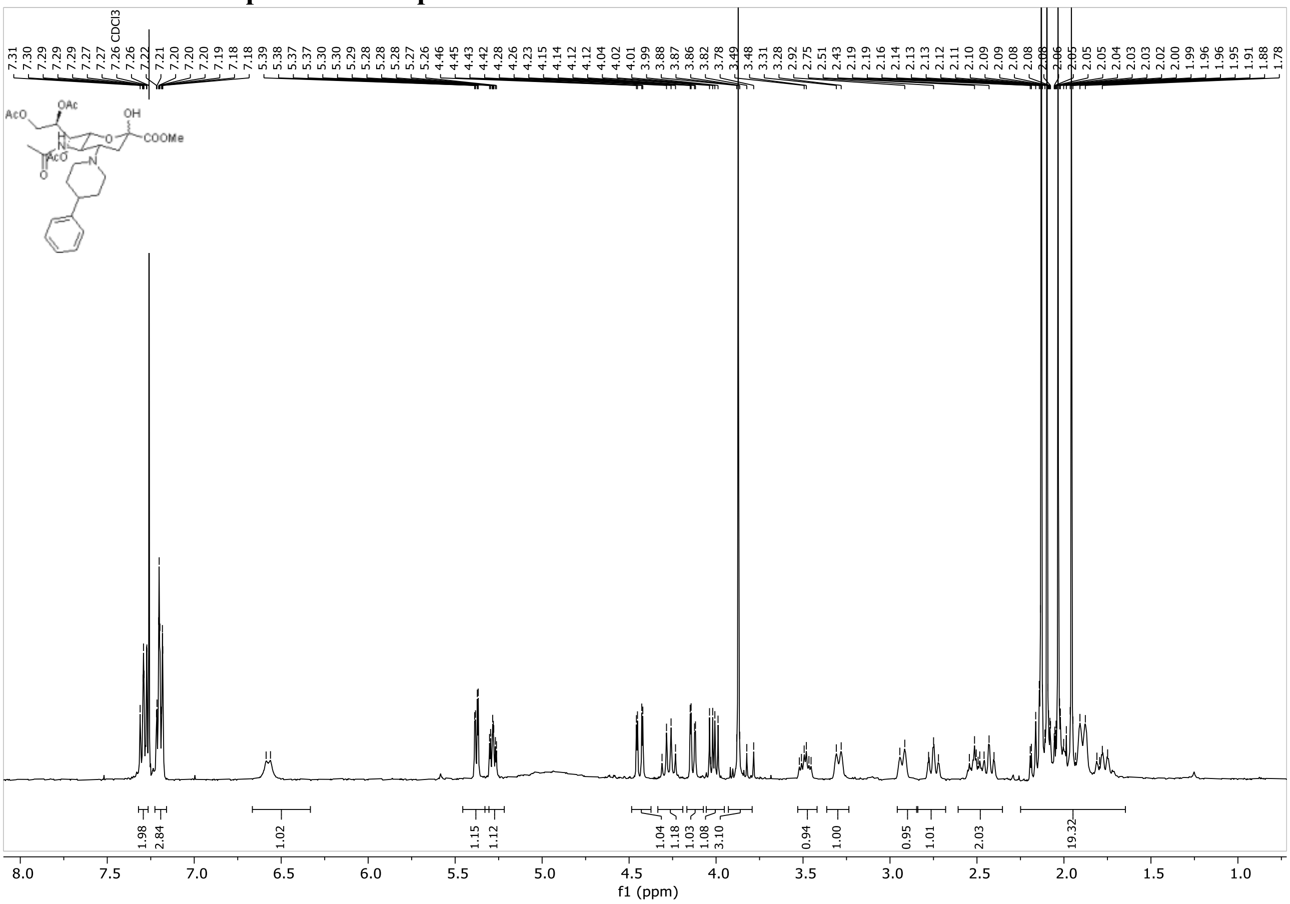

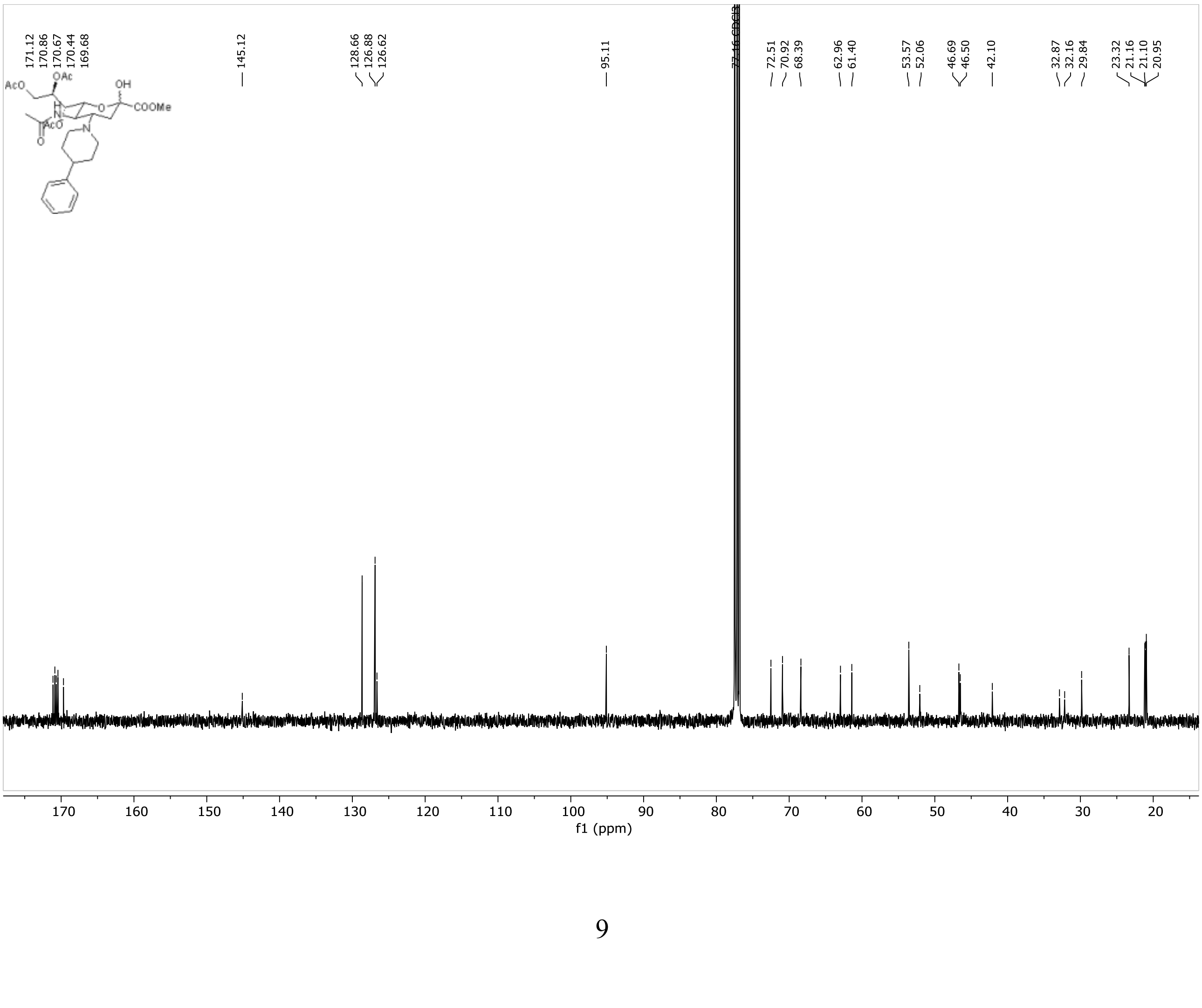

^{1}H- and ^{13}C-NMR spectra of compound 2f

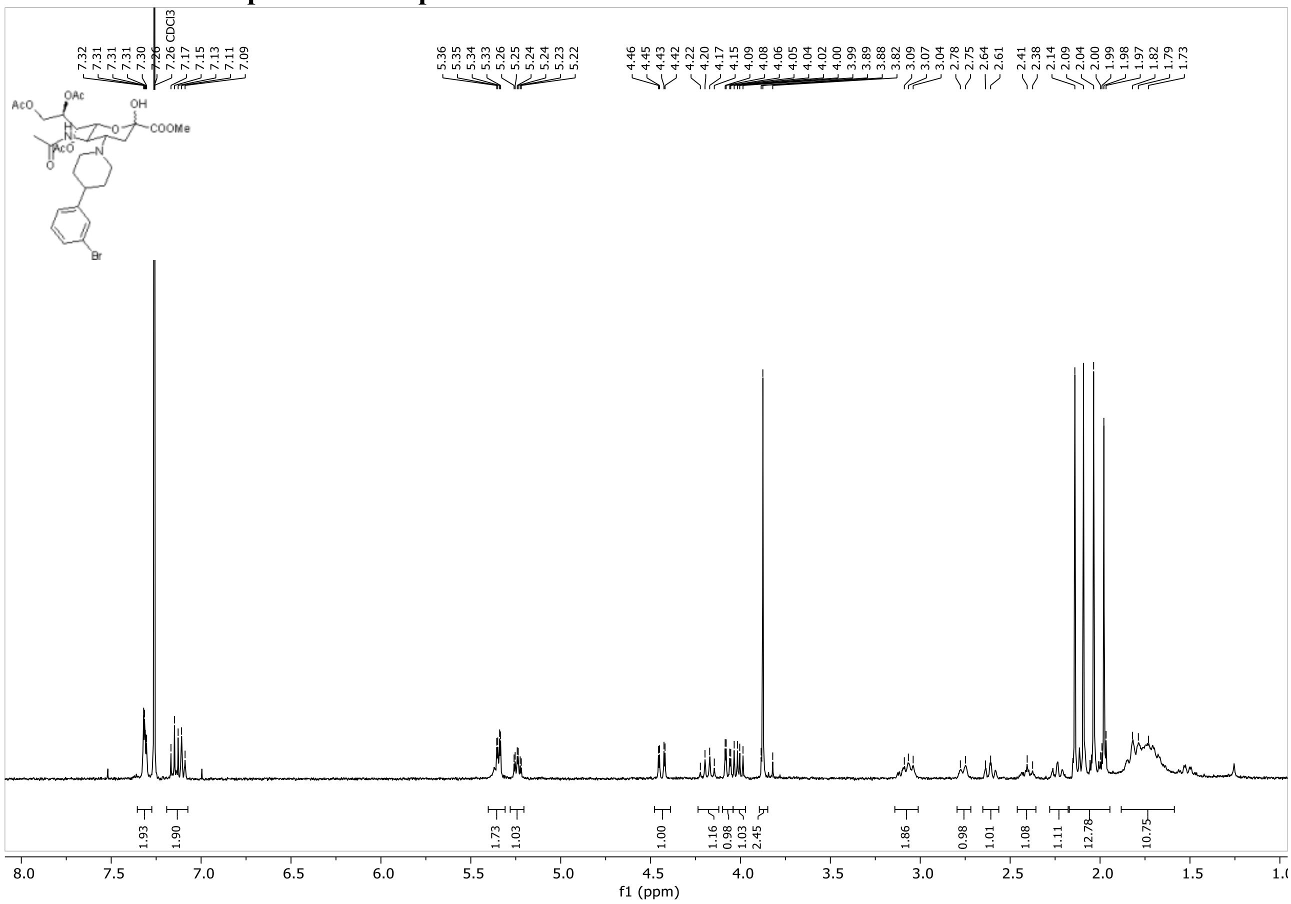

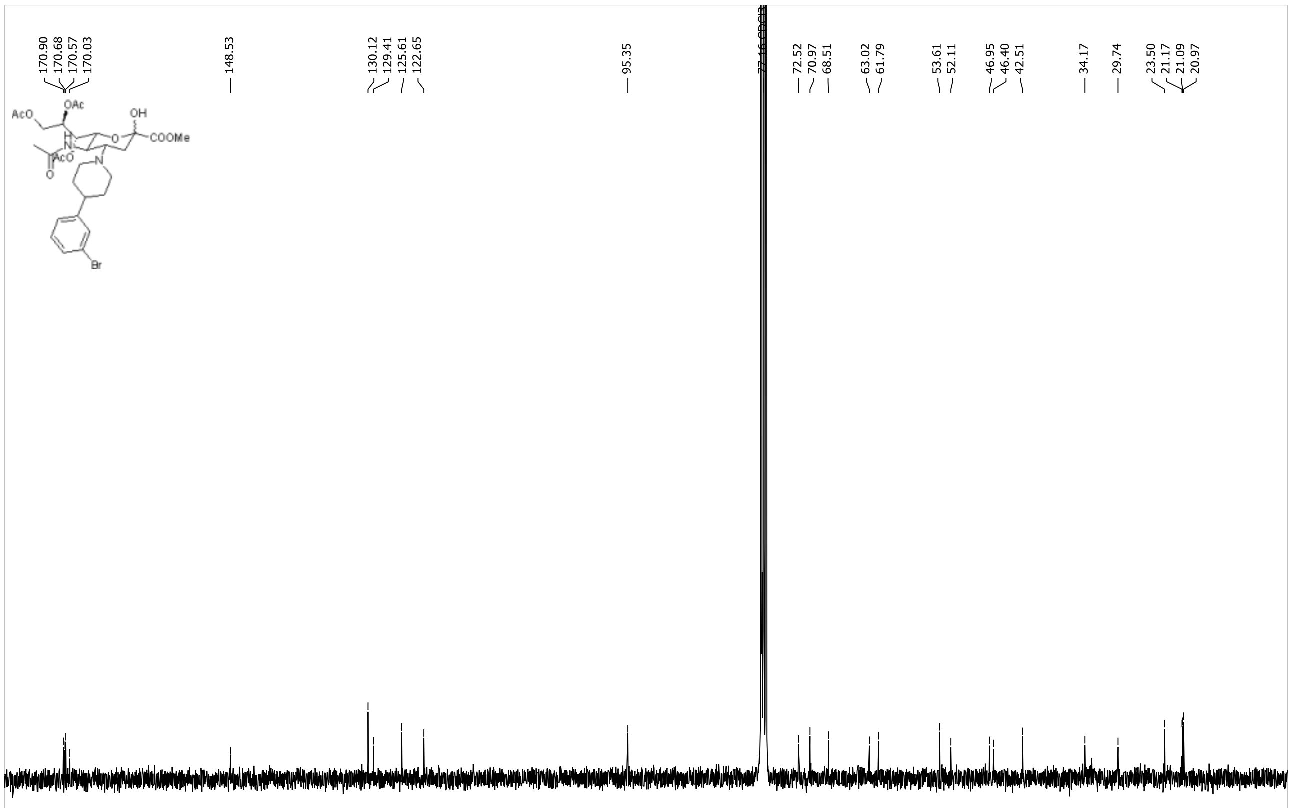

^{1}H- and ^{13}C-NMR spectra of compound 2g

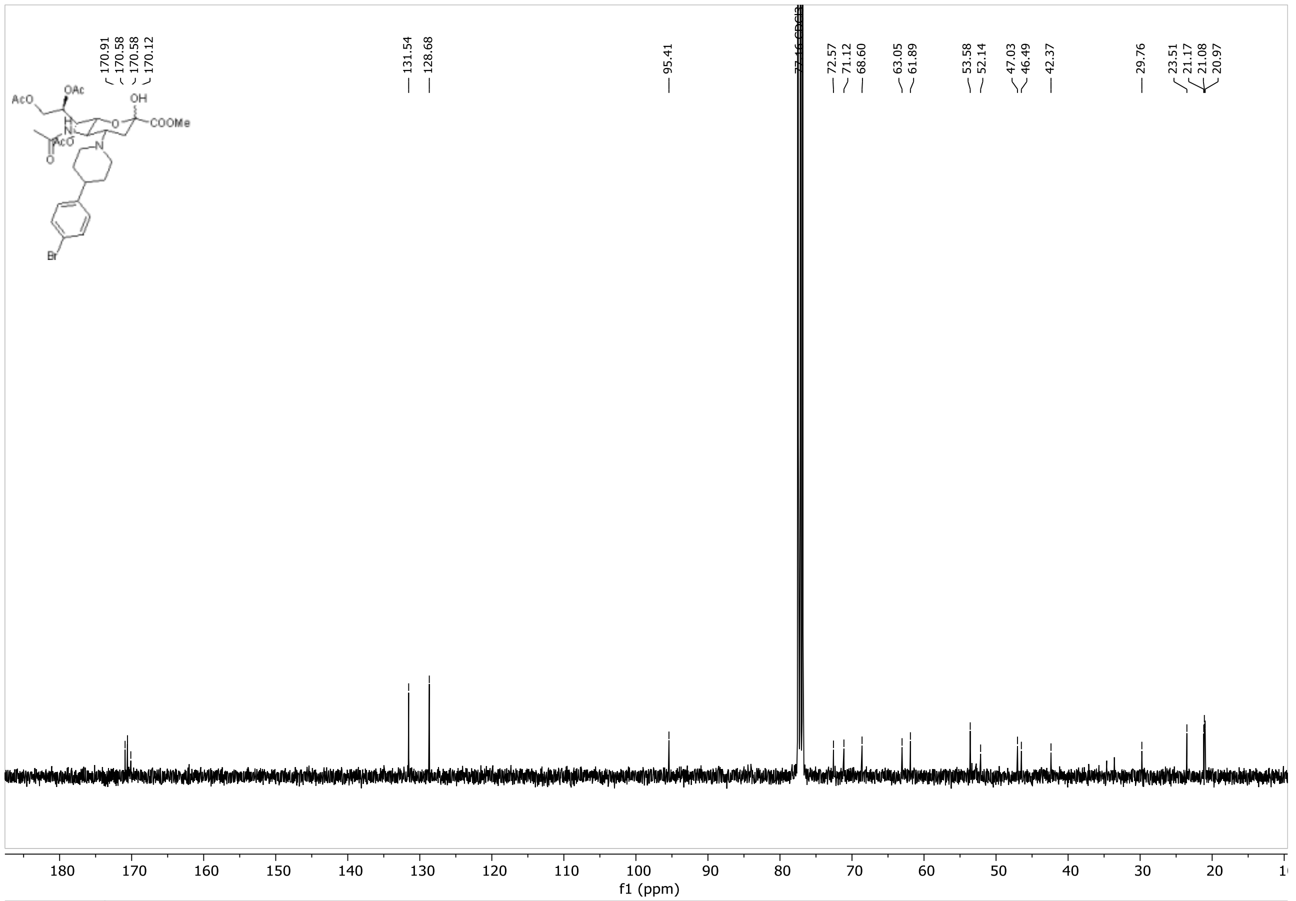

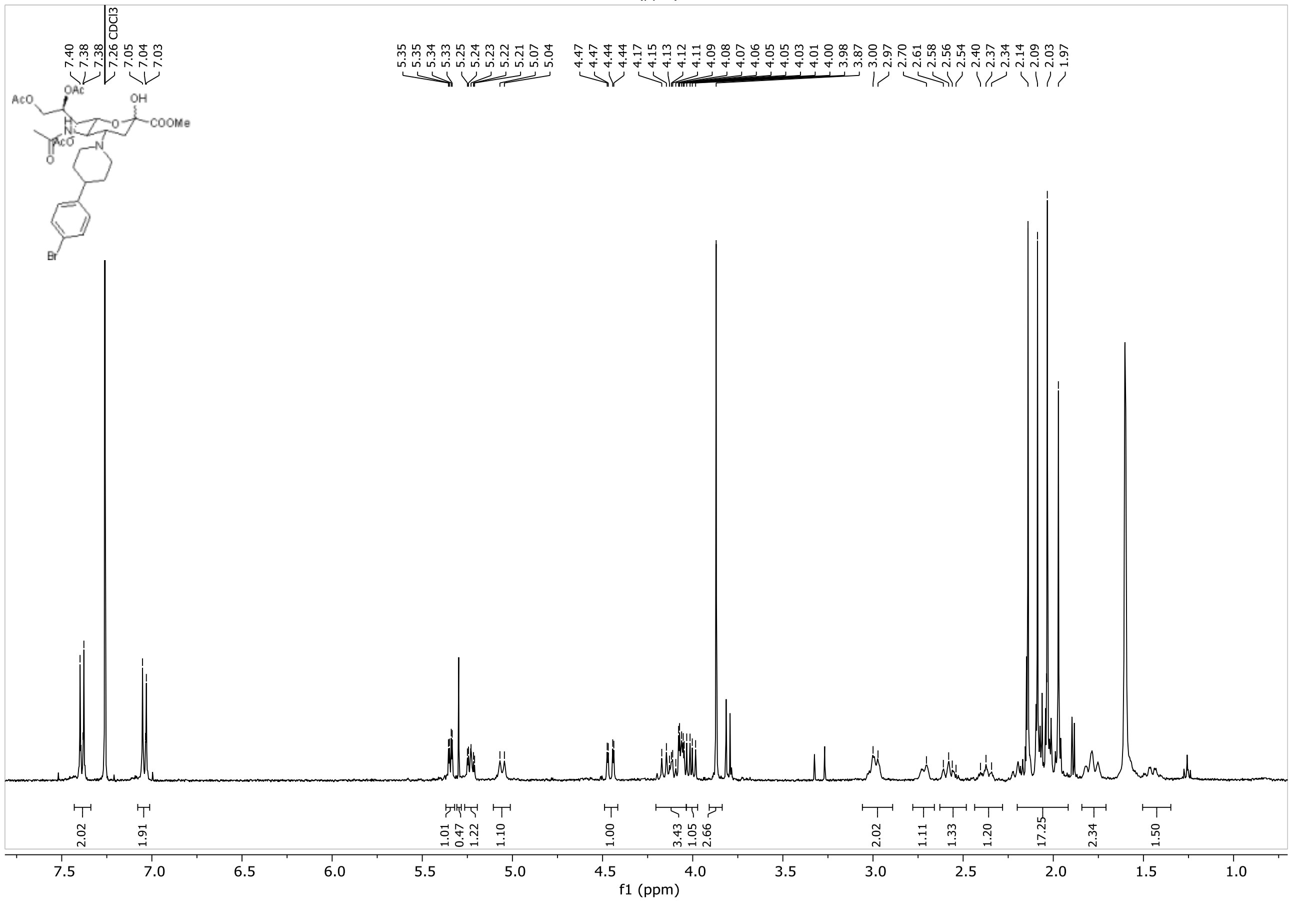

^{1}H- and ^{13}C-NMR spectra of compound 2h

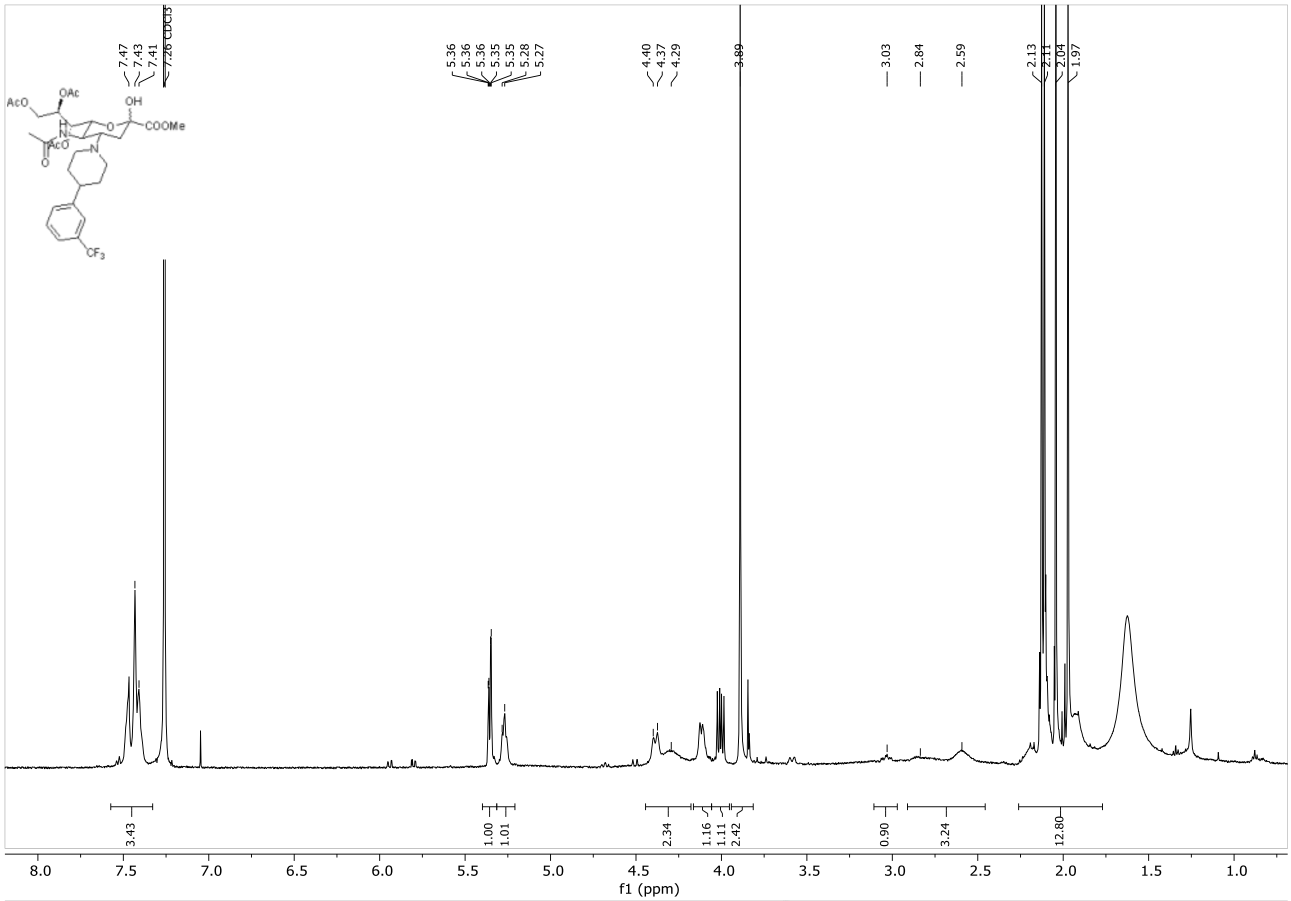

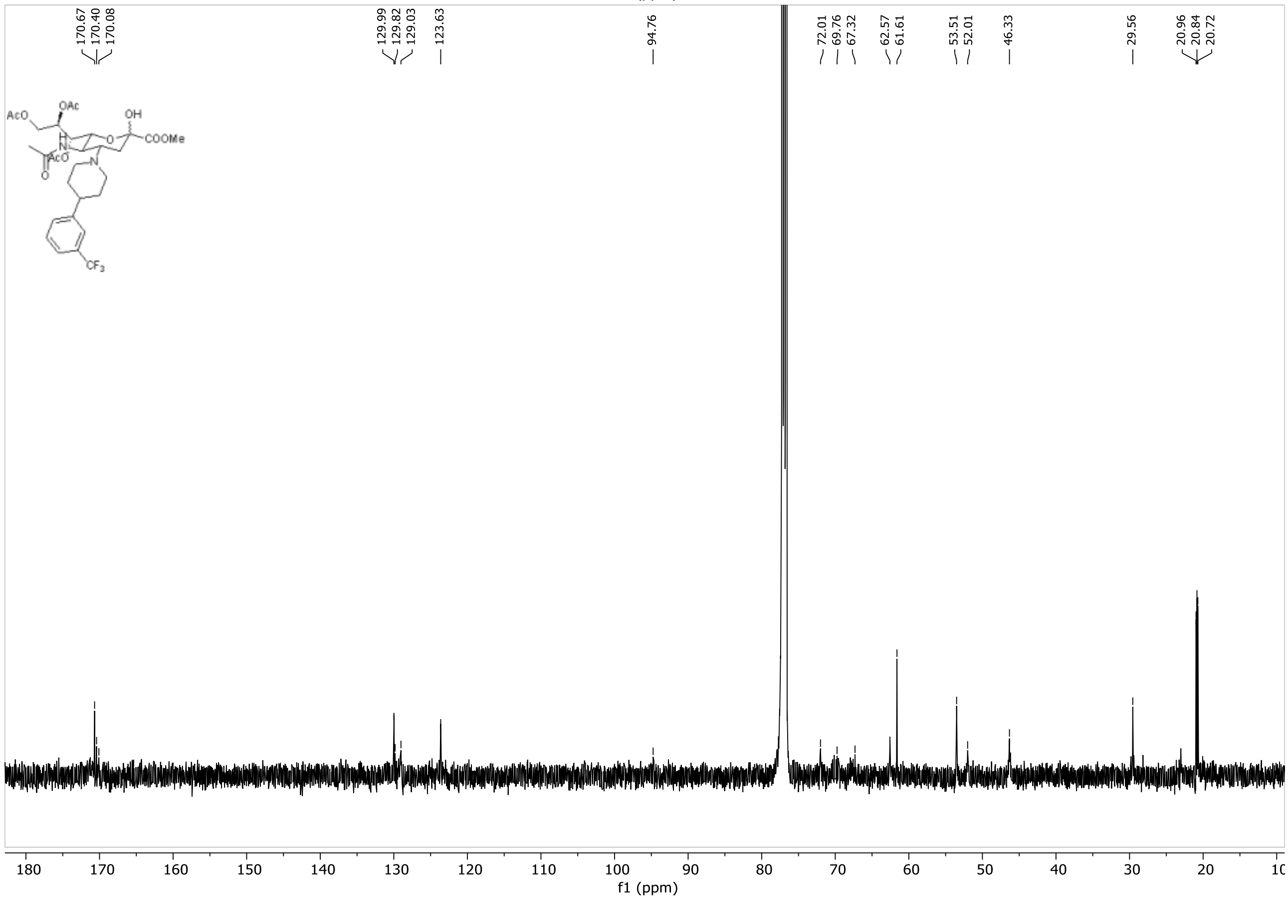

^{1}H- and ^{13}C-NMR spectra of compound 2i

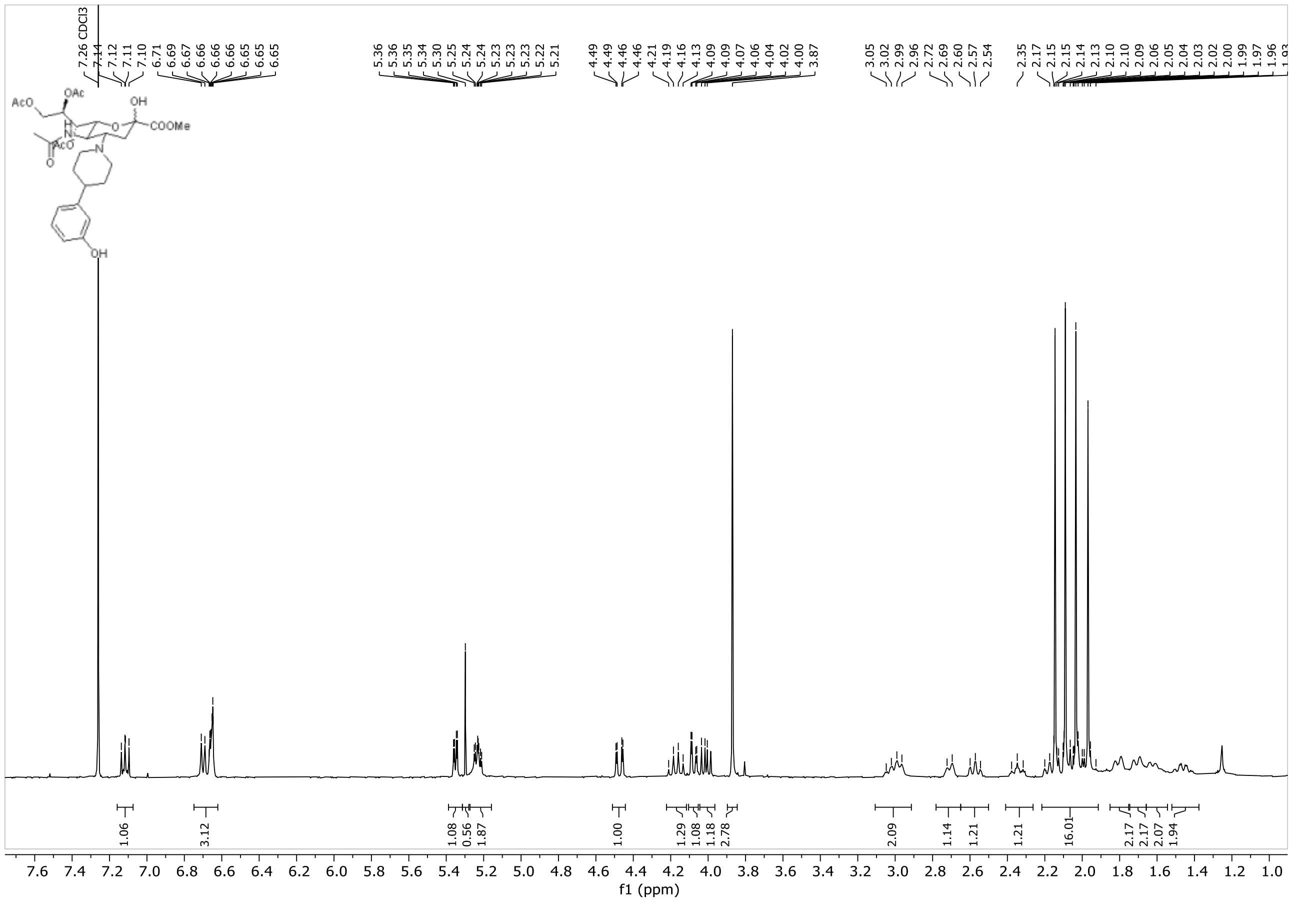

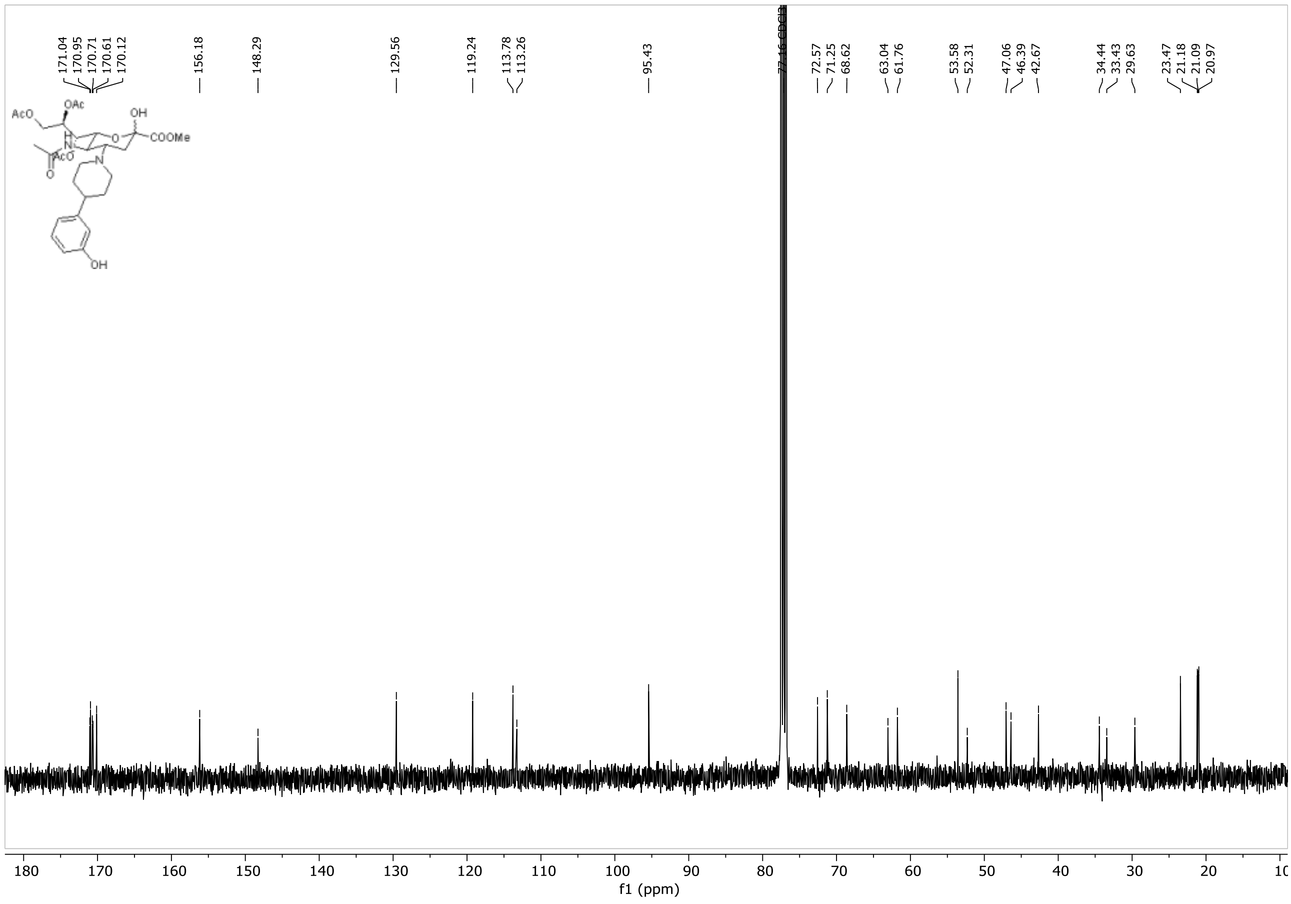

^{1}H- and ^{13}C-NMR spectra of compound 5a

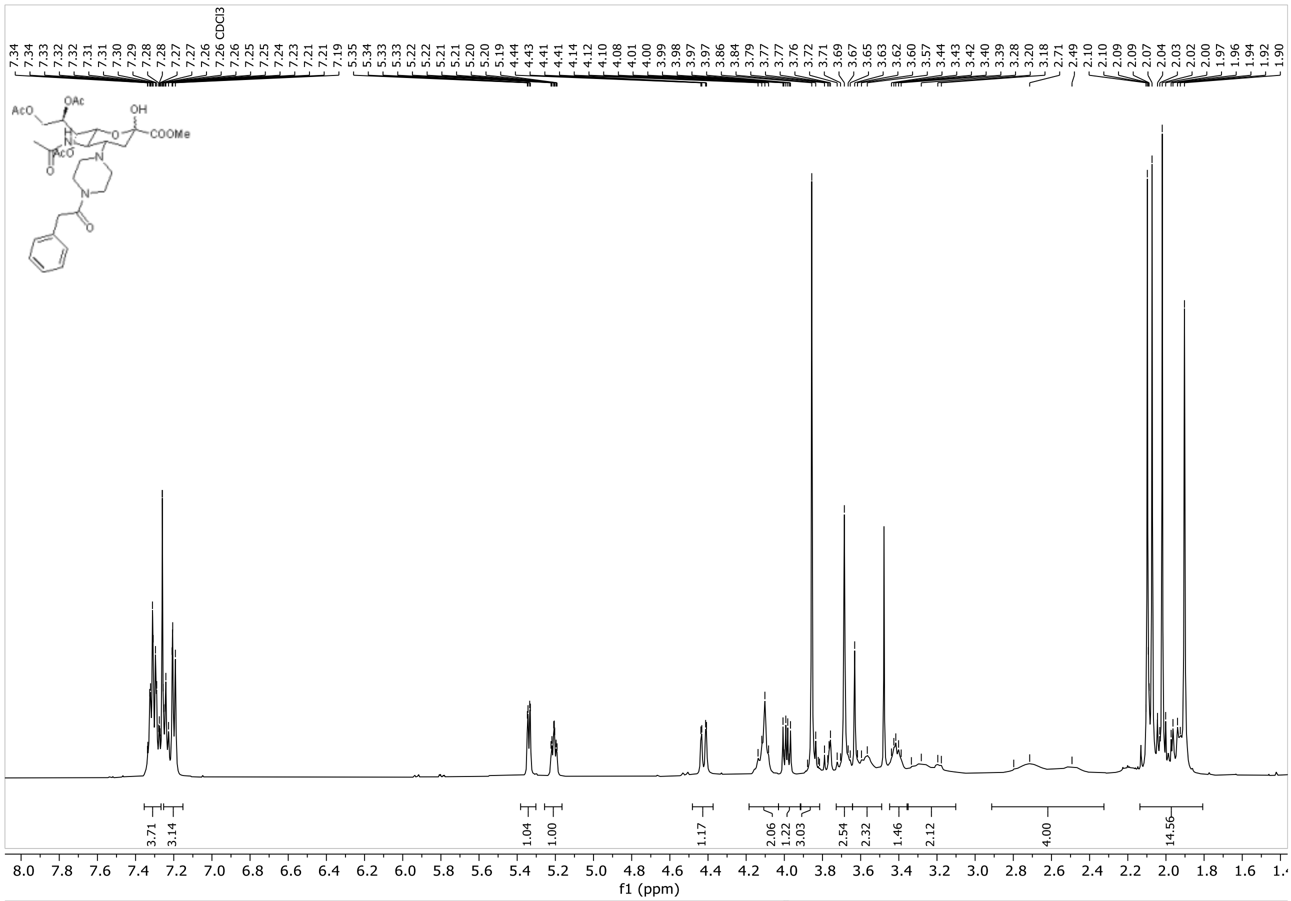

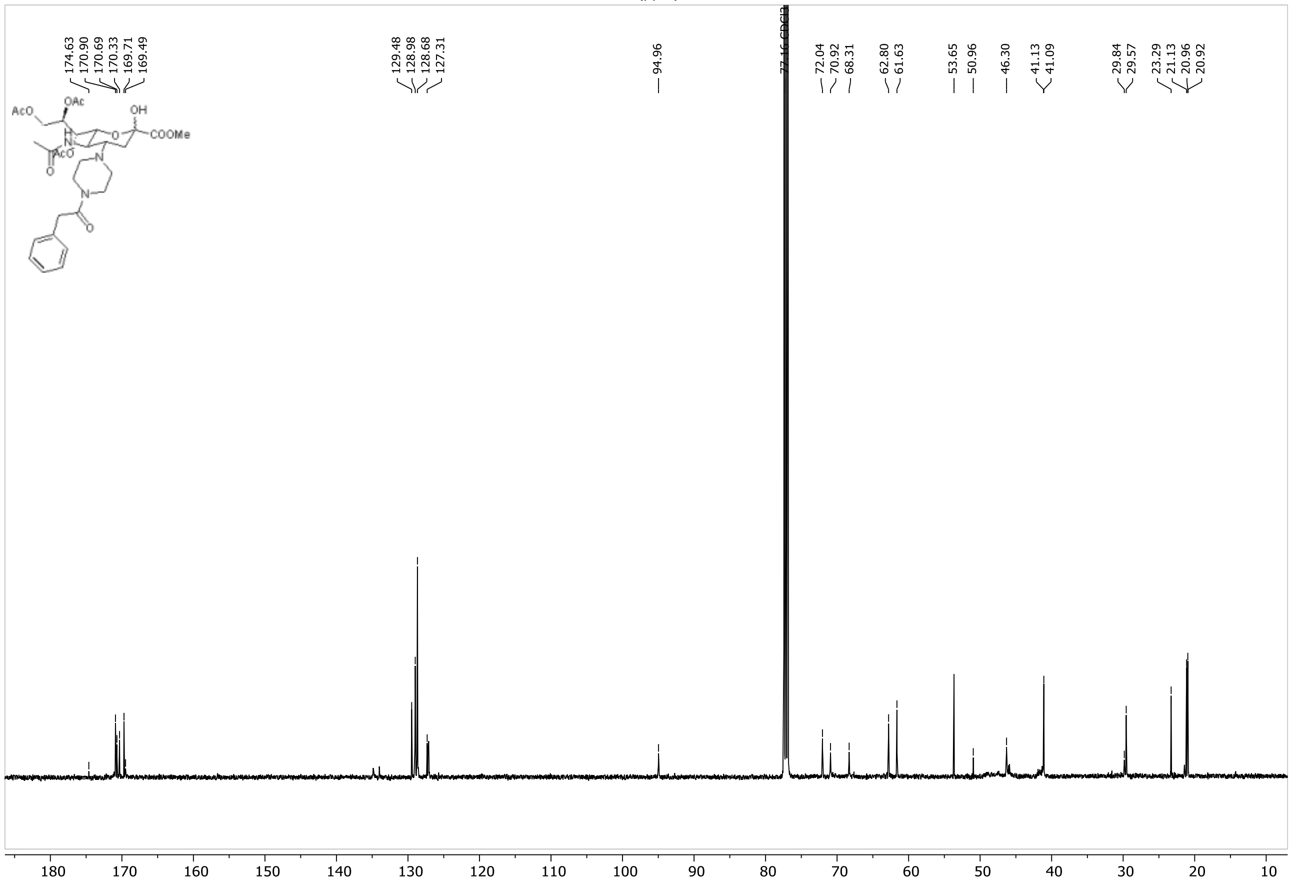

^{1}H- and ^{13}C-NMR spectra of compound 5b

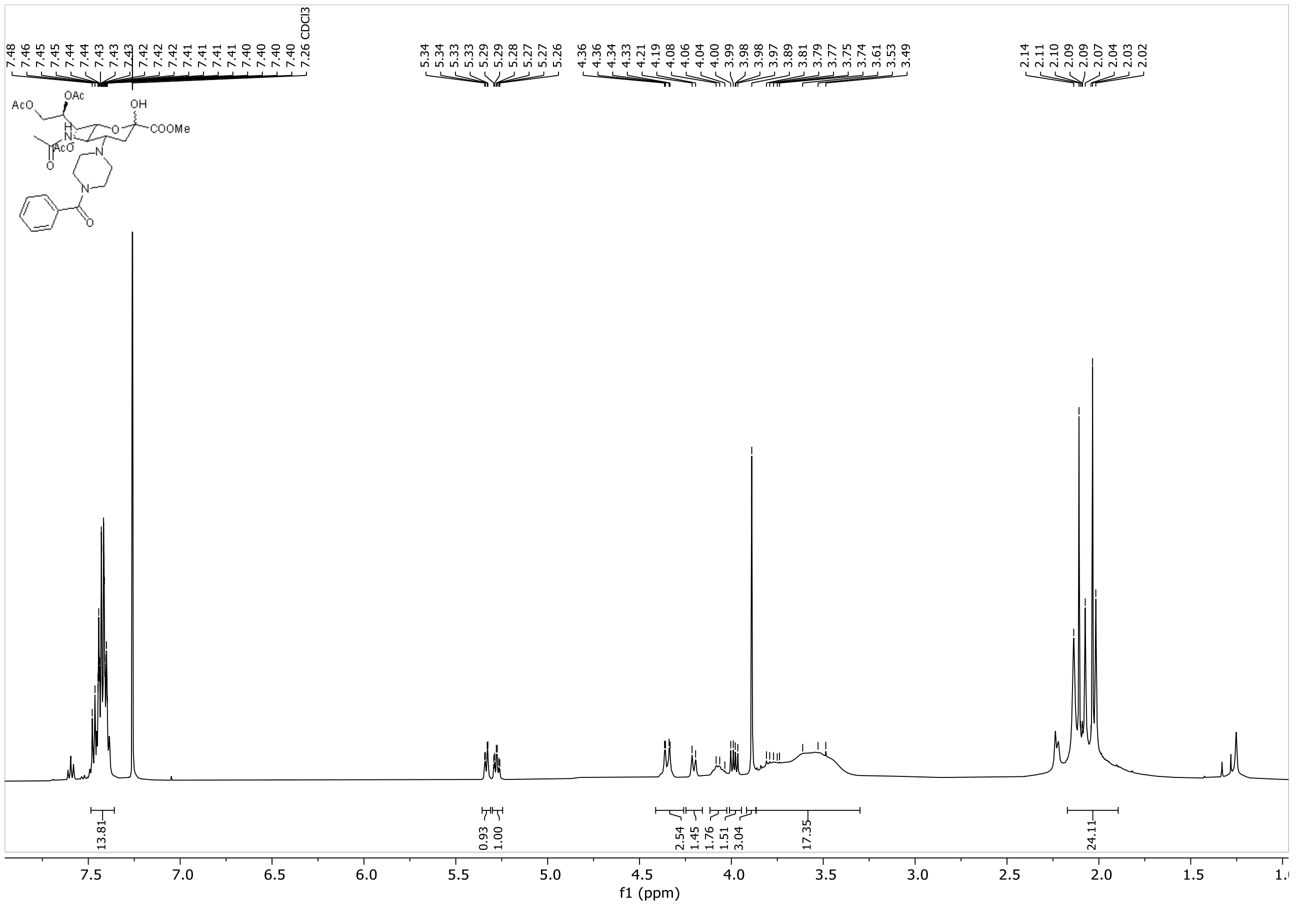

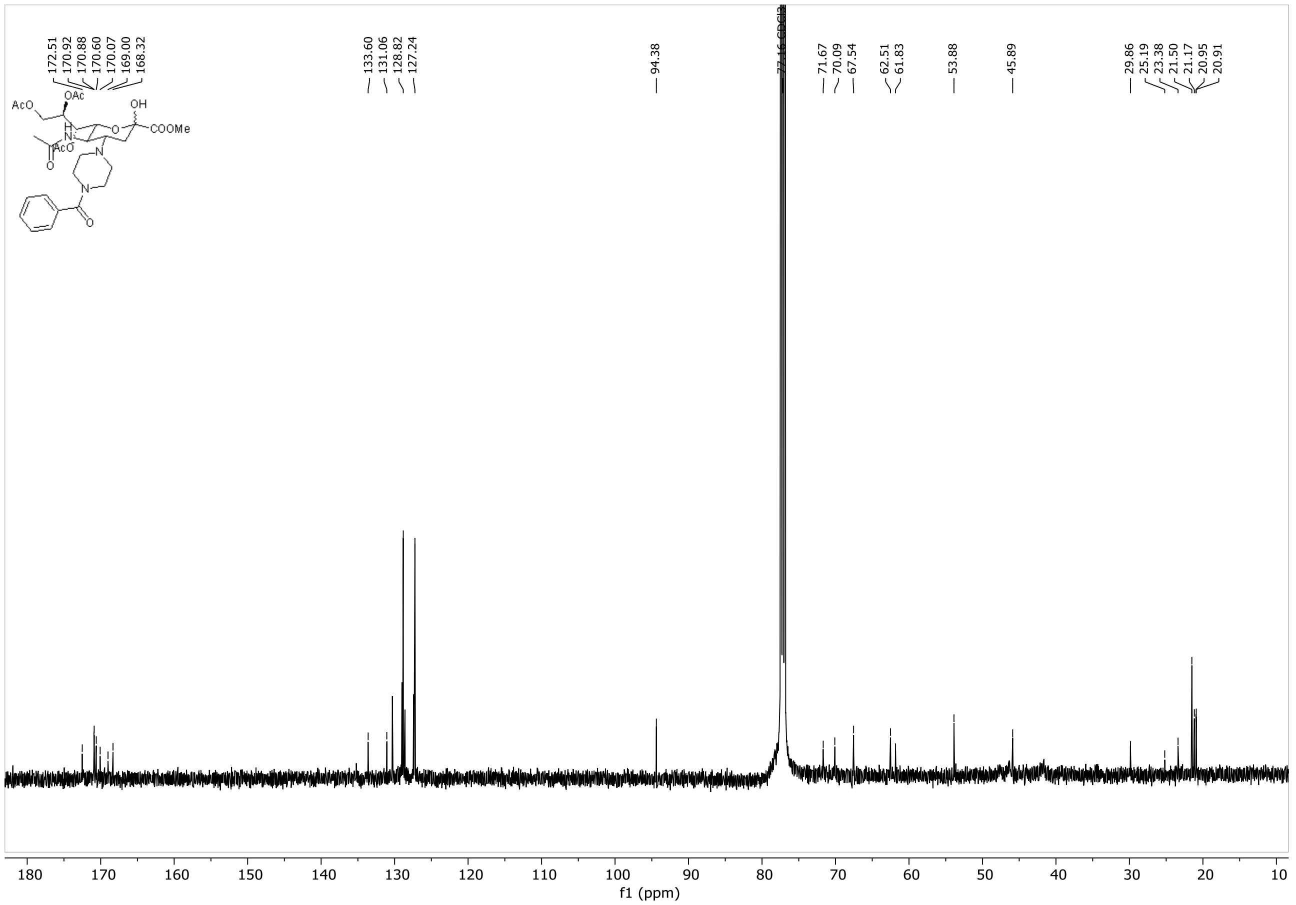

^{1}H- and ^{13}C-NMR spectra of compound 5c

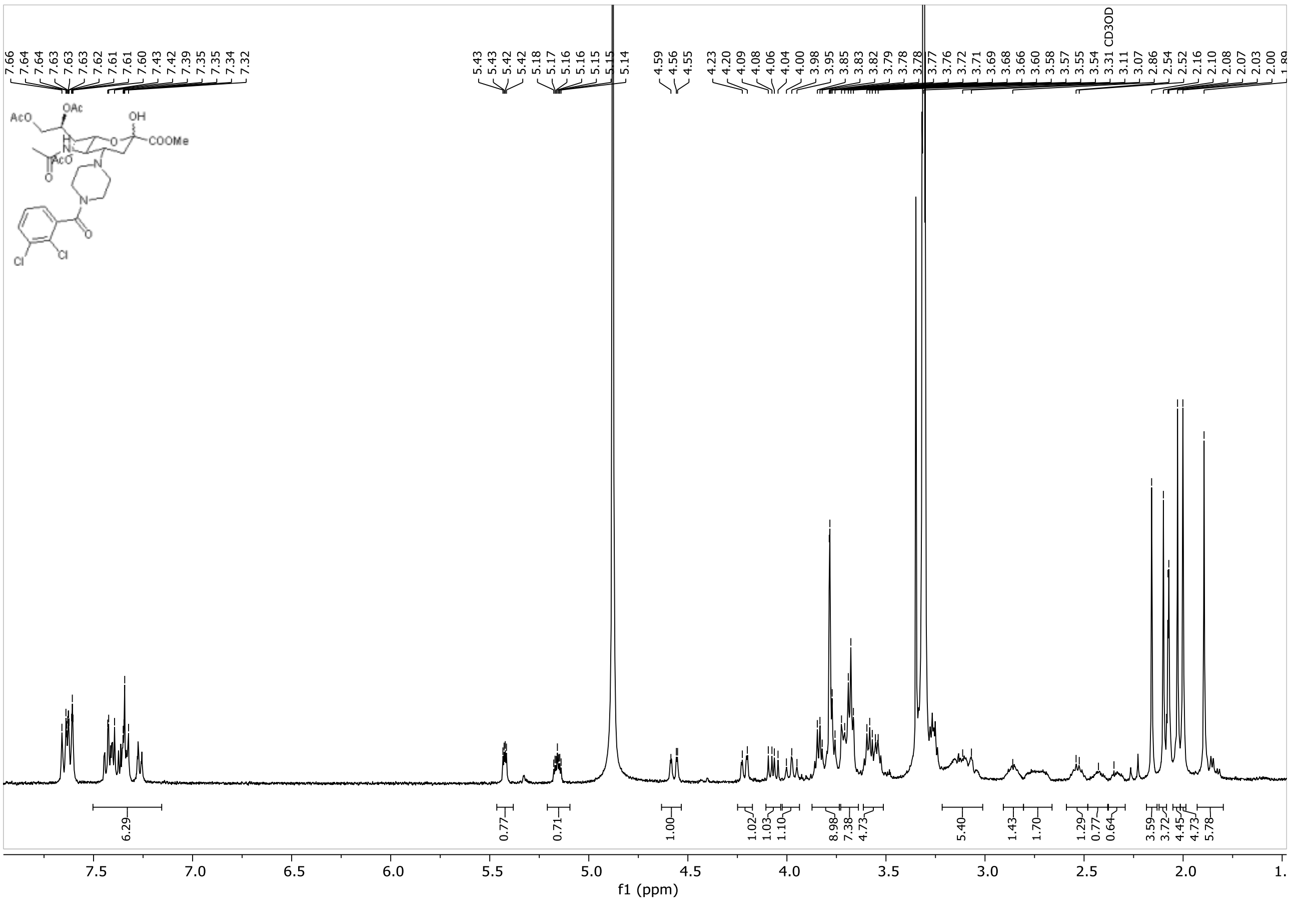

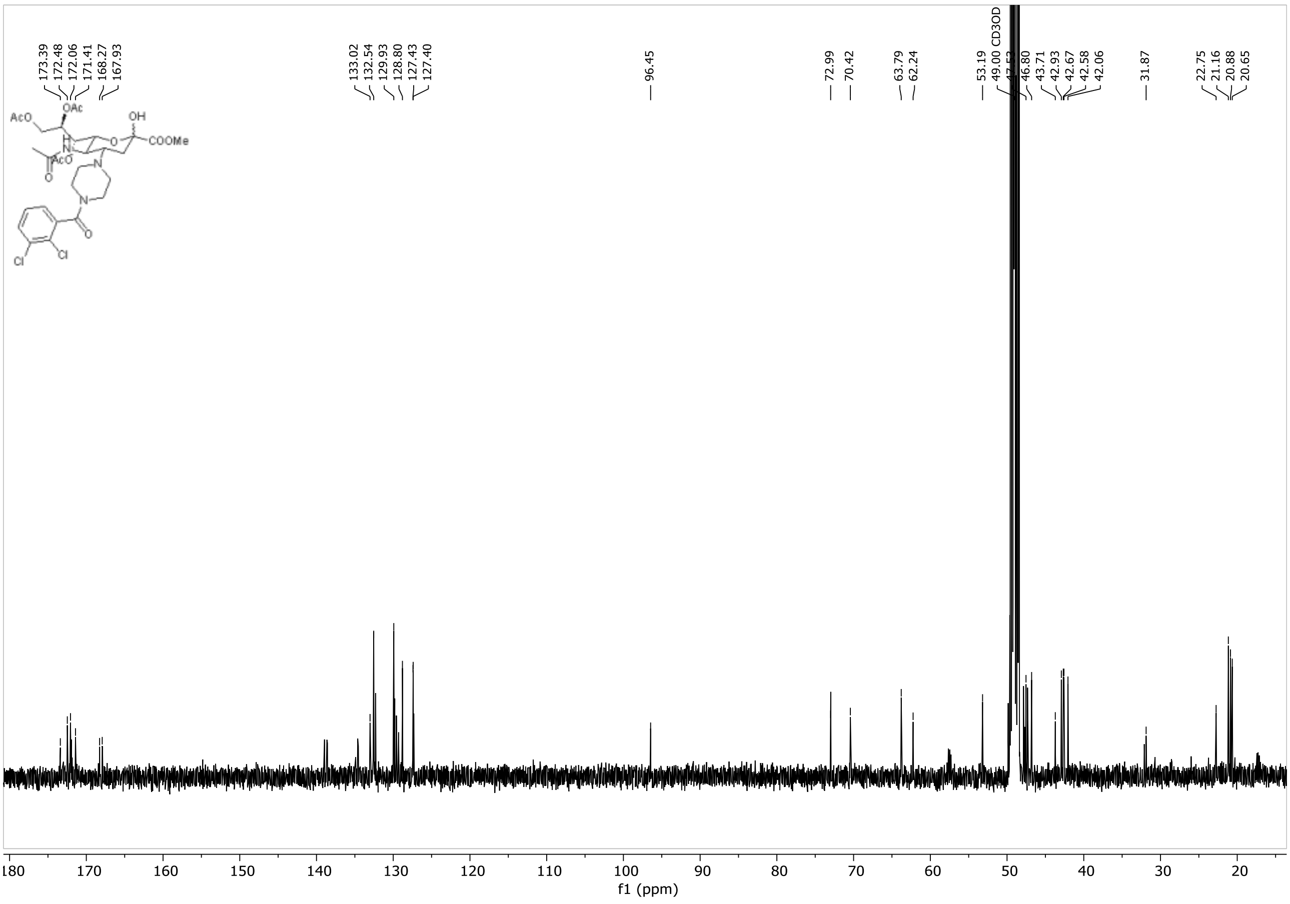

^{1}H- and ^{13}C-NMR spectra of compound 5d

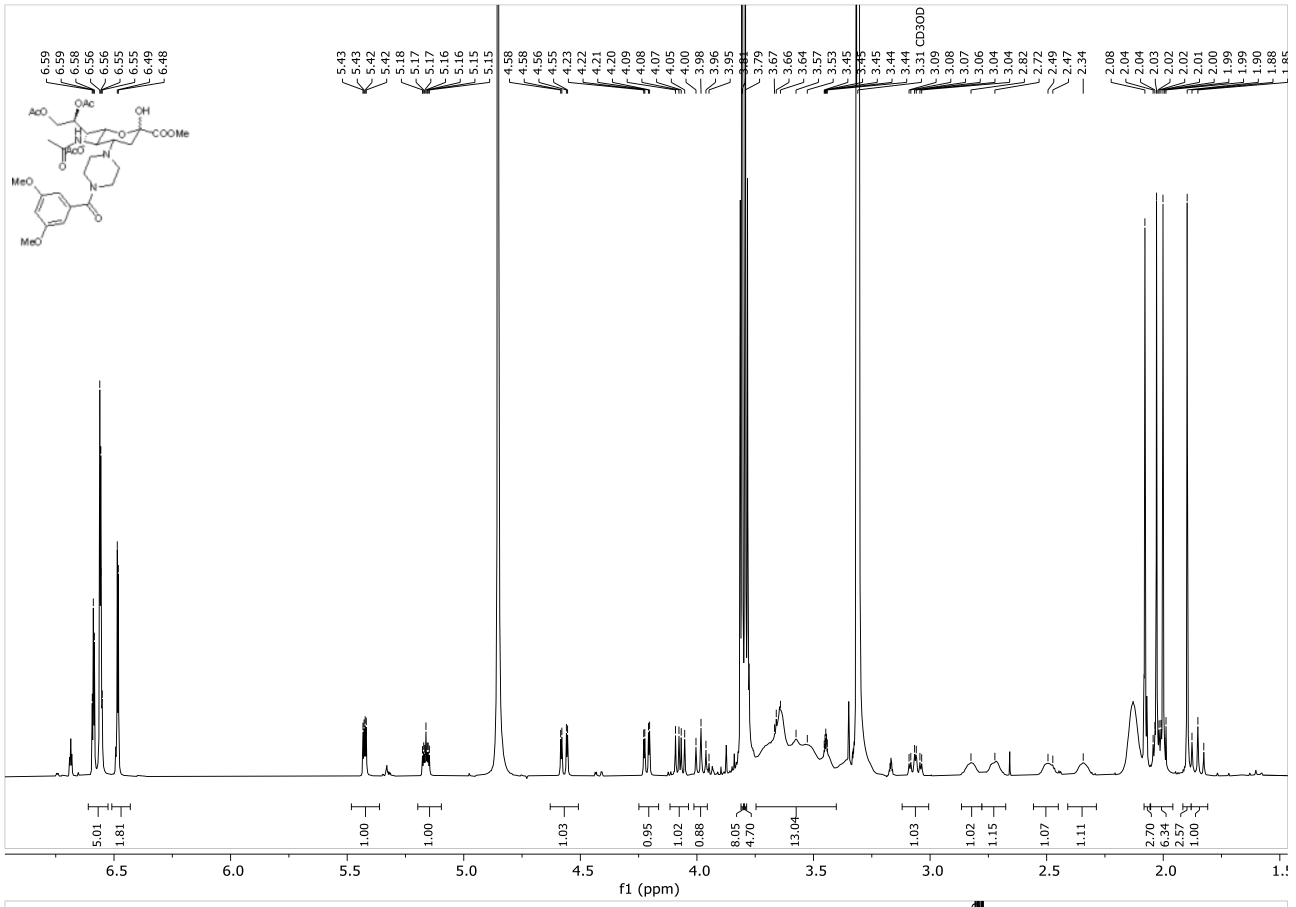

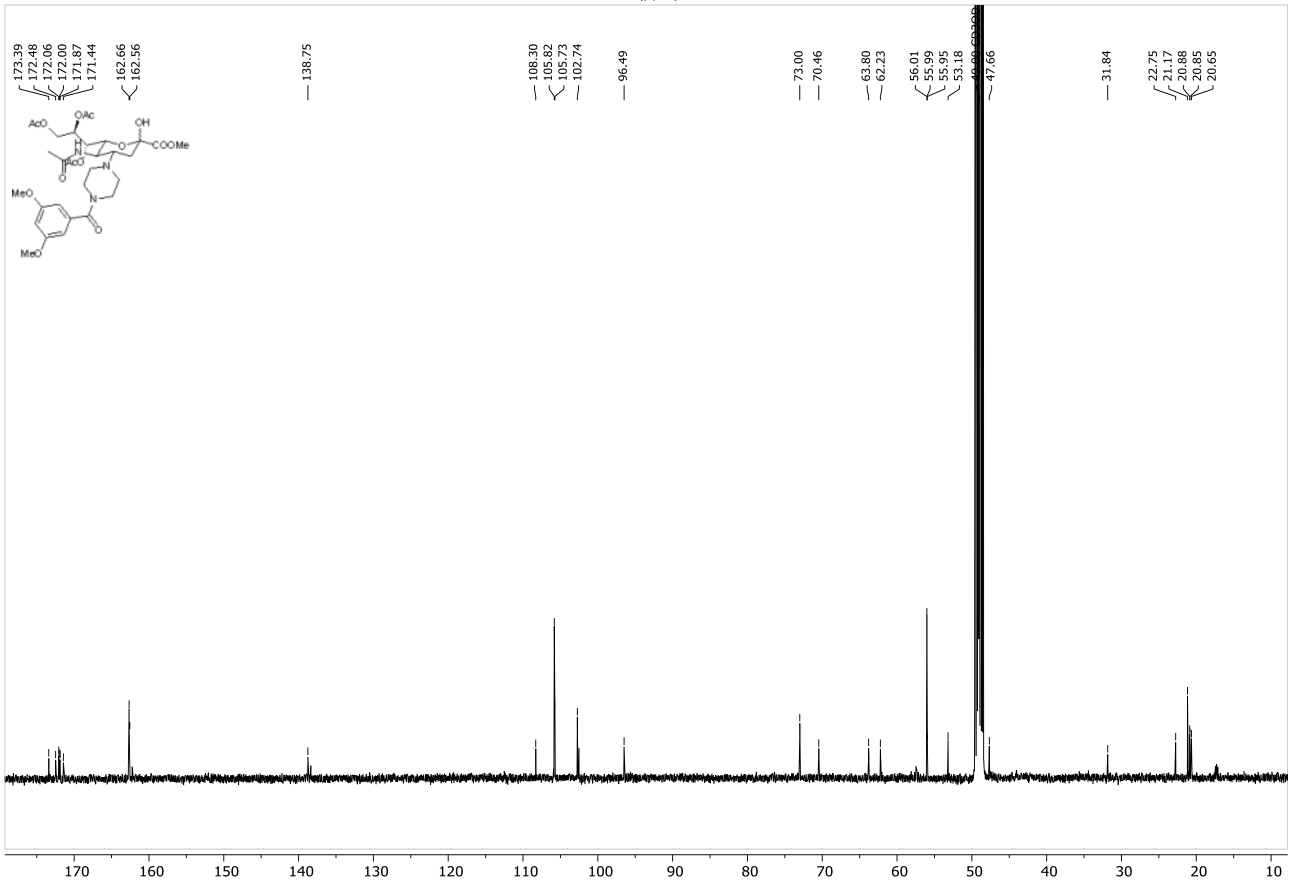

^{1}H- and ^{13}C-NMR spectra of compound 3a

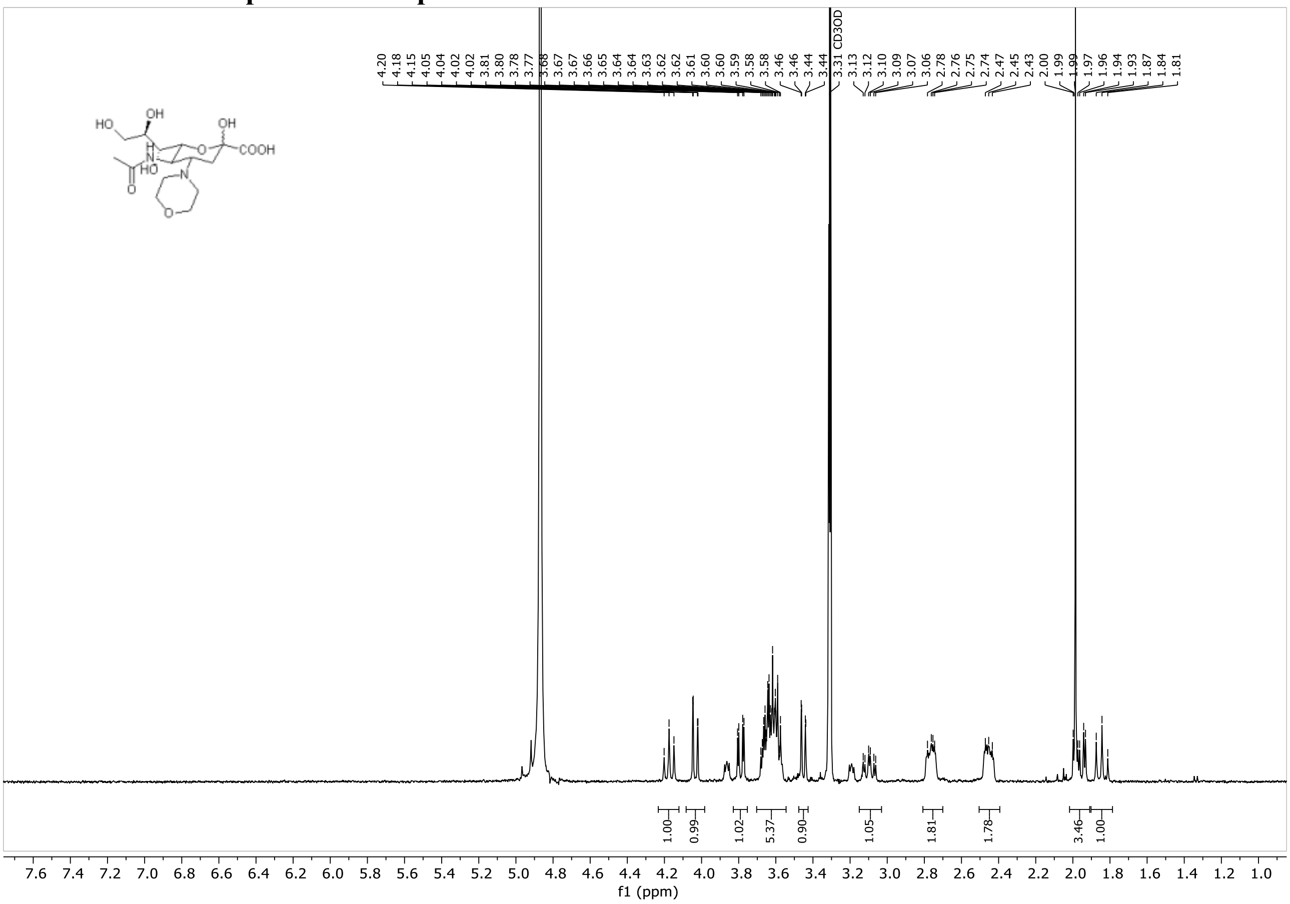

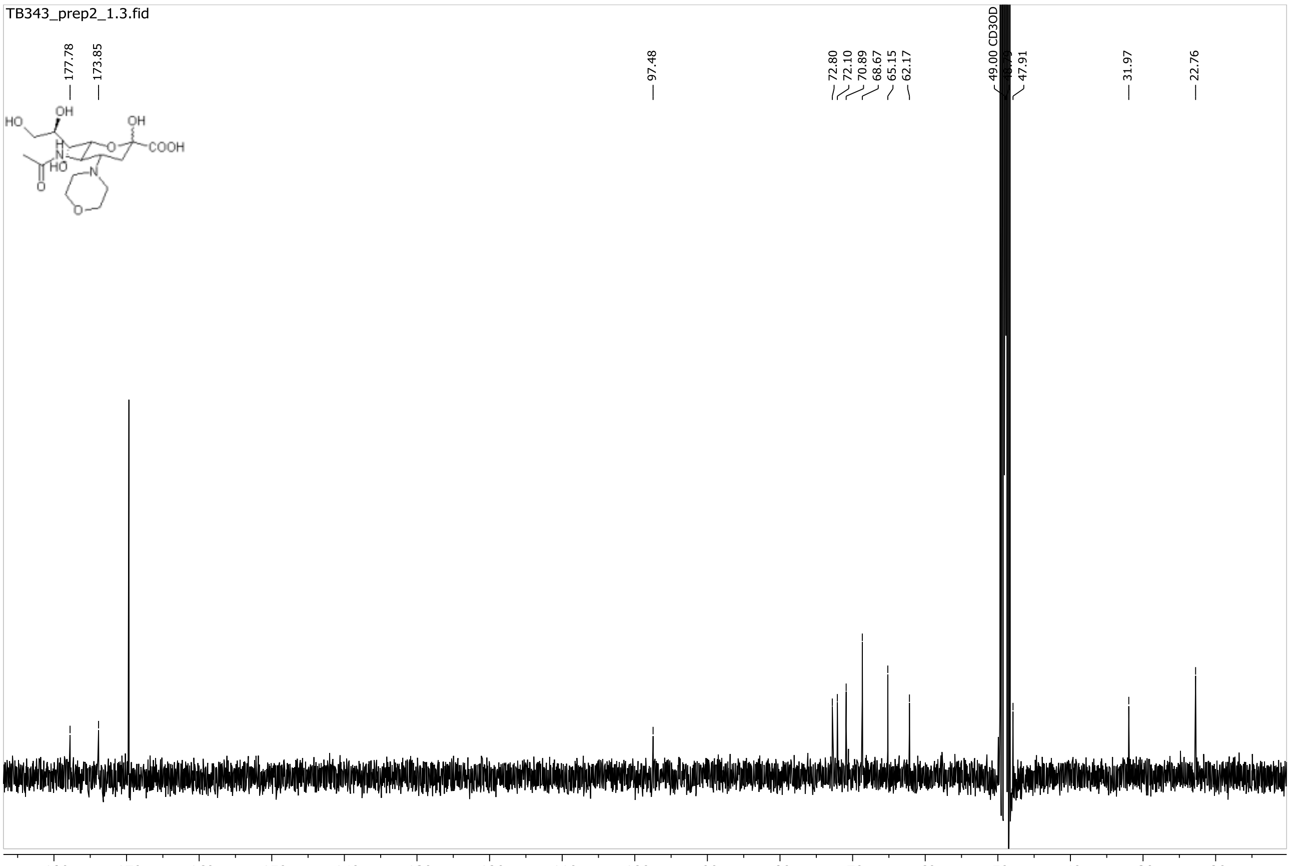

^{1}H- and ^{13}C-NMR spectra of compound 3b

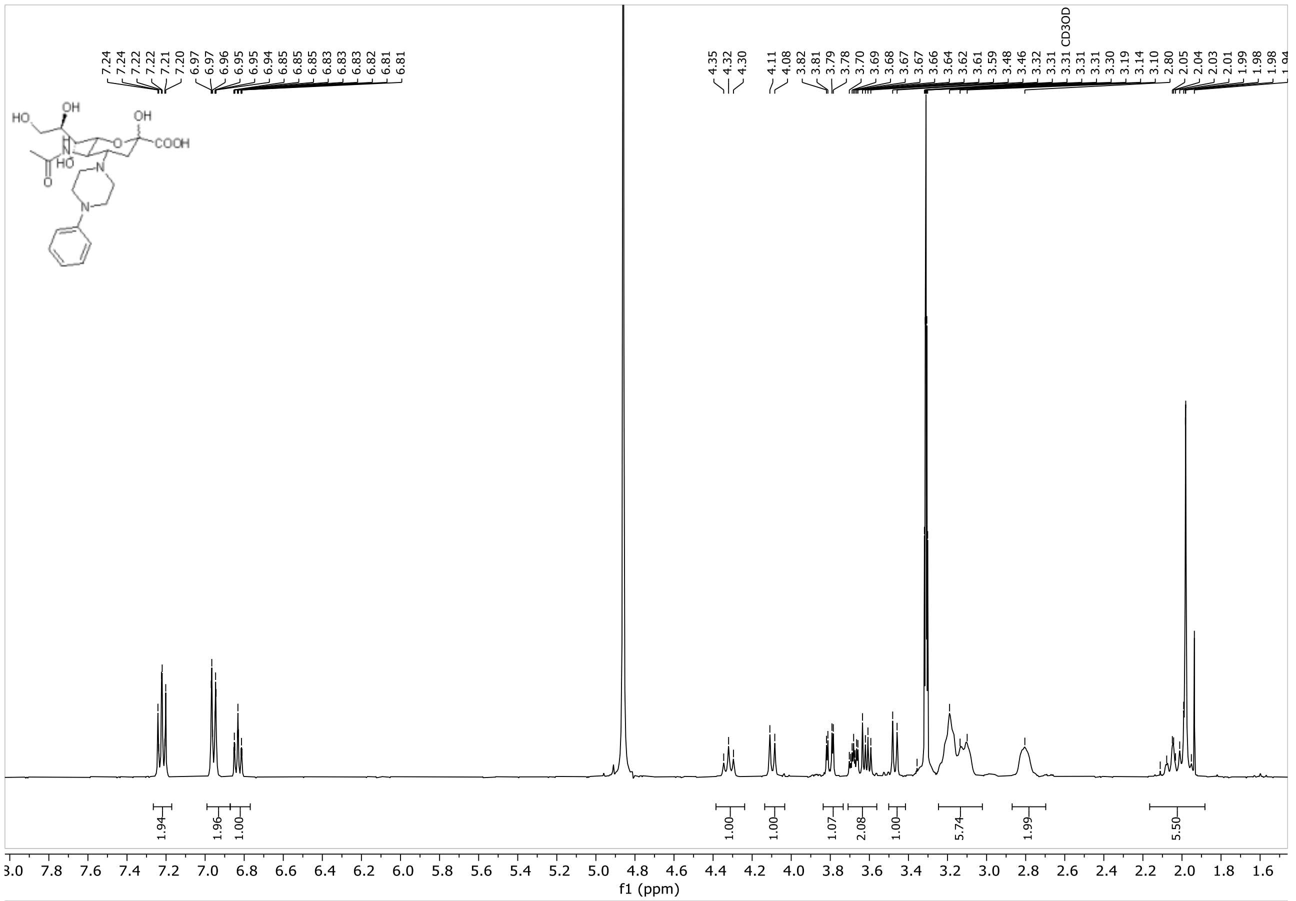

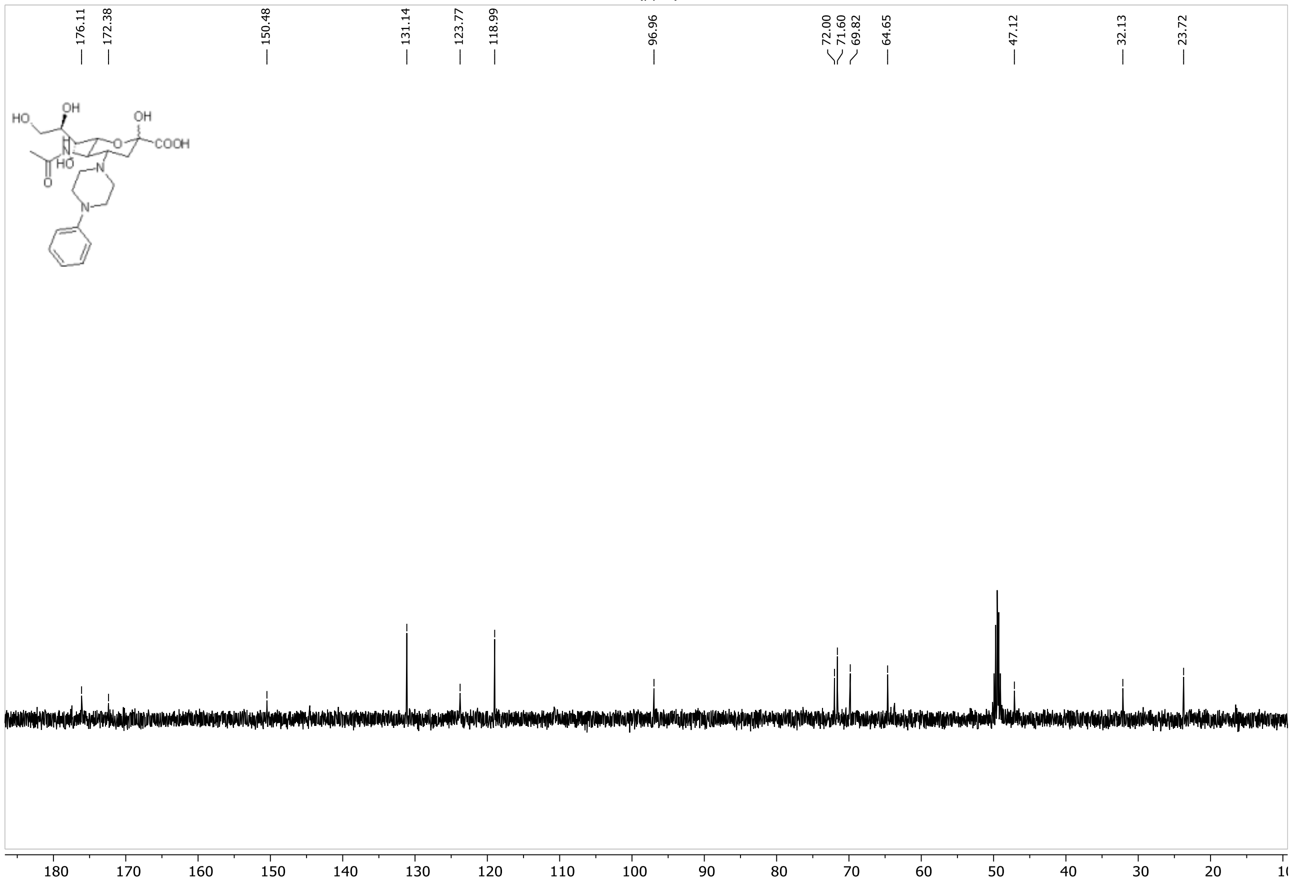

^{1}H- and ^{13}C-NMR spectra of compound 3c

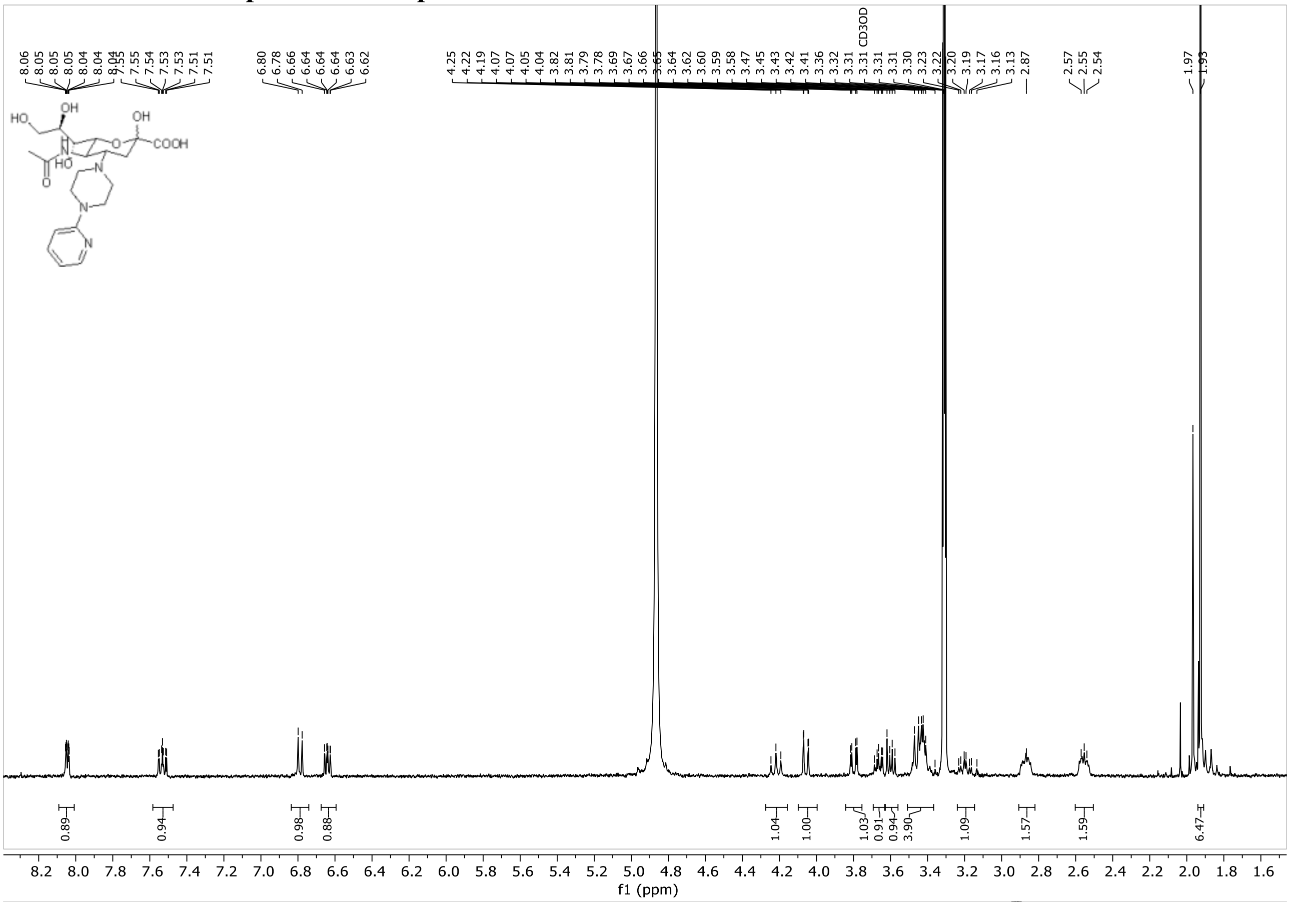

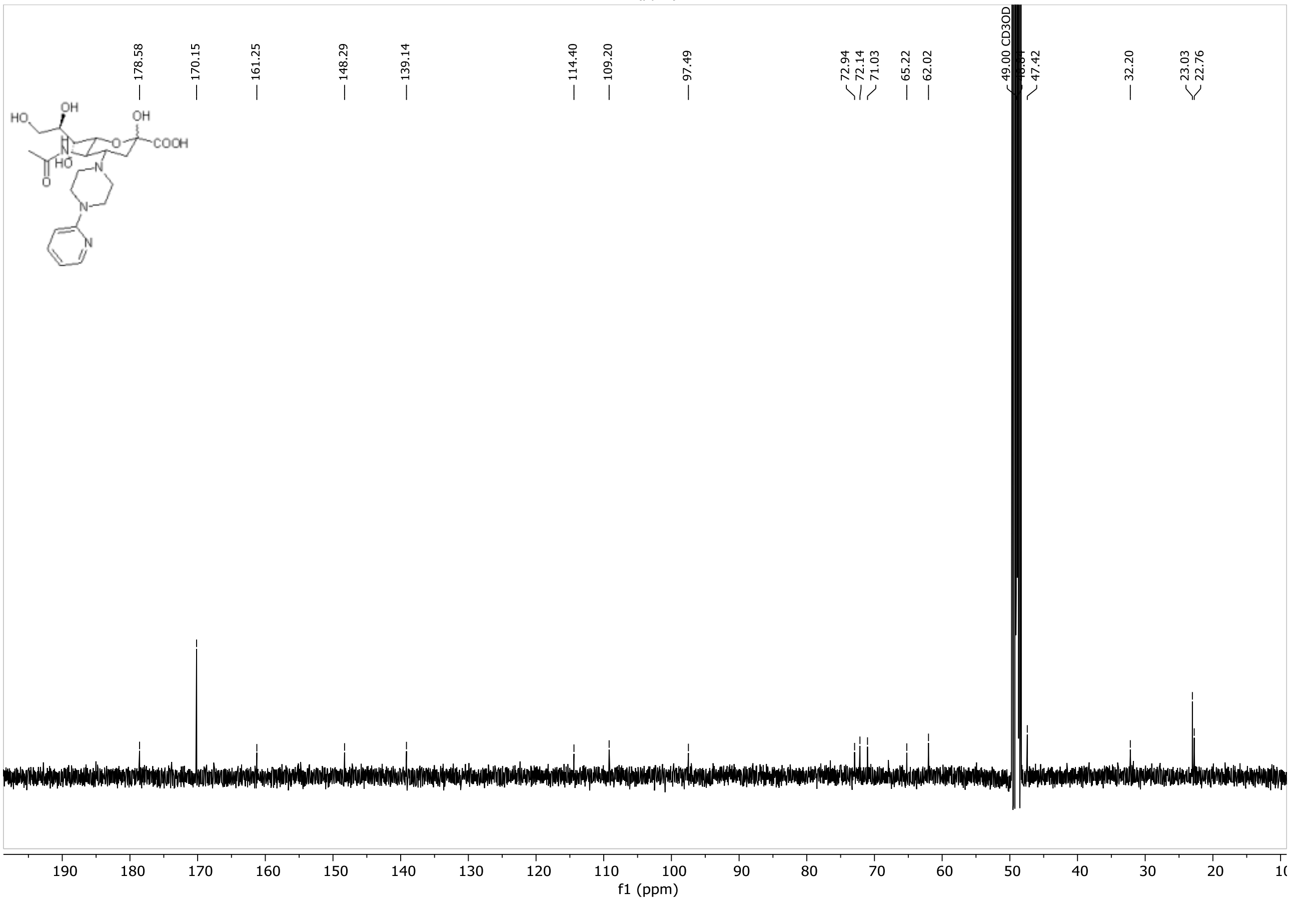

^{1}H- and ^{13}C-NMR spectra of compound 3d

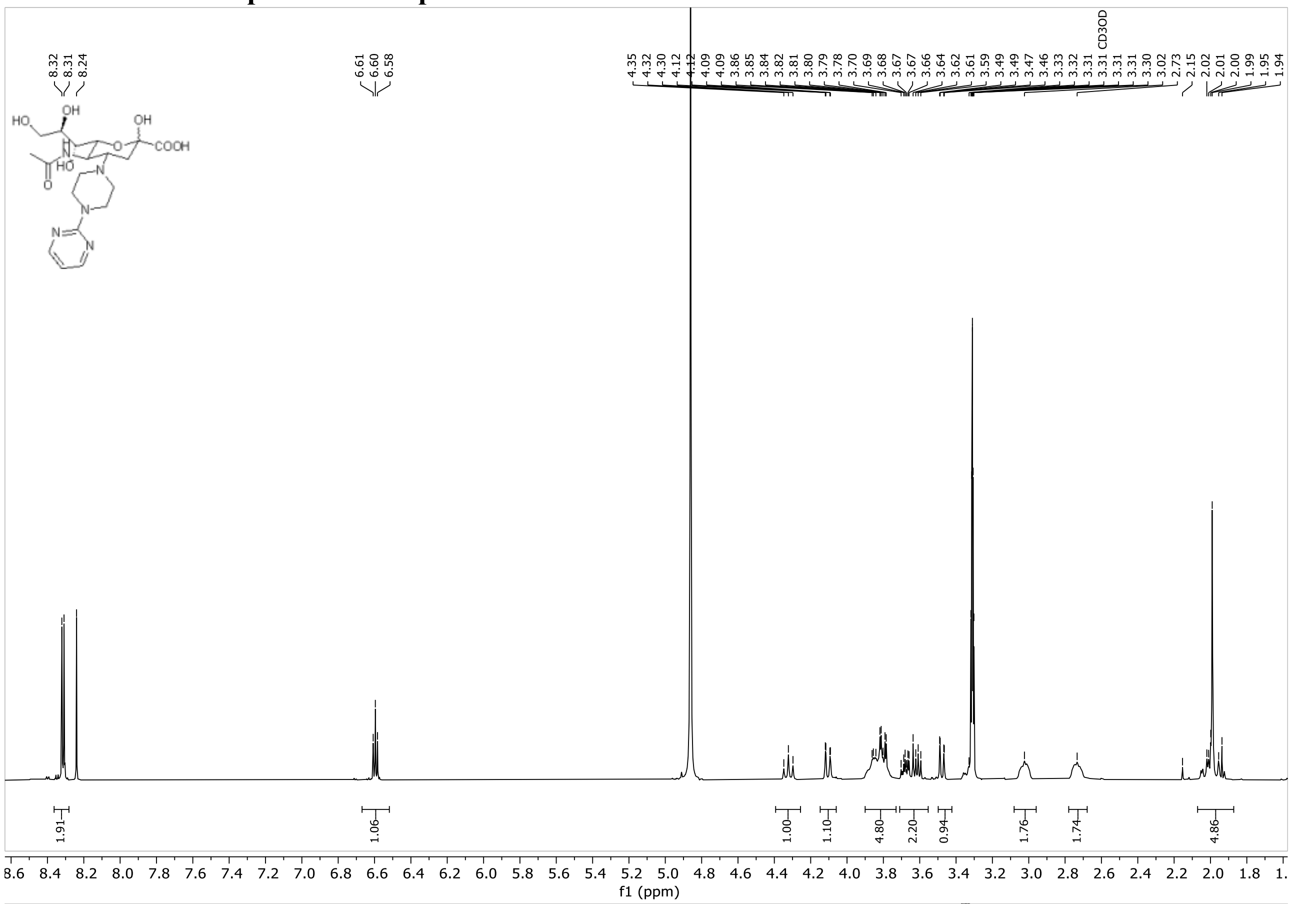

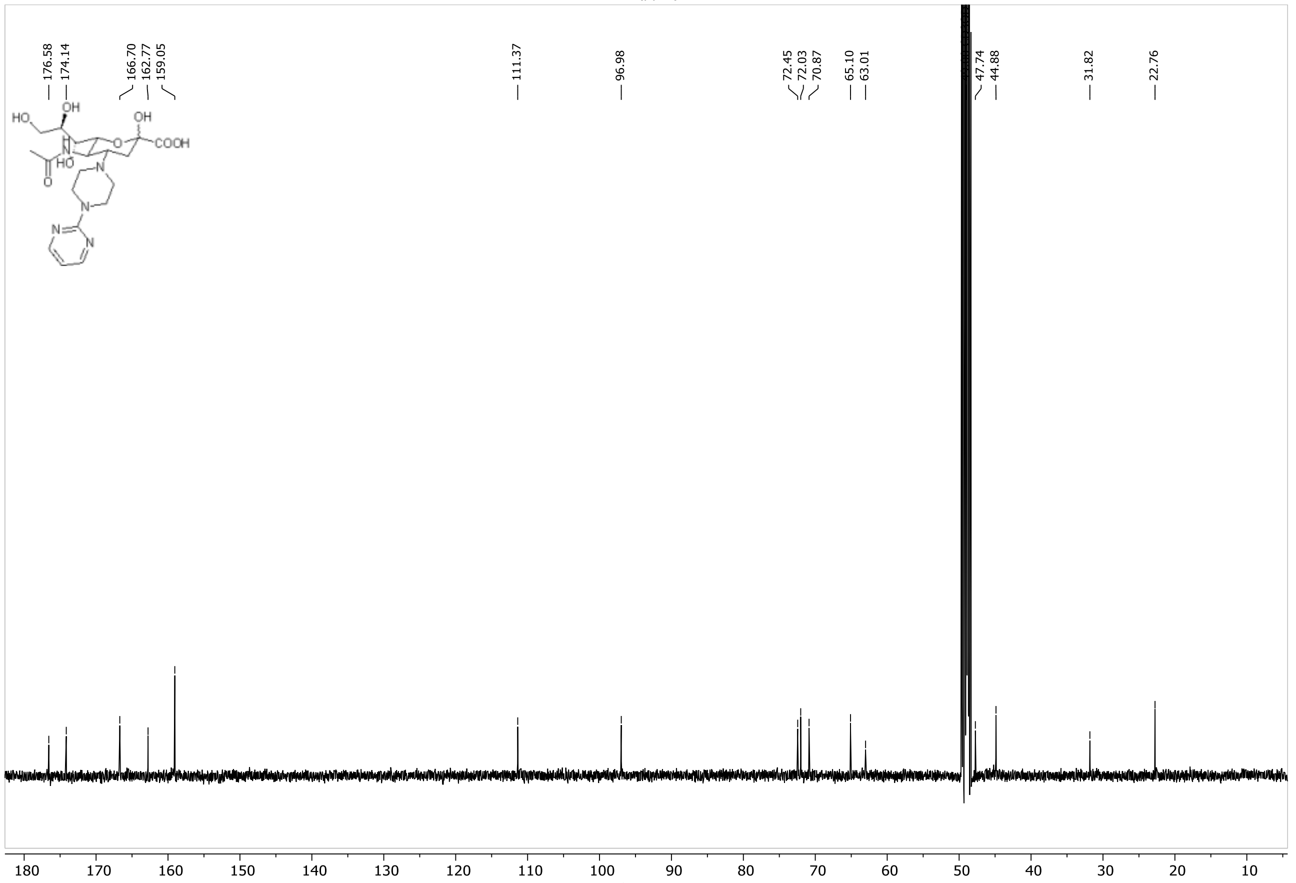

^{1}H- and ^{13}C-NMR spectra of compound 3e

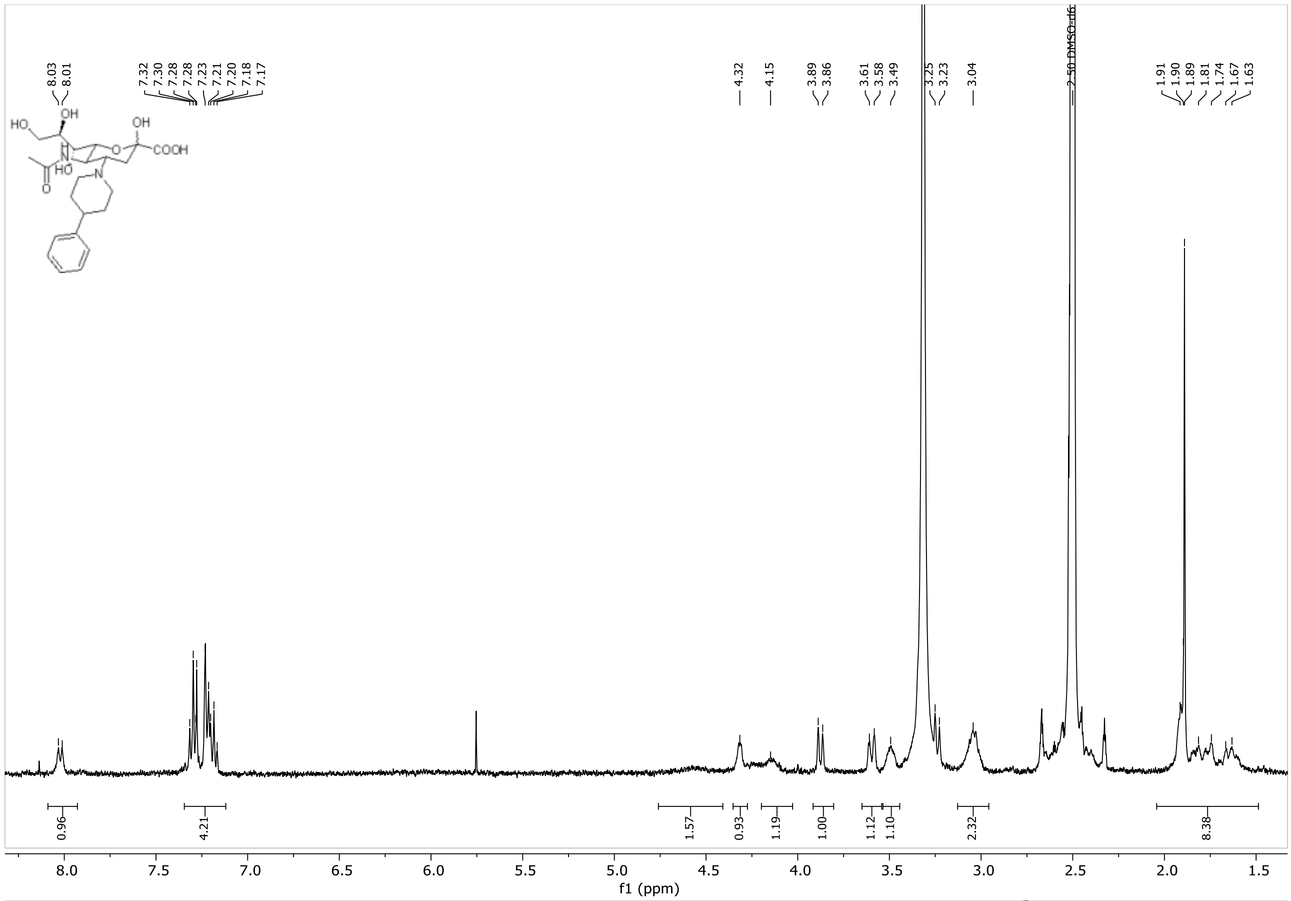

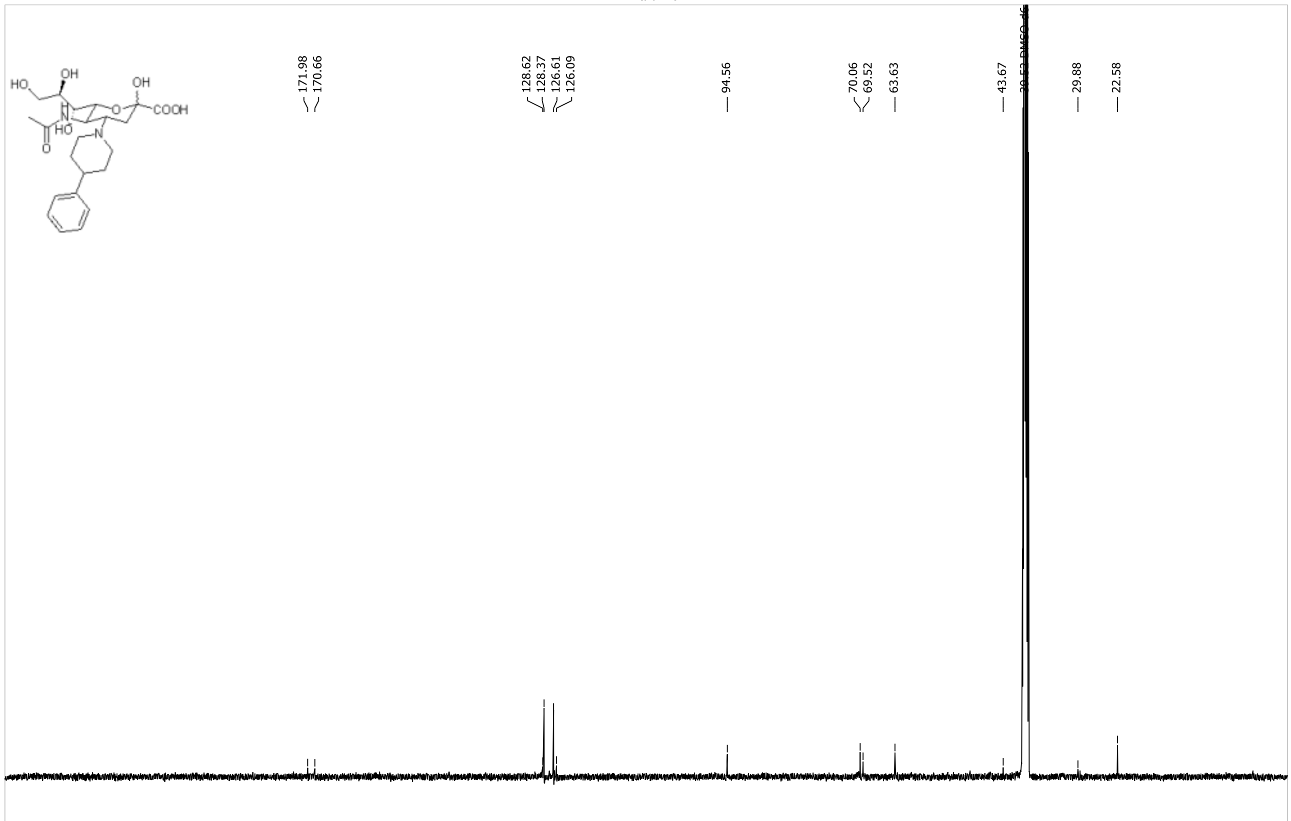

^{1}H- and ^{13}C-NMR spectra of compound 3f

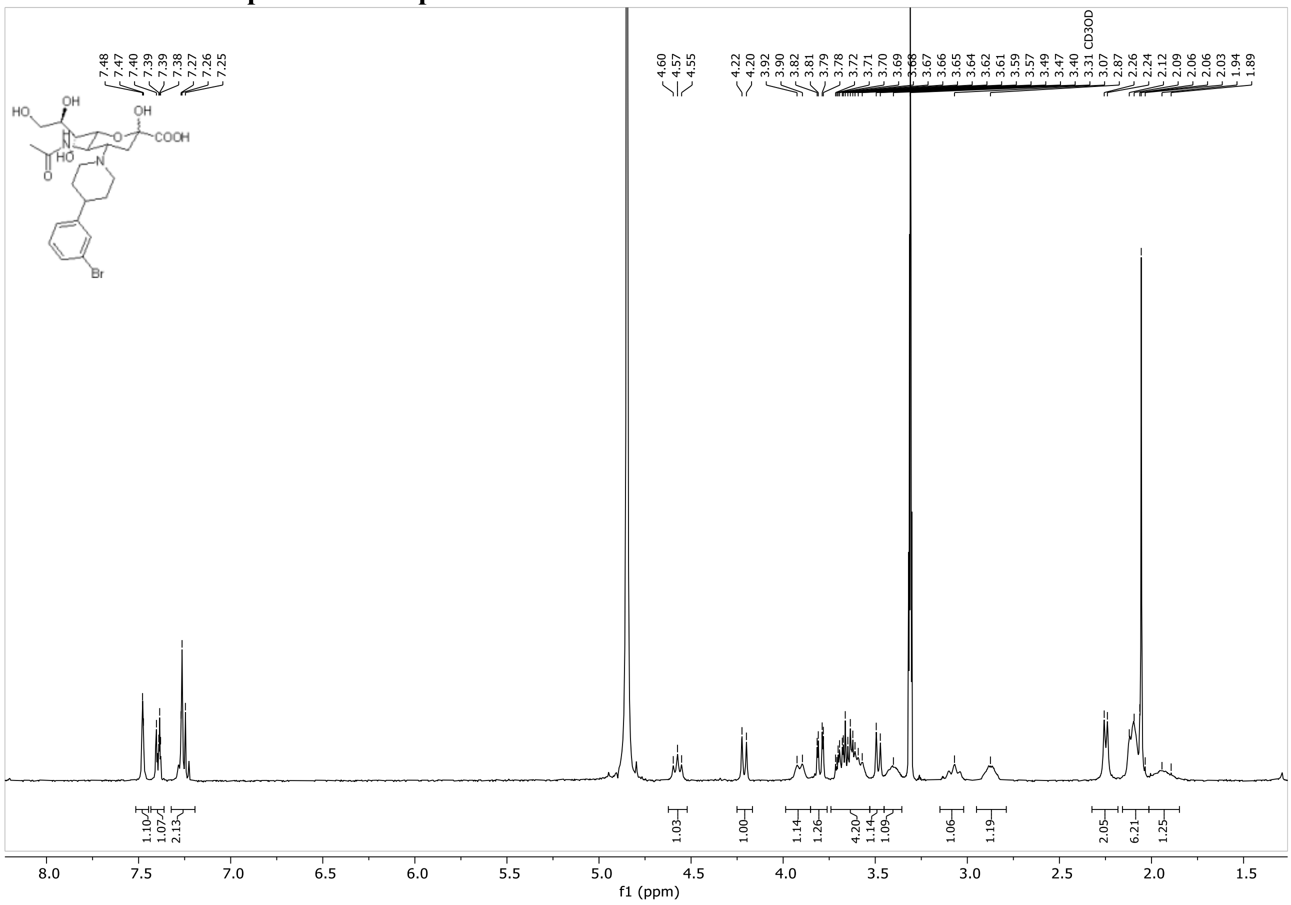

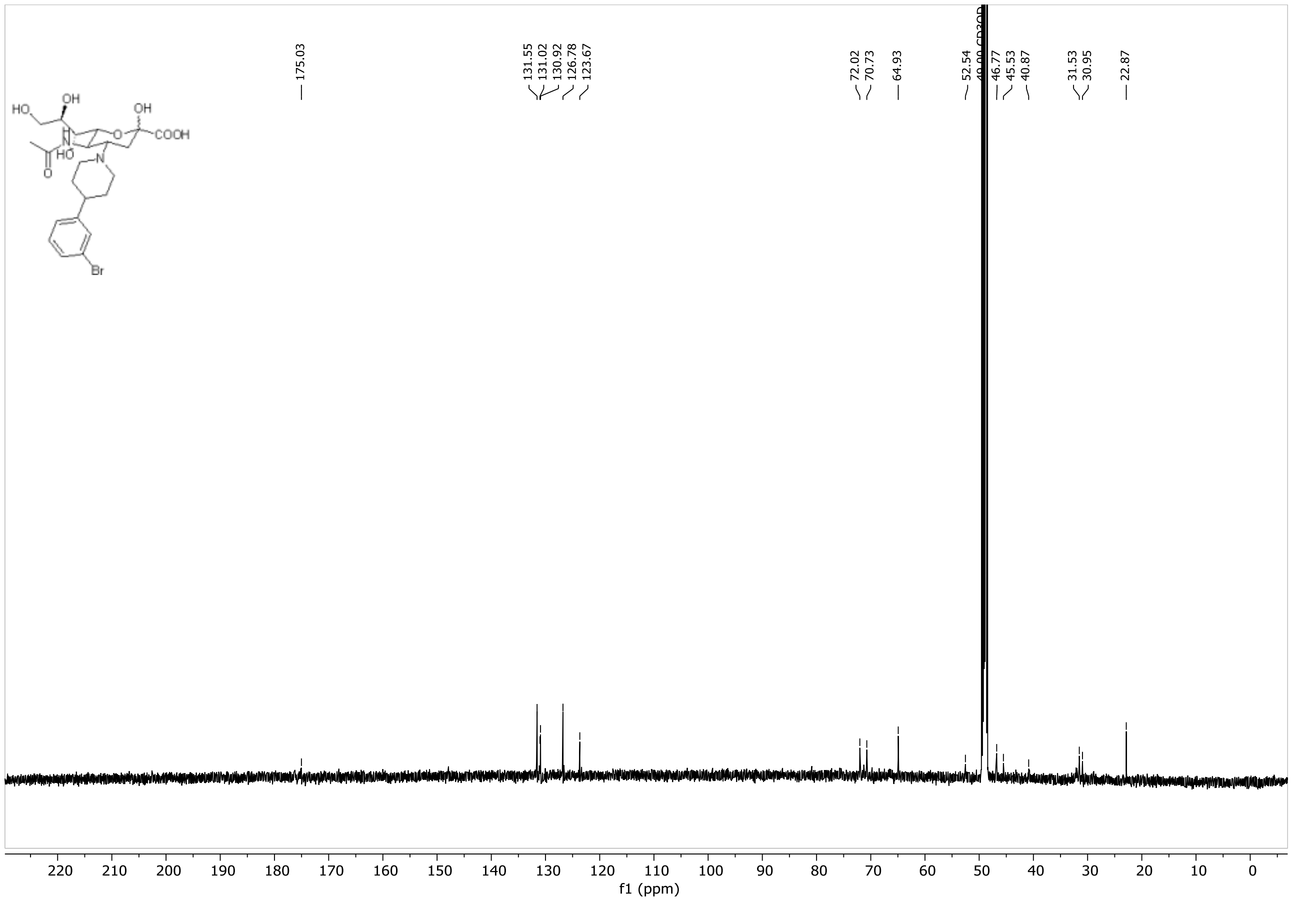

^{1}H- and ^{13}C-NMR spectra of compound 3g

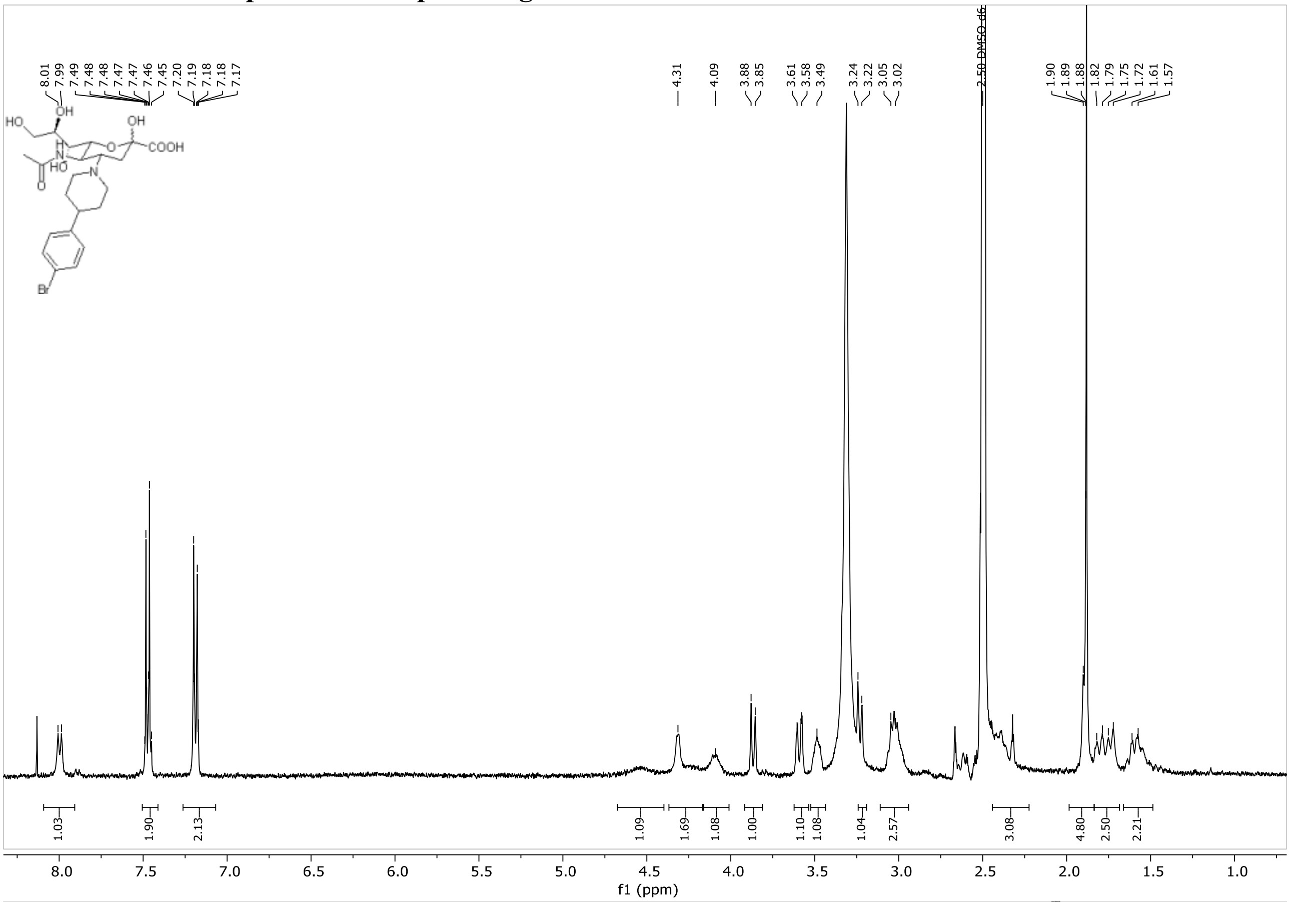

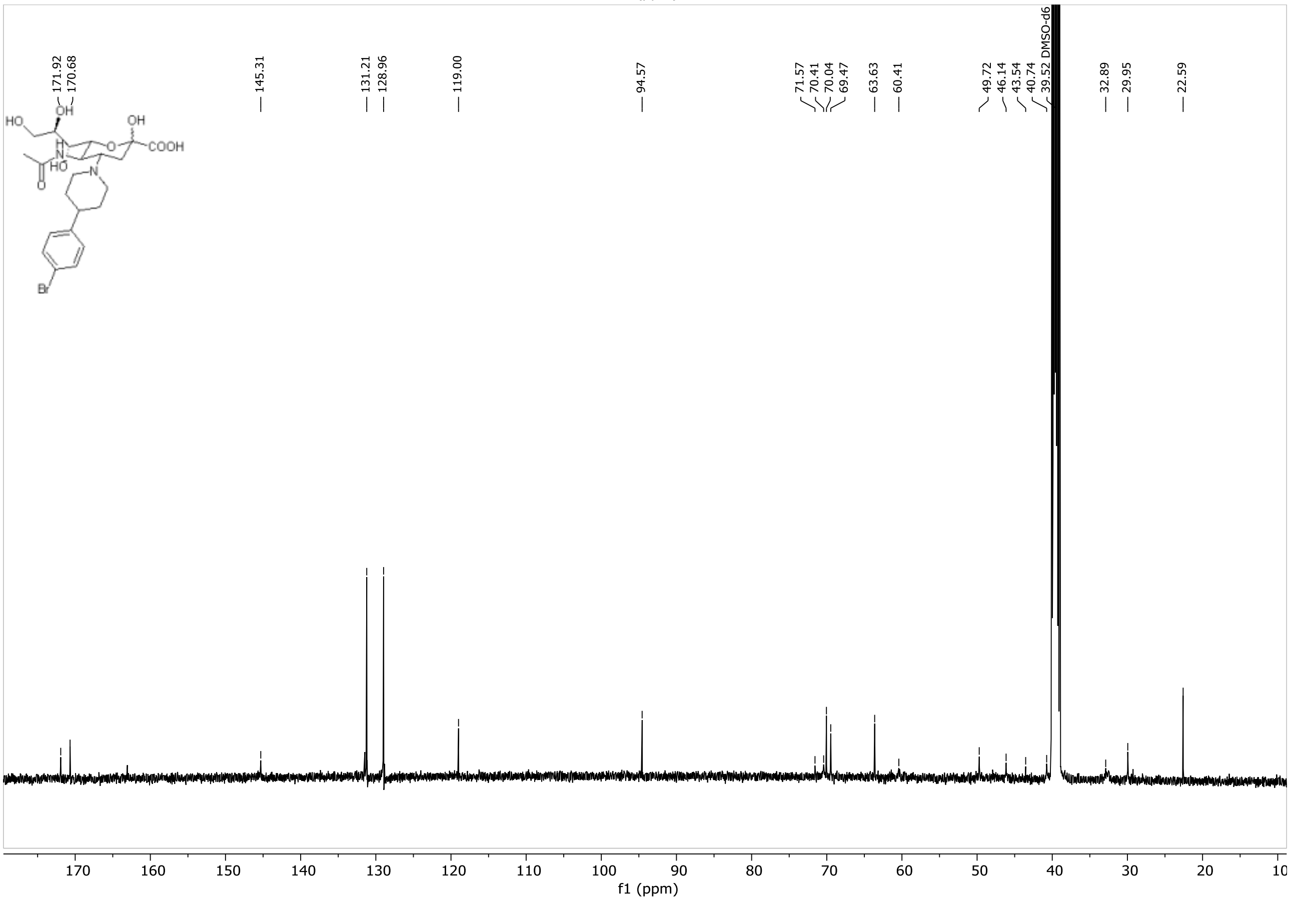

^{1}H- and ^{13}C-NMR spectra of compound 3h

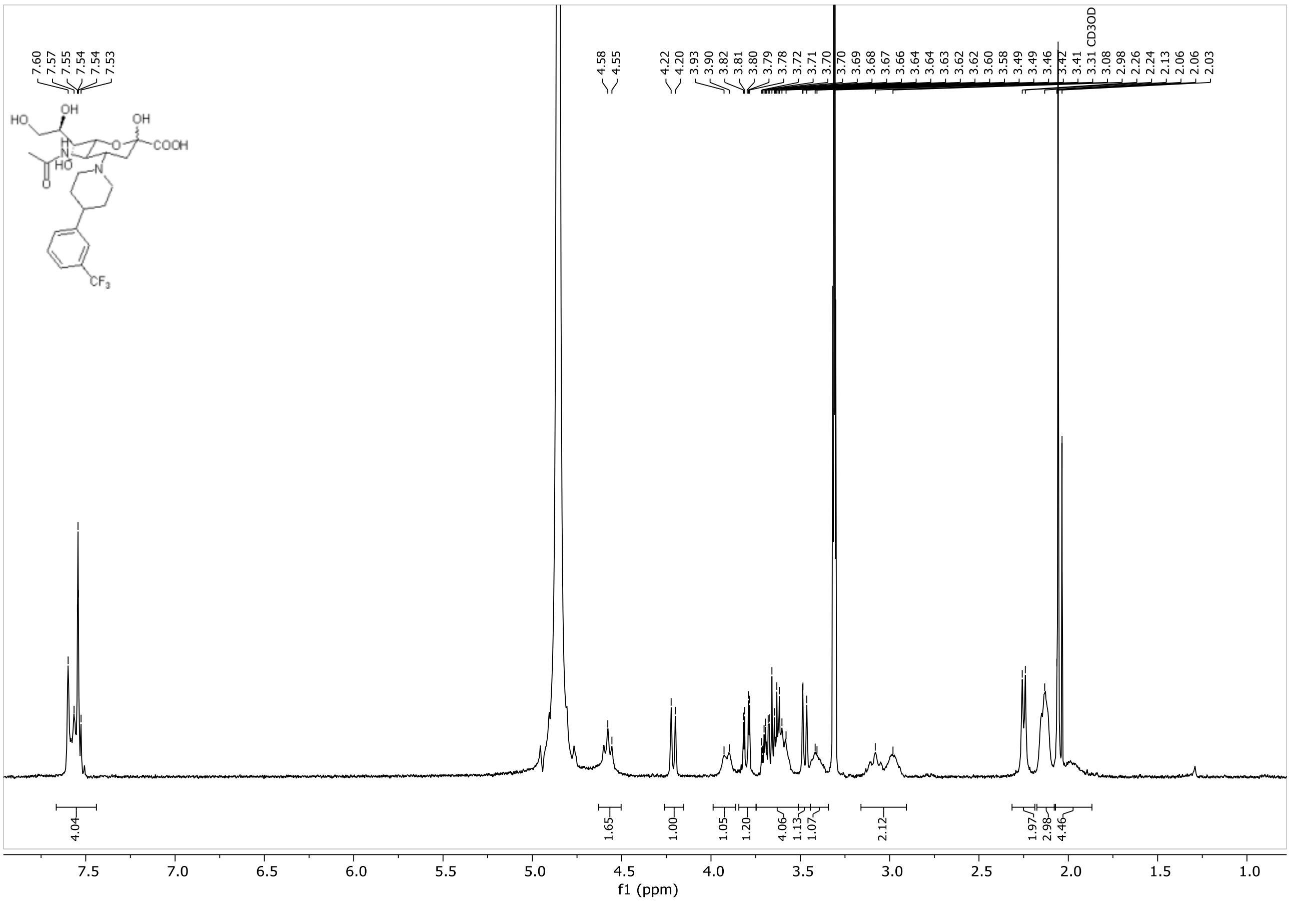

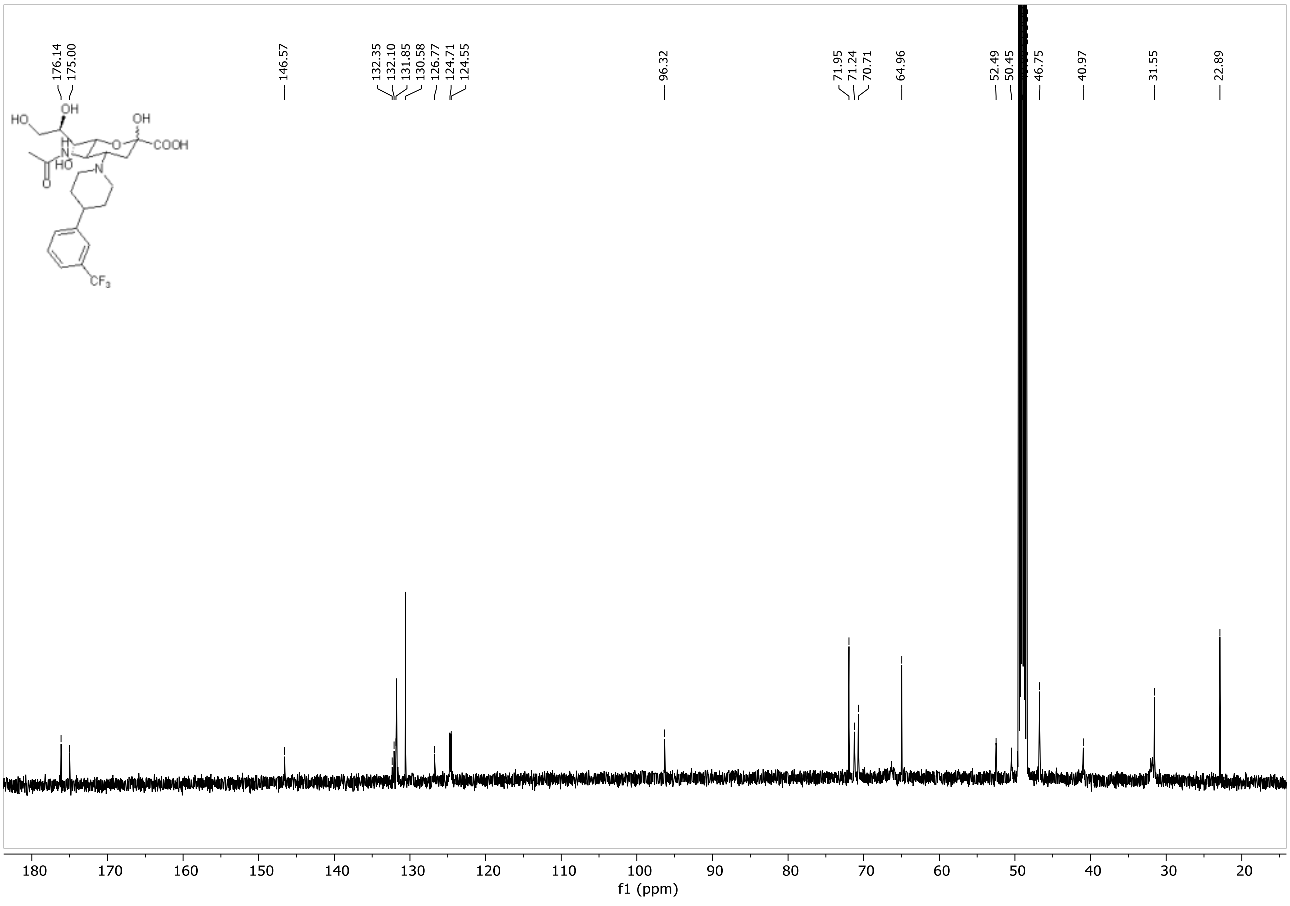

^{1}H- and ^{13}C-NMR spectra of compound 3i

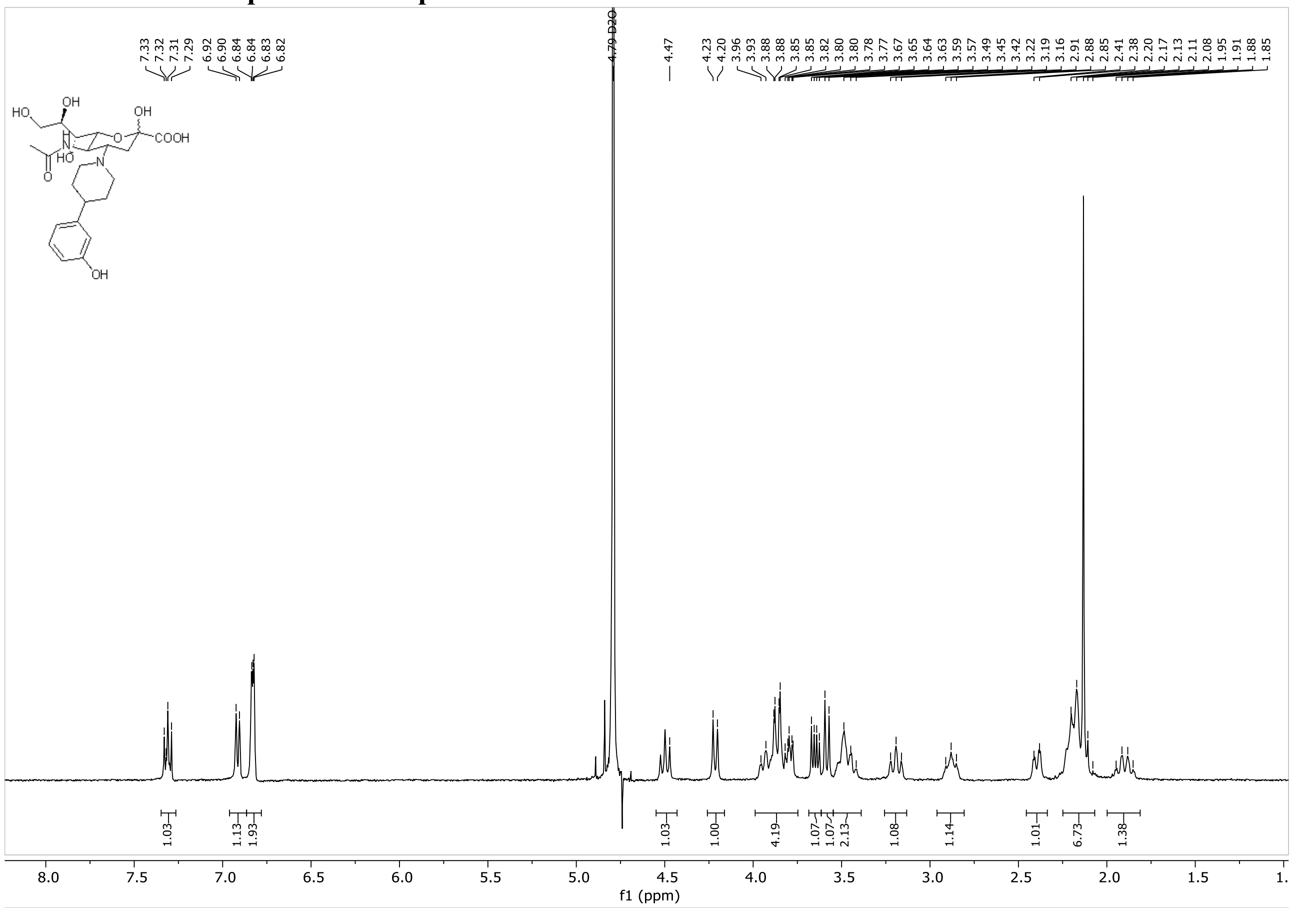

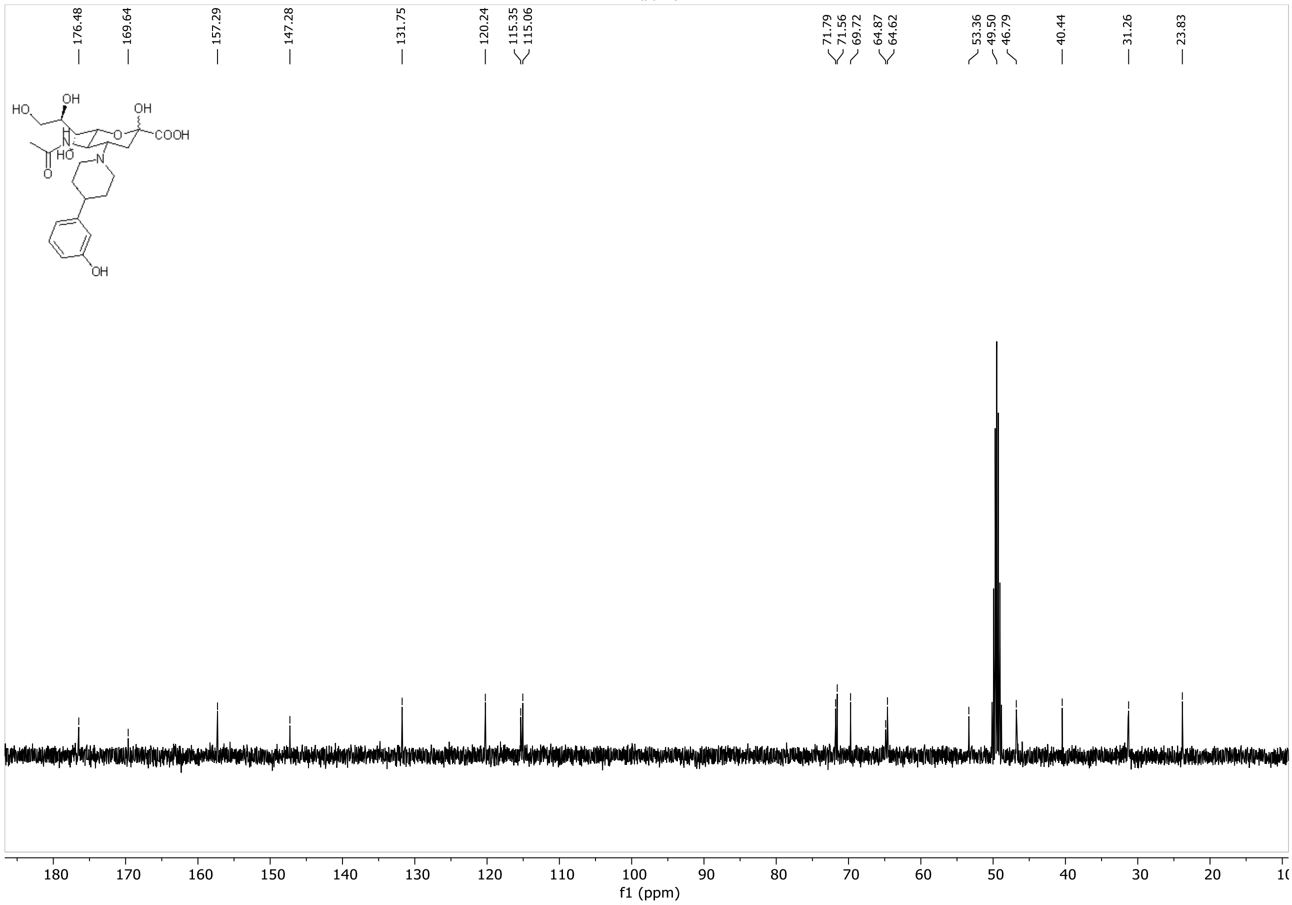

^{1}H- and ^{13}C-NMR spectra of compound 7a

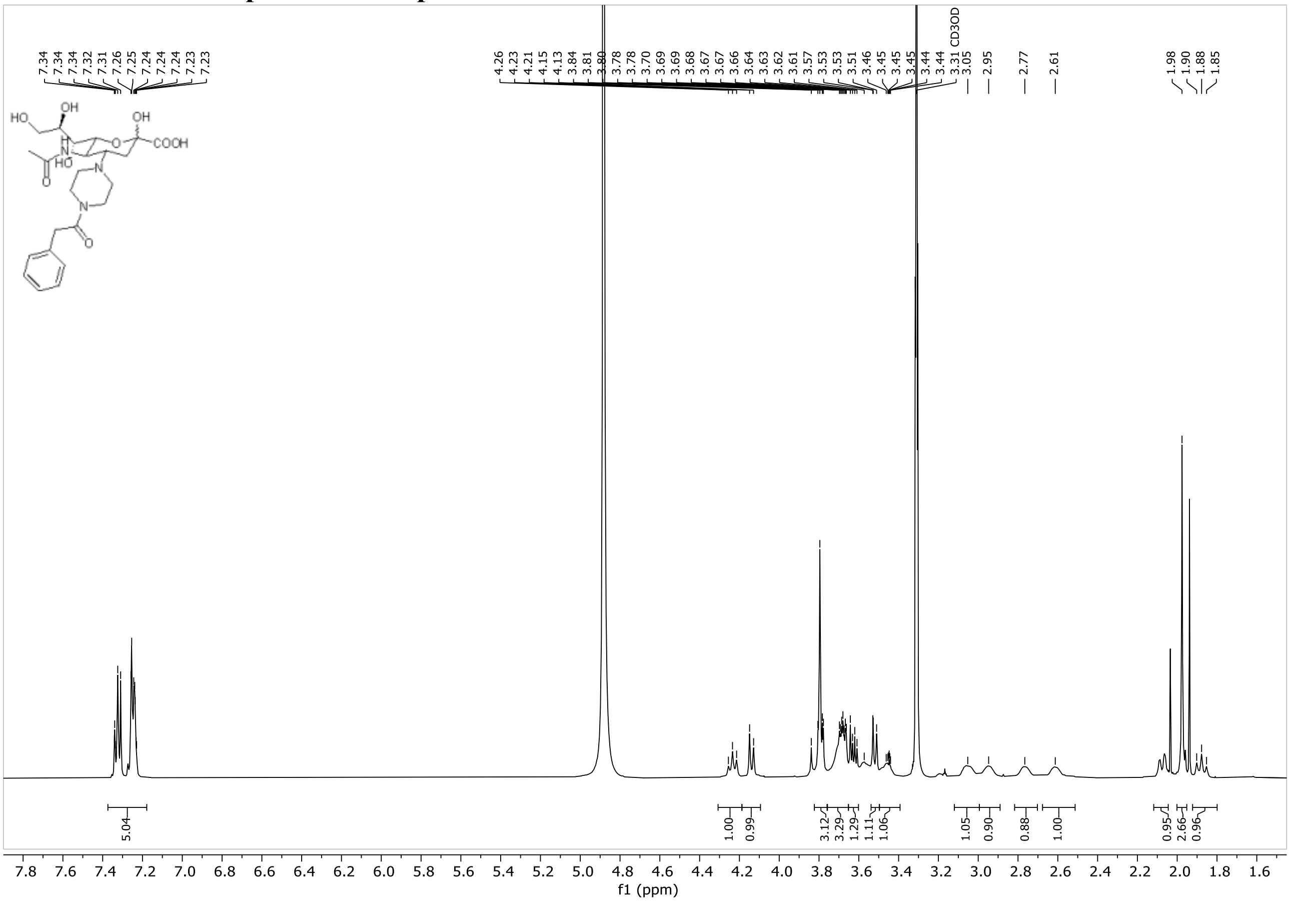

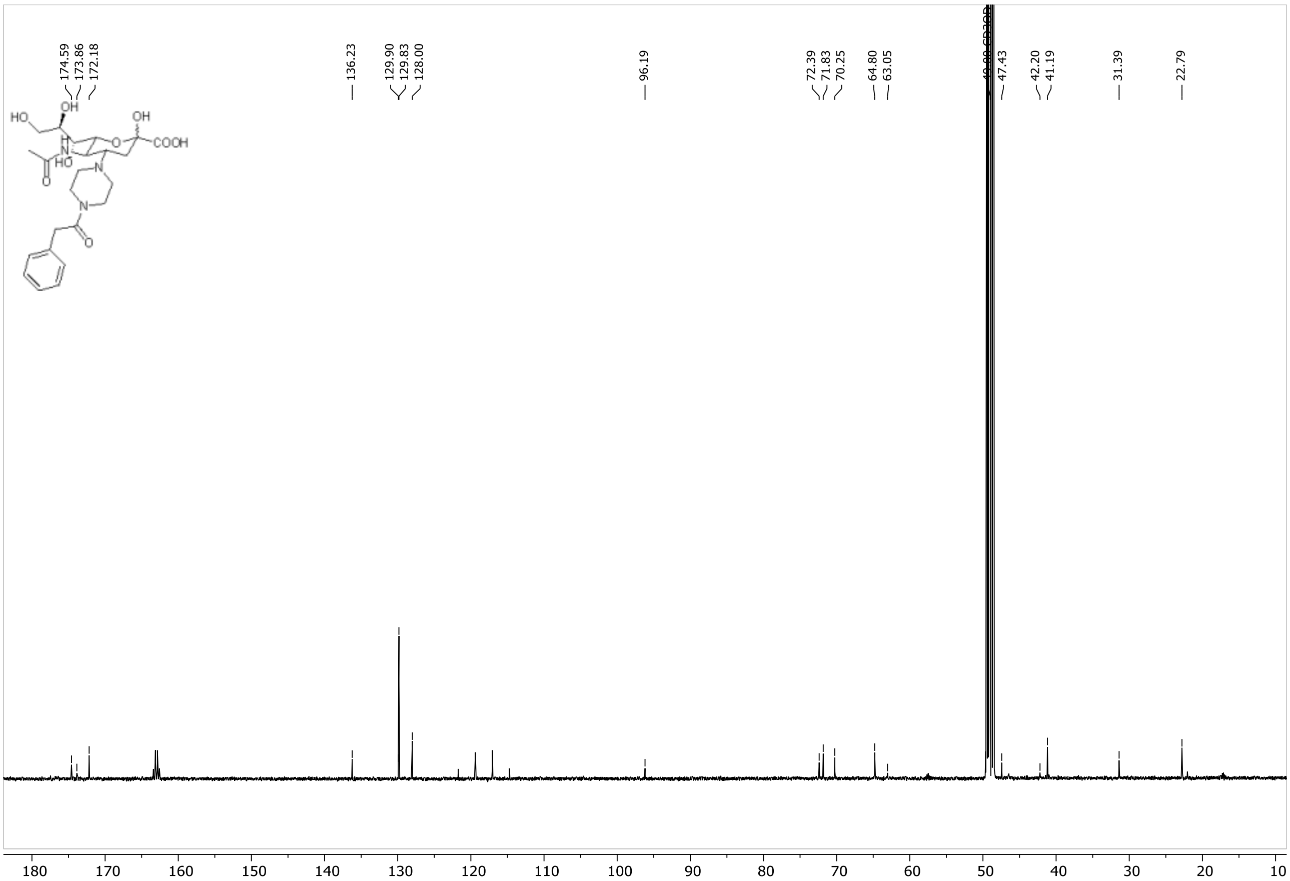

^{1}H- and ^{13}C-NMR spectra of compound 7b

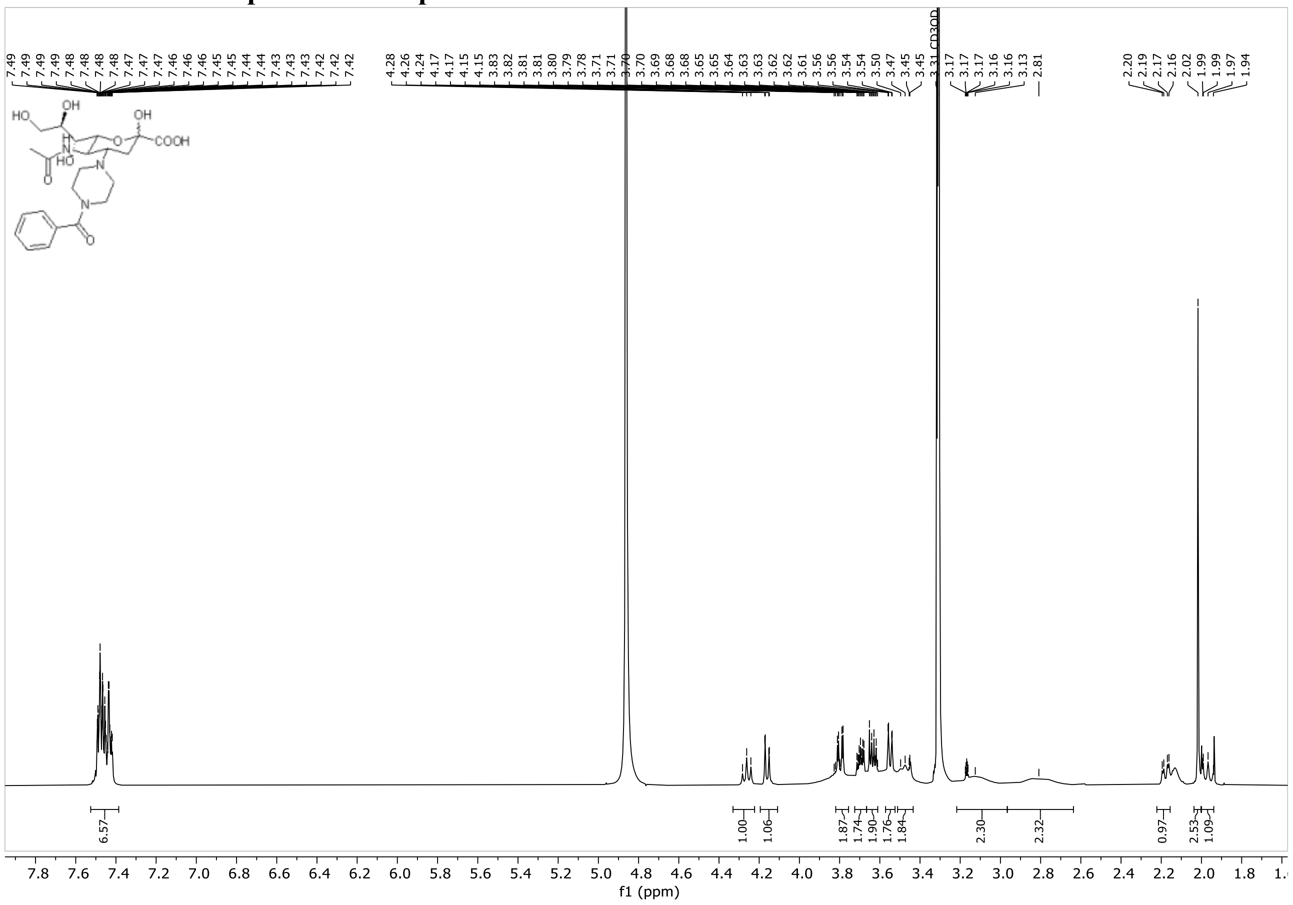

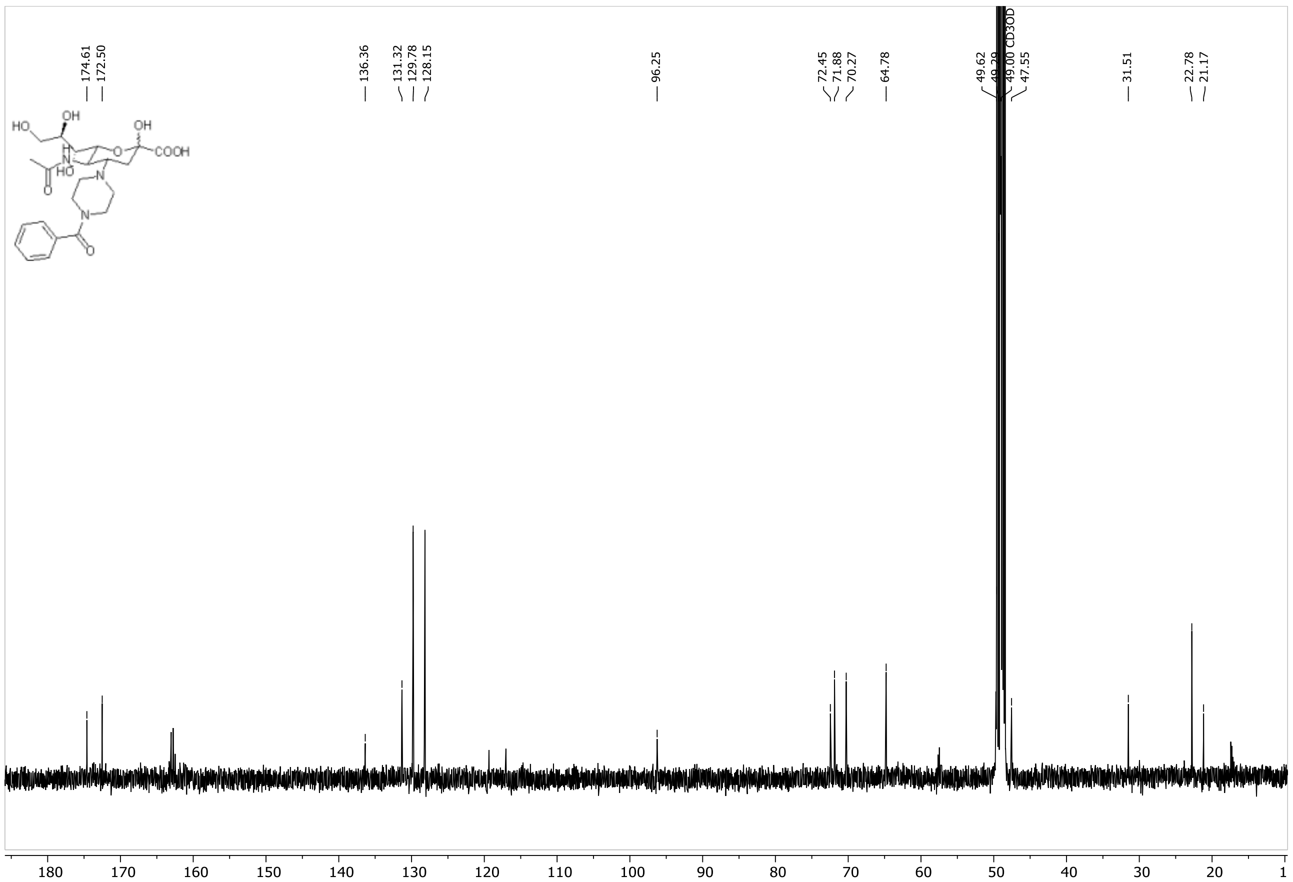

^{1}H- and ^{13}C-NMR spectra of compound 7c

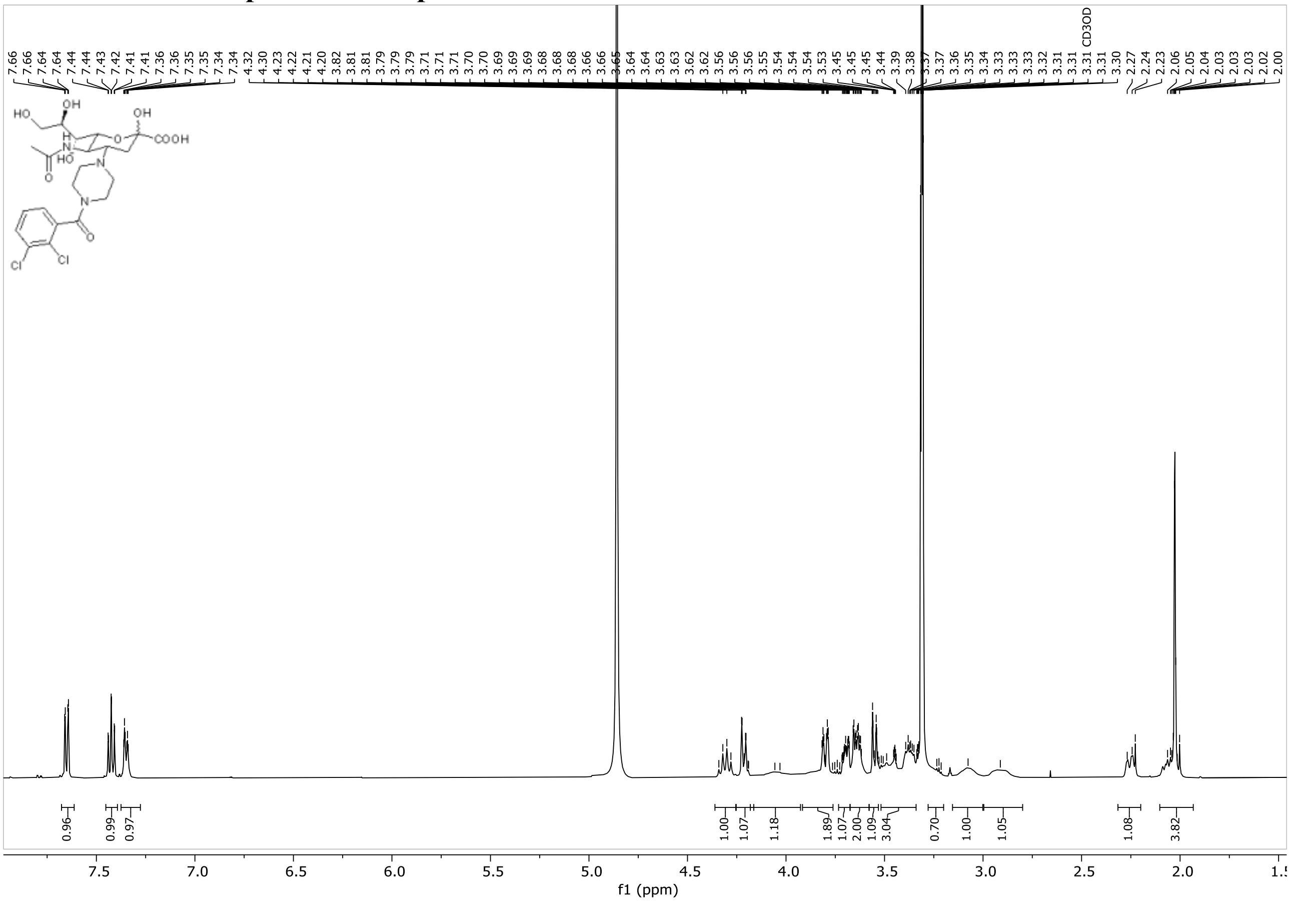

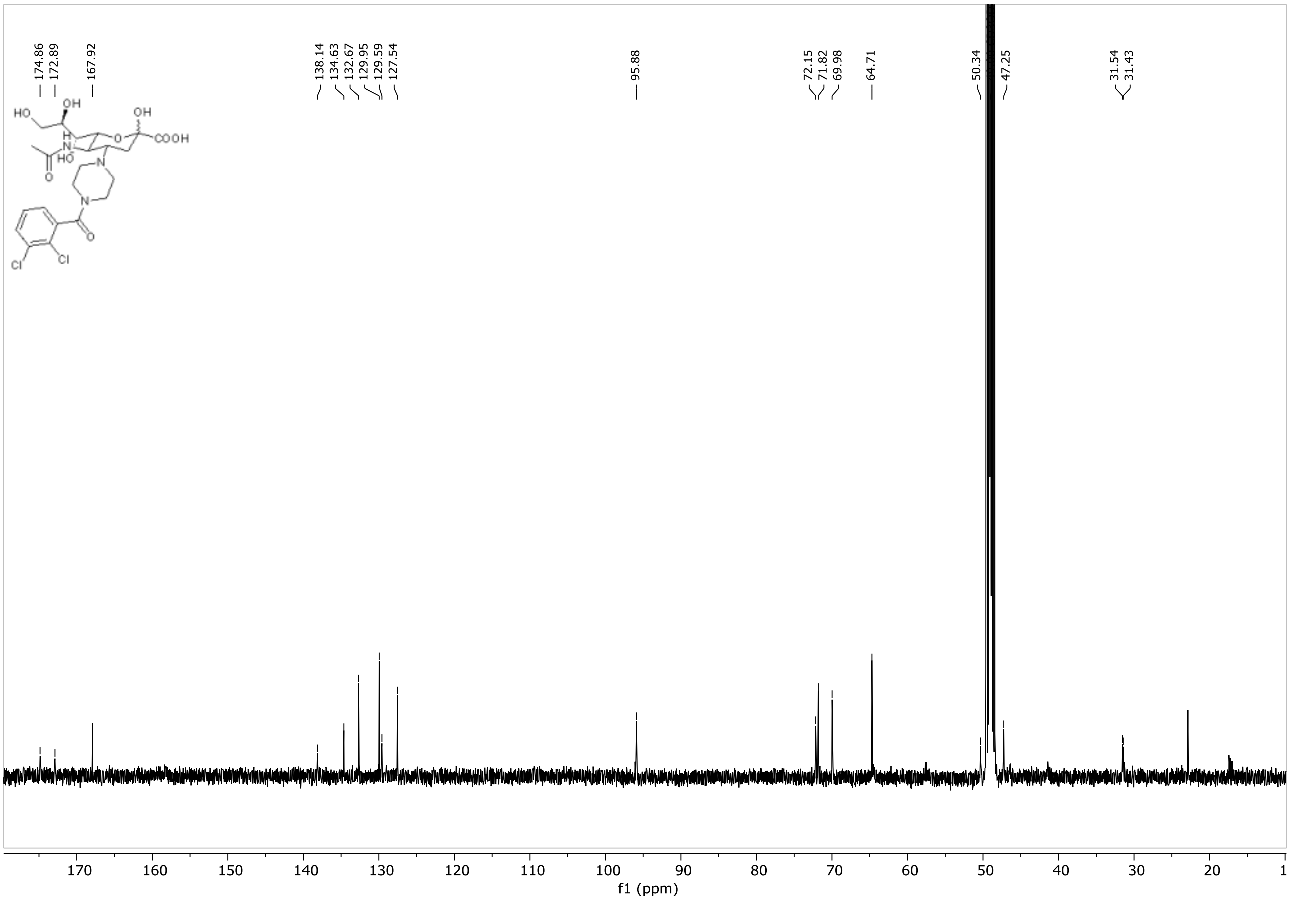

^{1}H- and ^{13}C-NMR spectra of compound 7d

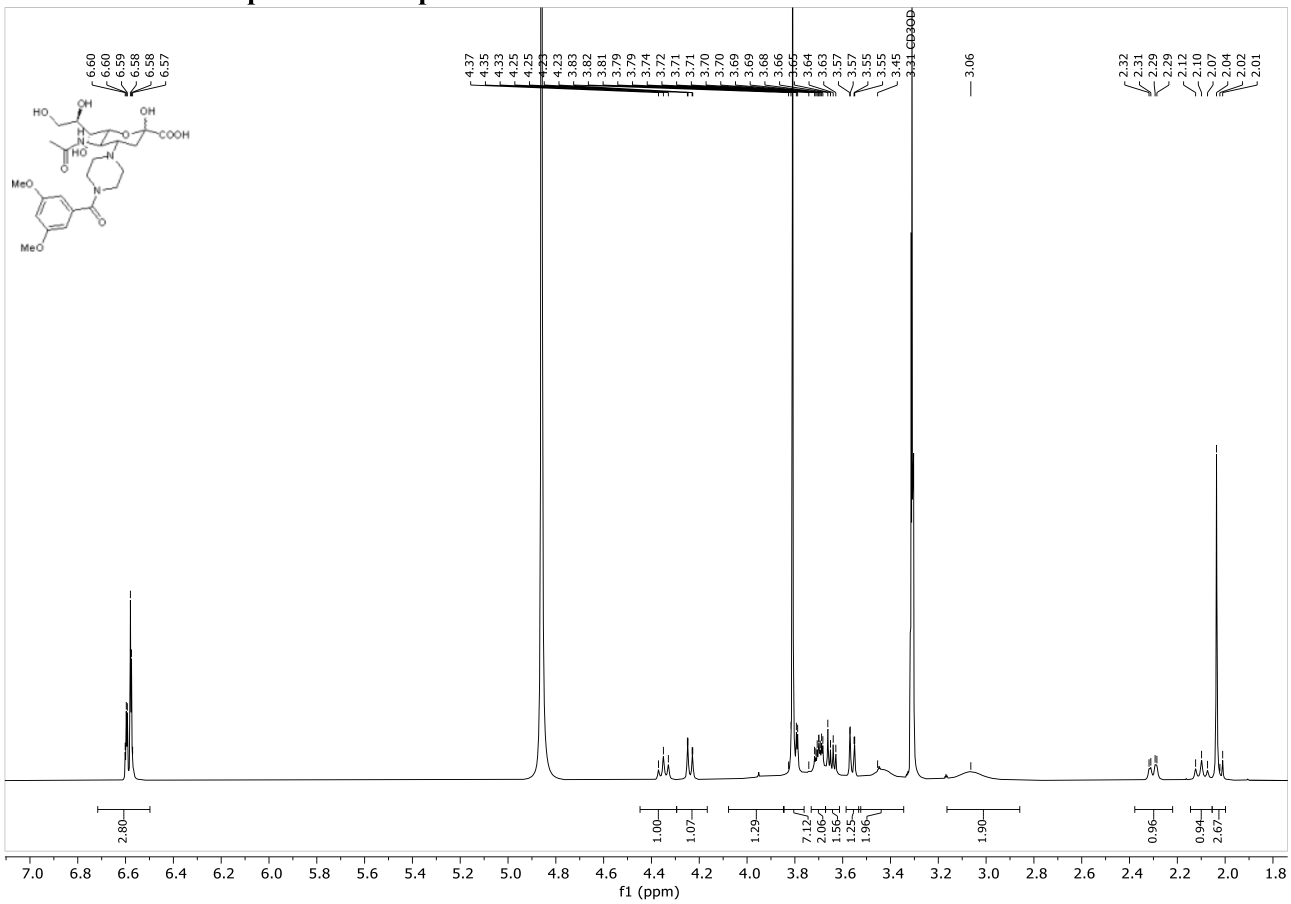

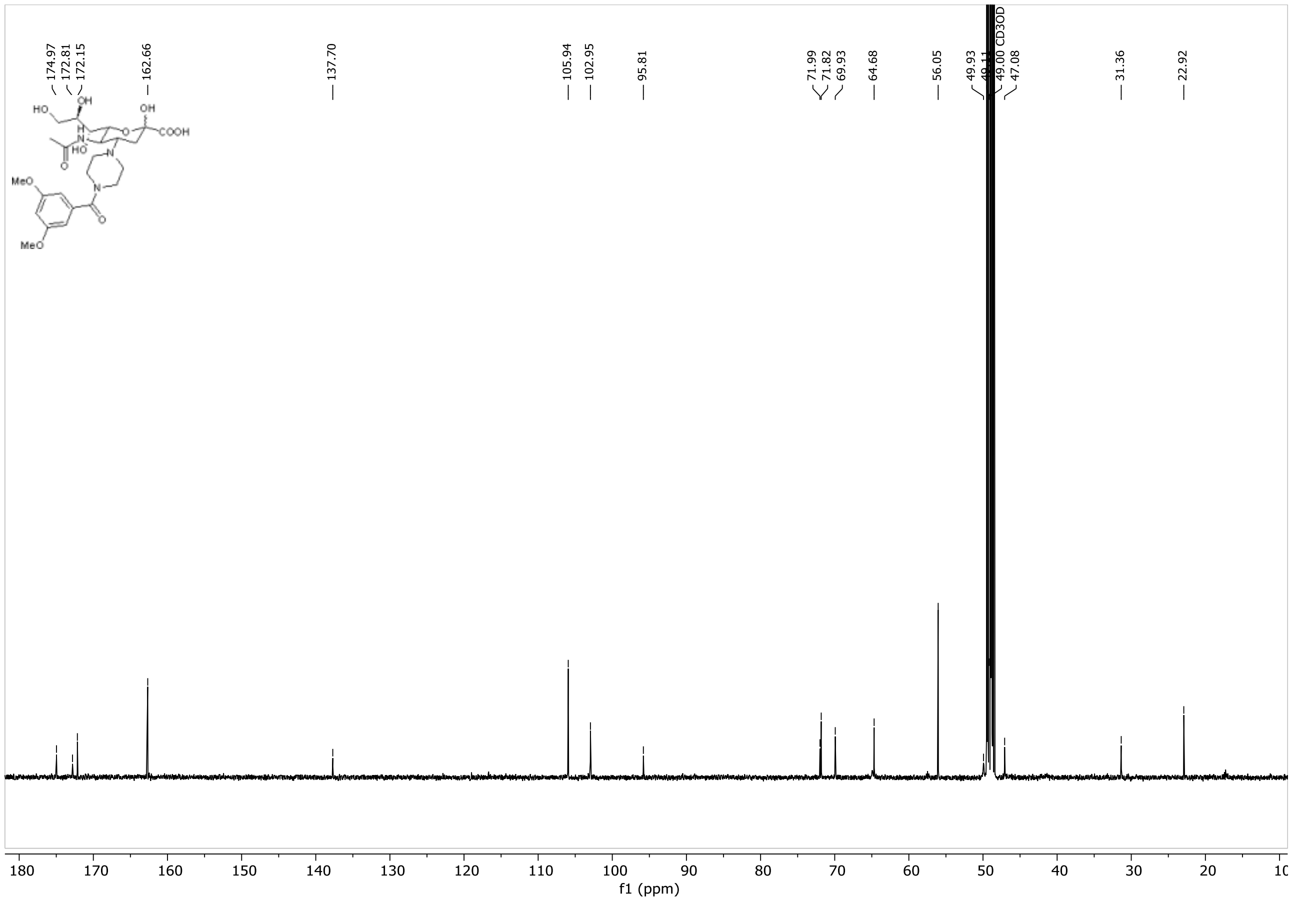

^{1}H- and ^{13}C-NMR spectra of compound 8a

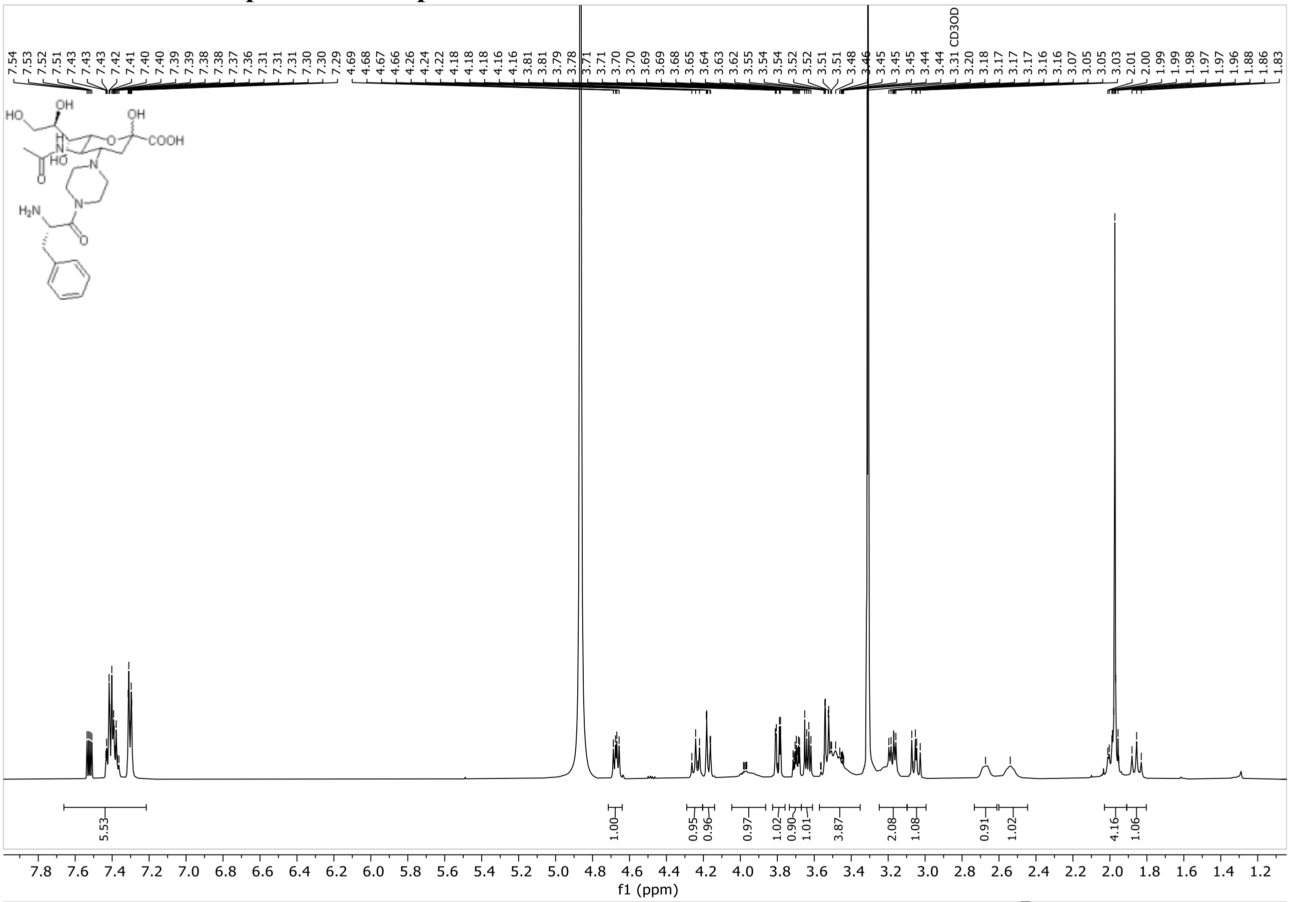

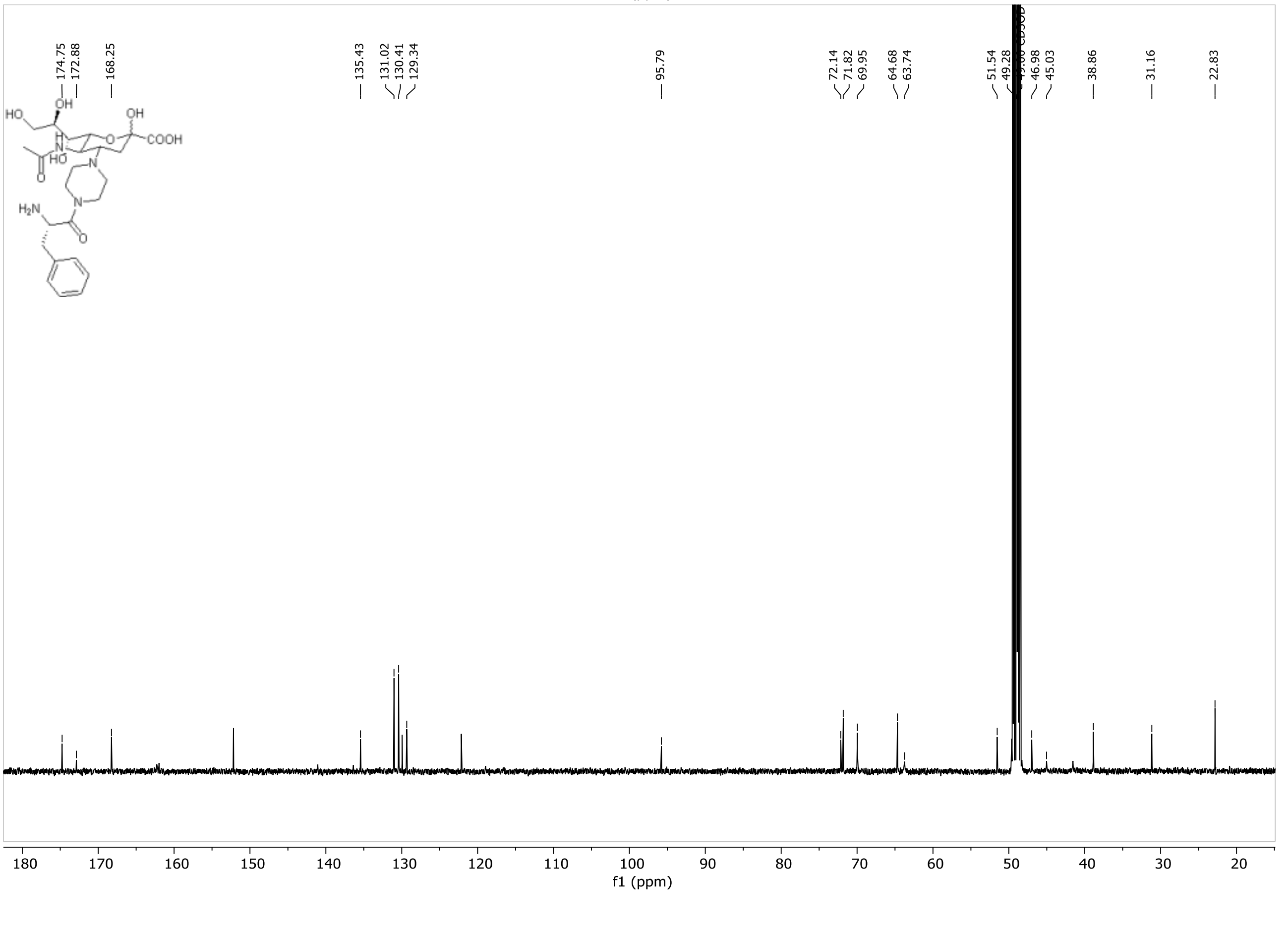

^{1}H- and ^{13}C-NMR spectra of compound 8b

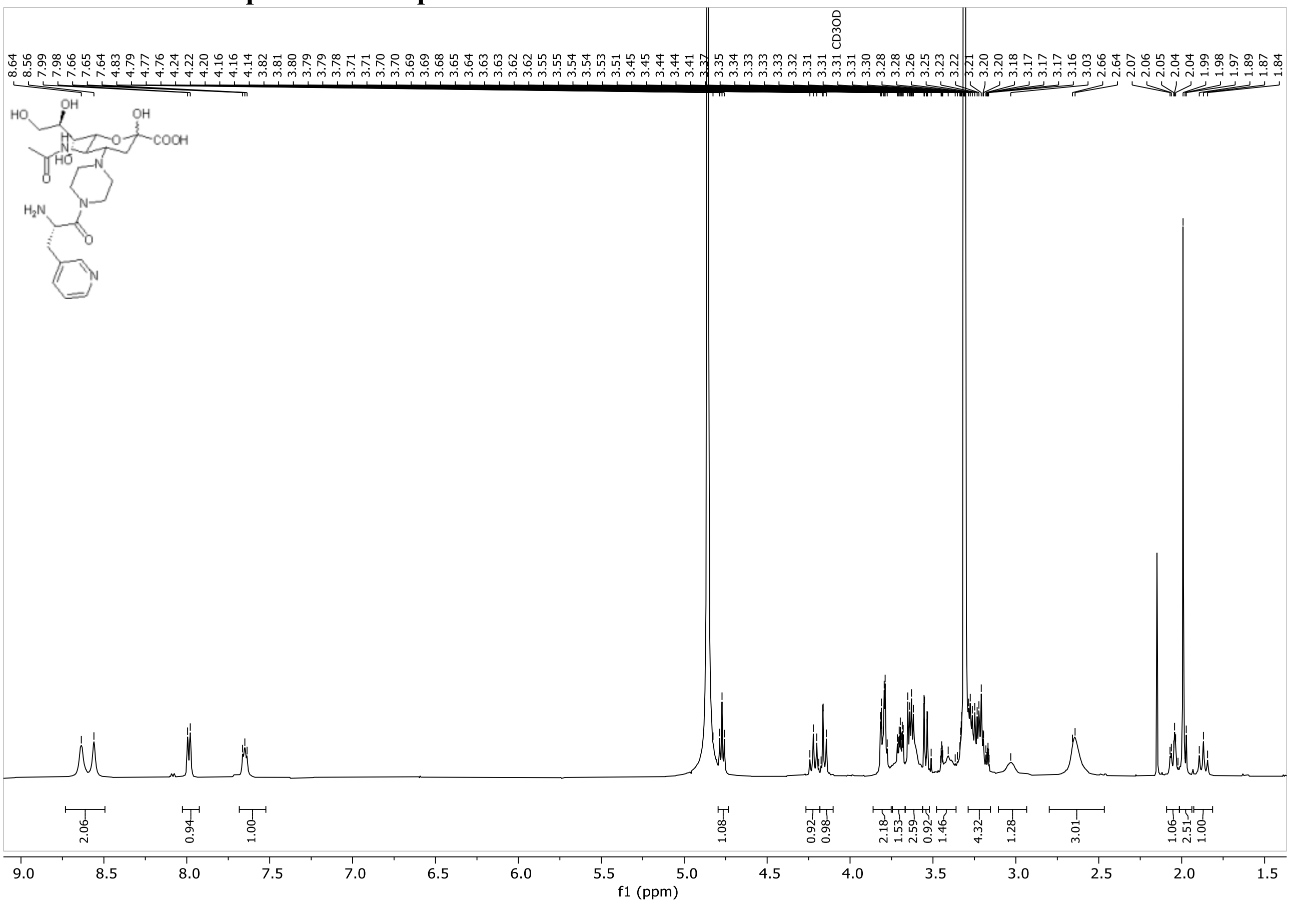

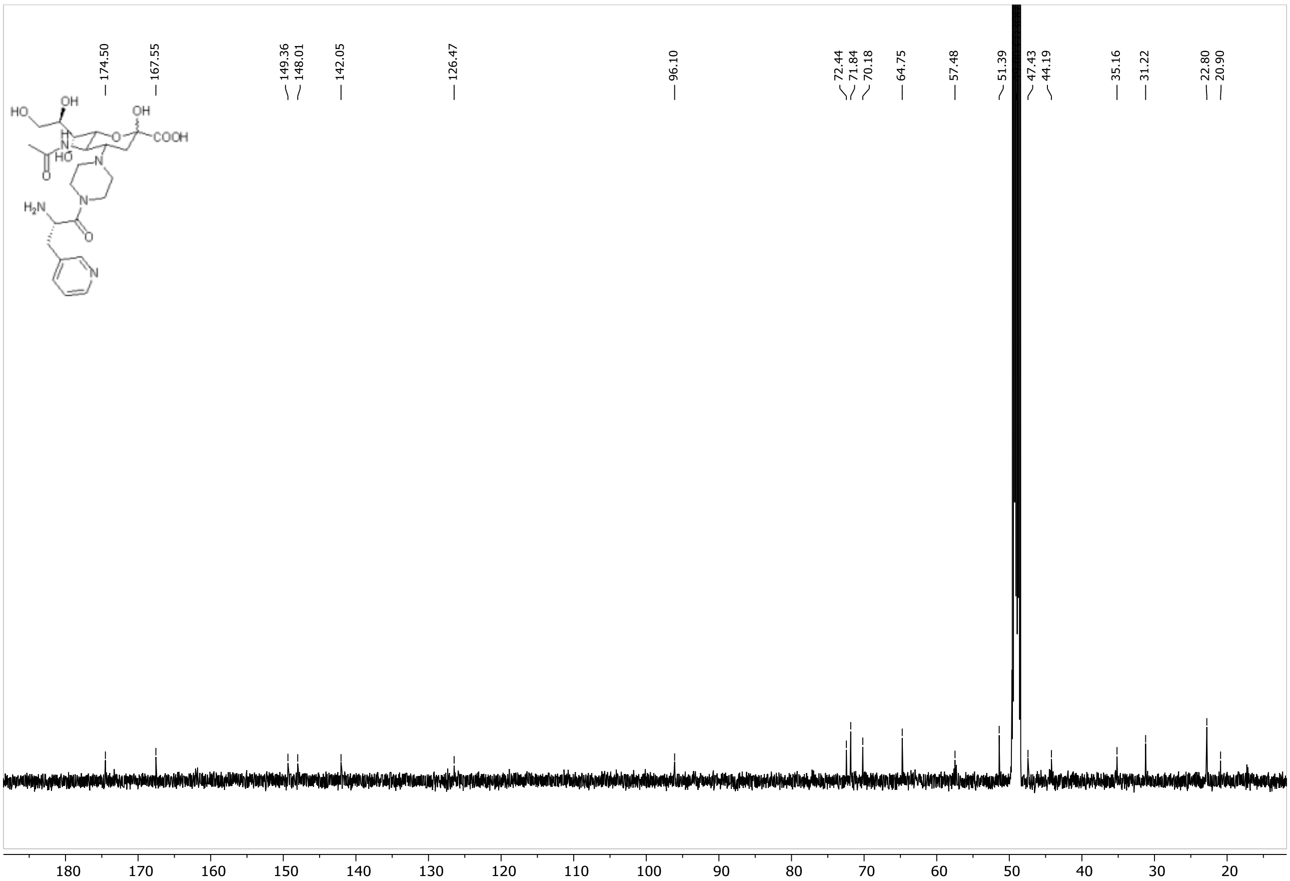

Supplement: Supplementary file 1 — Supporting Information [file CMDC-17-0-s001.pdf]
